# Applying explainable artificial intelligence to interpret supervised ensemble learning models for robust credit card fraud detection

**DOI:** 10.1038/s41598-026-49939-5

**Published:** 2026-05-15

**Authors:** Shimaa S. Awad, Alyaa A. Hamza, Mohamed A. Sobh, Ayman M. Bahaa-Eldin

**Affiliations:** 1 Computer Engineering & Systems Department, Faculty of Engineering, Ain Shams University, Cairo, Egypt; 2 Elsewedy University of Technology, Cairo, Egypt

**Keywords:** Fraud detection, Machine learning, Supervised learning, Explainable Artificial Intelligence (XAI), Financial fraud, Model interpretability, Engineering, Mathematics and computing

## Abstract

As the usage of digital financial transactions continue to swell, it becomes all the more crucial to employ classifiers with machine learning techniques in order to process credit card fraud detection. While complex ensemble models can reach incredible levels of predictive accuracy, the black-box nature of these algorithms often leaves us at a loss, and there hasn’t been much research on how interpretable these high-performing models are across different settings. To contribute to this research gap, this study assesses four supervised learning algorithms (Logistic Regression, Random Forest, XGBoost and LightGBM) based on their predictive performance and applicability with Explainable Artificial Intelligence (XAI) techniques. To optimize the generalizability of their findings, the models were extensively tested and compared against three disparate public credit card transaction datasets. The performance, as measured by different metrics such as accuracy, precision, recall, F1-score and ROC-AUC gives the best results to tree-based algorithms ensembles (especially XGBoost) with linear methods also providing decent improvement over baseline models. Data used as input for training consisted of the top-performing models, and then SHAP (SHapley Additive exPlanations) framework was applied to help identify leading feature importance and interpret complicated predictive output. This study provides a comprehensive outline linking predictive performance to explainability in every combination of models, yielding impactful results for developing effective, transparent and accountable financial security systems.

## Introduction

The rapid proliferation of digital financial transactions has greatly increased the incidence and sophistication of fraud^1^. These evolving threats are simply too much for traditional rule-based systems to keep pace with anymore. So far, thus making machine learning (ML) become an indispensable tool for efficient fraud detection^2–4^. Notably, supervised learning approaches excel at detecting complex pre-existing fraudulent patterns^5–9^.

Nonetheless, there remain considerable gaps in the research limiting actual application of these models in practice. To start, many previous studies have only tested their techniques on a single dataset; this is not very good evidence that they will be effective in different transaction environments^10,11^. To augment the explainability of decisions made by these increasingly sophisticated models^12^, external evaluations have been created that depend on better predictive outputs or more easily explained solutions while still leaving a gap in understanding how to balance predictive accuracy with explainability. Moreover, the currently existing research has a lot of challenges in managing huge imbalance where it becomes difficult to retain Appropriateness without compromising Recall at large skewed fraud datasets^13,14^. Thus, there is an urgent necessity for a holistic framework that addresses the above mentioned challenges by evaluating lots of algorithms across several datasets taking also Explainable Artificial Intelligence (XAI) techniques such as SHAP into consideration in order to make sure we fulfil both high accuracy and interpretability of decisions.

This research addresses the urgent “black box” challenge posed by deploying complex machine learning models, such as deep neural networks or advanced ensembles. Despite such impressive accuracy, these models are often opaque as to why a certain transaction gets flagged. It has caused an increasing interest in Explainable Artificial Intelligence (XAI). However, the existing literature often either treats predictive accuracy and explainability as conflicting goals or focuses on applying XAI to a single model in a single dataset, thus making it difficult to generalize results.

To bridge this gap, using generalization over data realms that constitute a continuous spectrum of high predictive performance with strong interpretability we have introduced a comprehensive and multi-dimensional framework. This study addresses two complex issues (data complexity and model com- plexity): The three datasets display characteristics that cause data complexity in the form of significant class imbalance, different feature spaces and shifting distributions of transactions. While model complexity refers to the non-linear decision boundaries and ensemble interactions learned by Random Forest, XGBoost, and LightGBM. While these models improve detection capabilities, they also add layers of opacity. We list the key technical innovations and primary contributions of this work as follows:

Generalizability across multiple datasets: Most studies rely on single datasets; in contrast, we trained and evaluate four supervised models (Logistic Regression, Random Forest, XGBoost, and LightGBM) over three heavily diverse public credit card transaction data sets. Employing this multi-environment approach to test delivers a strong basis for model consistency, adaptability and real-world usability.

Cross-Model Explainability Comparison: We extend the current state of the art in XAI by providing a unique, side-by-side comparison using SHAP (SHapley Additive exPlanations) technology across a range of high-performing models. This approach helps us understand how different algorithmics structures (such as gradient boosting vs. bagging) focus and interpret similar fraudulent structures differently.

Financial Deployment for Precisely Actionable Precision-Recall Optimization: Accounting for an imbalanced fraud domain we delve into the precision-recall trade-off. By connecting these metrics to XAI outputs, we deliver actionable principles based on a data-oriented approach for finance institutions to select models that meet their unique risk tolerance and business realities.

Real-World Deployment Framework: To contrast the theory of prioritization with practical implications, we propose a formal deployment framework. It addresses key operational challenges such as inference latency, system scalability (micoroservice architecture), monitoring model performance for concept drift detection and compliance (GDPR Article 22, PSD2). This is all to ensure that our models are fully ready for real-world production environments^15,16^.

Beyond improving the state of research, this integrated approach provides a practical guide for strengthening efficient, transparent and accountable financial security systems.

The manuscript is organized as follows: Section “Related work” outlines related works, the section “Methodology” describes the methodology and datasets, the section “Experimental results” presents the results and XAI analysis while finally, the section “Conclusions, limitations, and future work” concludes with a summary.

## Related work

The field of financial fraud detection has come a long way, moving from old-school rule-based systems to cutting-edge artificial intelligence frameworks. In this section, we take a close look at the latest literature, organizing it by the advancements in methodology. Unlike earlier reviews that simply described the findings, this analysis brings together recent top-tier research from 2023 to 2026, examining the effectiveness, architectural trade-offs, and real-world challenges of different approaches. These include cost-sensitive learning, deep sequential models, graph-based networks, and autoencoders.

### Traditional supervised models and cost-sensitive learning

In the past, supervised learning algorithms like Random Forest, Support Vector Machines (SVM), and Logistic Regression were the go-to methods for detecting credit card fraud^5–7^. These models can achieve impressive accuracy on balanced datasets, but they really struggle when faced with the severe class imbalance that’s common in real-world financial transactions.

To tackle this significant issue, recent studies have started focusing on Cost-Sensitive Learning (CSL). Instead of treating all misclassifications the same, CSL places a heavier penalty on false negatives (which means missing a fraud case) compared to false positives (which are false alarms). For instance, Xiao et al. (2025) showed that using example-dependent cost-sensitive frameworks can greatly enhance the financial outcomes by aligning the loss function with the actual amounts of transactions^17^. Likewise, Singh and Jain (2022) introduced a cost-sensitive weak learner ensemble that effectively addresses class imbalance without depending on synthetic oversampling methods like SMOTE, which can sometimes add unnecessary noise^18^. A thorough review of these studies indicates that while CSL does boost recall, it often demands a lot of hyperparameter tuning to avoid a problematic increase in false positive rates, underscoring the importance of dynamic thresholding in real-world applications.

### Deep learning architectures: LSTMs and transformers

The ever-changing landscape of credit card transactions has led to the rise of deep learning models tailored for sequential data. Long Short-Term Memory (LSTM) networks have been widely used to capture the long-term patterns in how users spend their money. Researchers like Benchaji et al. (2021) and Akour et al. (2025) took traditional LSTMs a step further by incorporating attention mechanisms. This innovation allows the network to zero in on particularly suspicious transactions within a sequence, rather than treating all past data with the same level of importance^19,20^.

Recently, there’s been a shift towards Transformer-based architectures. Initially created for natural language processing, Transformers use self-attention to analyze entire transaction sequences simultaneously, which helps to bypass the sequential limitations of LSTMs. Yu et al. (2024) demonstrated the power of advanced Transformer models on tabular transaction data, achieving impressive predictive accuracy by effectively capturing complex, non-linear interactions between features^21^. A systematic review by Chen et al. (2025) highlights that while Transformers excel over RNNs/LSTMs in terms of accuracy and parallel processing, their significant computational demands and need for large datasets still pose challenges for real-time applications in banking systems^1^.

### Graph-based fraud detection

Fraudulent activities, especially those linked to organized crime and money laundering, often rely on well-coordinated networks of accounts. Unfortunately, traditional tabular models struggle to capture these complex relationships. This is where Graph Neural Networks (GNNs) come into play as a cutting-edge solution. By treating accounts as nodes and transactions as edges, GNNs can gather localized neighborhood information to spot unusual structural patterns.

In a thorough review from Q1, Motie and Raahemi (2024) found that GNNs are particularly effective at uncovering intricate relational fraud that traditional models often overlook^22^. Recent advancements have introduced Heterogeneous Graph Neural Networks (HGNNs), which can handle various types of nodes (like users, devices, and merchants) and edges all at once. For instance, Sha et al. (2025) employed HGNNs with graph attention to pinpoint fraudulent subgraphs within extensive transaction networks^23^. Additionally, Tian et al. (2023) presented adaptive sampling GNNs to tackle the computational challenges of processing large-scale graphs in real-time^24^. While GNNs offer unmatched insights into relationships, their practical use can be limited by the delays in real-time graph construction and the “oversmoothing” issue, where node embeddings become too similar in dense networks.

### Unsupervised anomaly detection and autoencoders

With the limited availability of labeled fraud data and the swift rise of new attack methods, unsupervised anomaly detection has become a vital area of research. Autoencoders (AEs) and Variational Autoencoders (VAEs) are deep generative models that are trained solely on legitimate transactions to understand what “normal” behavior looks like. When it comes to inference, fraudulent transactions tend to show a high reconstruction error, which helps identify them as anomalies.

Misra et al. (2020) showcased how effective AEs can be in detecting credit card fraud without needing prior labels^25^. Since then, there have been significant improvements in this field. For example, Shi et al. (2025) introduced an attention-based balanced VAE that adeptly handles data imbalance while enhancing the separation of latent distributions^26^. Additionally, hybrid architectures are becoming more popular; Mienye et al. (2025) merged VAEs with Graph Attention Networks to simultaneously capture both feature-level anomalies and structural irregularities^27^. However, a major drawback of autoencoder-based systems is their tendency to produce a high false-positive rate in fast-changing financial environments, where legitimate but infrequent purchases (like travel expenses) are often misidentified as reconstruction anomalies.

### Explainable AI (XAI) and privacy-preserving models

In the realm of deep learning, as models become more intricate, their “black-box” nature often clashes with stringent financial regulations like GDPR Article 22. This has made Explainable AI (XAI) an essential part of modern fraud detection strategies. For instance, Sai et al. (2023) combined SHAP (SHapley Additive exPlanations) with deep neural networks to enhance transparency at the feature level for individual predictions^28^.

At the same time, concerns about data privacy have led to the rise of Federated Learning (FL). Aljunaid et al. (2025) introduced an XAI-driven FL framework that enables multiple banks to work together in training fraud detection models without needing to share sensitive customer data^29^. However, while this approach seems solid in theory, merging XAI with FL can lead to considerable computational demands and communication delays, creating a delicate balance between transparency, privacy, and the speed of real-time execution.

### Synthesis and comparative analysis

In order to give you a clear picture of the current research landscape, Table 1 lays out a comparative evaluation of the key methodological approaches. Rather than just providing descriptive summaries of individual studies, this synthesis focuses on the essential trade-offs, emerging research trends, and the practical applicability of each method.

**Table 1:** Comparative Synthesis of Modern Fraud Detection Paradigms.

Methodology Paradigm	Key Algorithms	Primary Strengths	Critical Limitations	Current Research Trends
Cost-Sensitive Learning	Example-dependent weighting, Cost-sensitive Ensembles	Directly optimizes financial loss; mitigates class imbalance without synthetic data noise.	Sensitive to cost-matrix design; prone to high false-positive rates if miscalibrated.	Dynamic and adaptive cost-matrix formulation; integration with gradient boosting.
Deep Sequential Models	LSTMs, Attention Mechanisms, Transformers	Captures temporal spending behavior; Transformers enable parallel sequence processing.	High computational overhead; LSTMs suffer from sequential bottlenecks; requires massive data.	Shift from RNNs to Tabular Transformers; self-attention for feature interaction.
Graph-Based Detection	GNNs, HGNNs, Graph Attention Networks (GAT)	Uncovers hidden topological relationships and coordinated fraud rings.	High latency in real-time graph construction; oversmoothing in dense transaction networks.	Heterogeneous graphs; spatial-temporal GNNs; adaptive neighborhood sampling.
Unsupervised Anomaly Detection	Autoencoders (AE), Variational Autoencoders (VAE)	Detects novel, zero-day fraud patterns; requires no labeled data.	High false-positive rates for rare but legitimate transactions; difficult to interpret.	Hybrid models (VAE + GNN); attention-based VAEs; generative adversarial networks (GANs).
Explainable & Secure AI	SHAP, LIME, Federated Learning	Ensures regulatory compliance; preserves cross-institutional data privacy.	Substantial computational and communication latency; limits model complexity.	Lightweight XAI; secure multi-party computation; real-time explainability.

This research tackles the pressing “black box” issue that comes with using complex machine learning models, like deep neural networks or sophisticated ensembles. While these models can achieve impressive accuracy, they often lack transparency in their decision-making processes, leaving stakeholders puzzled about why certain transactions get flagged. This has led to a growing interest in Explainable Artificial Intelligence (XAI). However, much of the existing literature tends to view predictive accuracy and explainability as opposing goals, or it focuses on applying XAI to just one model within a single dataset, which limits how broadly the findings can be applied.

To fill this gap, our study presents a thorough, multi-dimensional framework that connects high predictive performance with strong interpretability across different data environments. Here are the key technical innovations and main contributions of this research:

1. Comprehensive Multi-Dataset Generalizability Framework: Unlike many studies that depend on a single dataset, we rigorously assess four distinct supervised models (Logistic Regression, Random Forest, XGBoost, and LightGBM) across three highly varied public credit card transaction datasets. This multi-environment testing sets a solid foundation for model consistency, adaptability, and real-world applicability.
2. Cross-Model Explainability Comparison: We push the boundaries of XAI by performing a unique, side-by-side comparative analysis using the SHAP (SHapley Additive exPlanations) framework across several high-performing models. This method sheds light on how different algorithmic structures (like gradient boosting versus bagging) prioritize features and interpret the same fraudulent patterns in distinct ways.
3. Actionable Precision-Recall Optimization for Financial Deployment: We dive deep into the precision-recall trade-off tailored for imbalanced fraud datasets. By linking these metrics with XAI outputs, we provide practical, data-driven insights for financial institutions to choose models that align with their specific risk tolerance and operational needs.
4. Real-World Deployment Framework: To bridge the gap between theory and practice, we’re putting forward a detailed deployment framework. This framework tackles essential operational hurdles like inference latency, ensuring system scalability through microservice architectures, keeping an eye on model performance to catch concept drift, and adhering to regulatory standards (think GDPR Article 22 and PSD2). All of this is aimed at making sure our models are fully prepared for real-world production settings^15,16^.

This integrated approach not only enhances the current research landscape but also offers a practical guide for building efficient, transparent, and accountable financial security systems.

Here’s how the manuscript is structured: Section “Related work” covers related works, the section “Methodology” outlines the methodology and datasets, the section “Experimental results” presents the results and XAI analysis, and finally, the section “Conclusions, limitations, and future work” wraps up with the conclusion.

### Benchmarking against state-of-the-art methods

To give a thorough quantitative assessment of the latest developments in the field, we evaluated how well cutting-edge machine learning and deep learning techniques perform in detecting credit card fraud. In Table 2, you’ll find a summary of the performance metrics—Accuracy, Precision, Recall, F1-Score, and AUC—gleaned from recent benchmark studies. These studies made use of a variety of datasets, including the well-known European credit card fraud dataset and other real-world banking datasets. They employed a range of techniques, from traditional ensemble methods like Random Forest and XGBoost to more sophisticated deep learning architectures such as CNNs and Autoencoders, as well as hybrid models. This benchmarking offers a clear view of the current state-of-the-art and underscores the trade-offs between different evaluation metrics, especially when dealing with highly imbalanced datasets.

**Table 2:** Quantitative benchmarking of state-of-the-art methods in credit card fraud detection.

Reference	Year	Best Model	Accuracy	Precision	Recall	AUC/F1
Alarfaj et al.^30^	2022	CNN (Deep Learning)	99.90%	93.00%	85.71%	AUC: 98.00%
Afriyie et al.^7^	2023	Random Forest	96.00%	-	-	AUC: 98.90%
Khalid et al.^31^	2024	Ensemble (SVM + KNN+RF+Bagging+Boosting)	99.95%	99.95%	99.95%	F1: 99.95%
Hossain et al.^32^	2024	Gradient Boosting	99.96%	98.50%	98.00%	AUC: 97.00%
Shah & Sharma^33^	2025	XGBoost	97.12%	84.44%	73.57%	AUC: 98.77%
Our Study (Dataset 2)	2026	XGBoost	99.71%	85.82%	59.89%	AUC: 99.62%

## Methodology

While the foundational algorithms used in this study are well-known, the real innovation comes from our extensive experimental and framework contributions. Instead of rolling out a brand-new algorithm, this methodology presents a solid, multi-dimensional framework that effectively connects predictive accuracy with model transparency. Our experimental contribution shines through with thorough cross-dataset validation of these models, even in cases of extreme class imbalance, along with a distinctive side-by-side comparative Explainable AI (XAI) framework. This strategy offers financial institutions a systematic way to not only achieve impressive fraud detection rates but also to quantitatively and qualitatively assess how various algorithmic architectures make their decisions.

The approach used in this research is based on supervised machine learning due to its success in detecting well-established patterns of fraud given past activity. The existence of tagged data sets with transactions already labeled as either fraudulent or genuine makes supervised machine learning a viable method for this type of classification problem. The goal for this research is to create models that are able to identify the characteristics of fraudulent behavior and then use this information to classify new transactions.

For this comparative study, four different supervised models were chosen: Logistic Regression, Random Forest, XGBoost, and LightGBM. The choice was deliberate, representing different levels of complexity and capability. Logistic Regression is great for identifying simple linear relationships, while models like Random Forest, XGBoost, and LightGBM are built to handle more complex, non-linear interactions. They excel at understanding feature dependencies and the ensemble decision-making processes that often characterize real fraud behavior.The choice of Logistic Regression as the baseline model was due to its simplicity, interpretability, and efficiency. This makes it easy to compare its performance with complex models. The other three models: Random Forest, XGBoost, and LightGBM, represent different types of tree-based ensemble models. They were chosen for their high level of performance proven in numerous machine learning competitions, including fraud detection. The ensemble models have been noted for their ability to combine the forecasted values from different models (decision trees in this case), which makes them robust, able to deal with complex non-linear relationships between features in the datasets, and highly accurate.

While deep learning models like CNNs, RNNs, LSTMs, and Transformer-based architectures are crucial in the realm of fraud detection, as highlighted in the section “Deep learning architectures: LSTMs and transformers” and compared with previous studies in Table 2, they weren’t part of the main experimental comparison for a few key reasons. First off, this study aimed to conduct a controlled, cross-dataset evaluation of interpretable supervised models using publicly available tabular transaction datasets, where tree-based ensembles have proven to be strong and reliable baselines. Secondly, the three datasets utilized here are mainly tabular and often lack the long, well-structured cardholder transaction sequences that are essential for fully leveraging the capabilities of sequential or self-attention-based models like LSTMs and Transformers. Moreover, Transformer-based architectures typically demand much larger training datasets, greater computational power, and more extensive tuning to ensure stable and fair cross-dataset comparisons, which would have diverted the focus of this study from its primary goal of balancing predictive performance with model explainability. Similarly, CNN-based methods often rely on task-specific feature representations, which can hinder direct comparisons across different datasets. Ultimately, this study emphasizes explainability, reproducibility, and practical deployment. In contrast to deep neural architectures, models such as Logistic Regression, Random Forest, XGBoost, and LightGBM are more computationally efficient, train more reliably under significant class imbalances, and offer more consistent SHAP-based interpretations across datasets. For these reasons, deep learning models, including Transformers, are regarded in this manuscript as valuable state-of-the-art references and promising avenues for future research, rather than as integral parts of the main comparative framework.

**Fig. 1:**
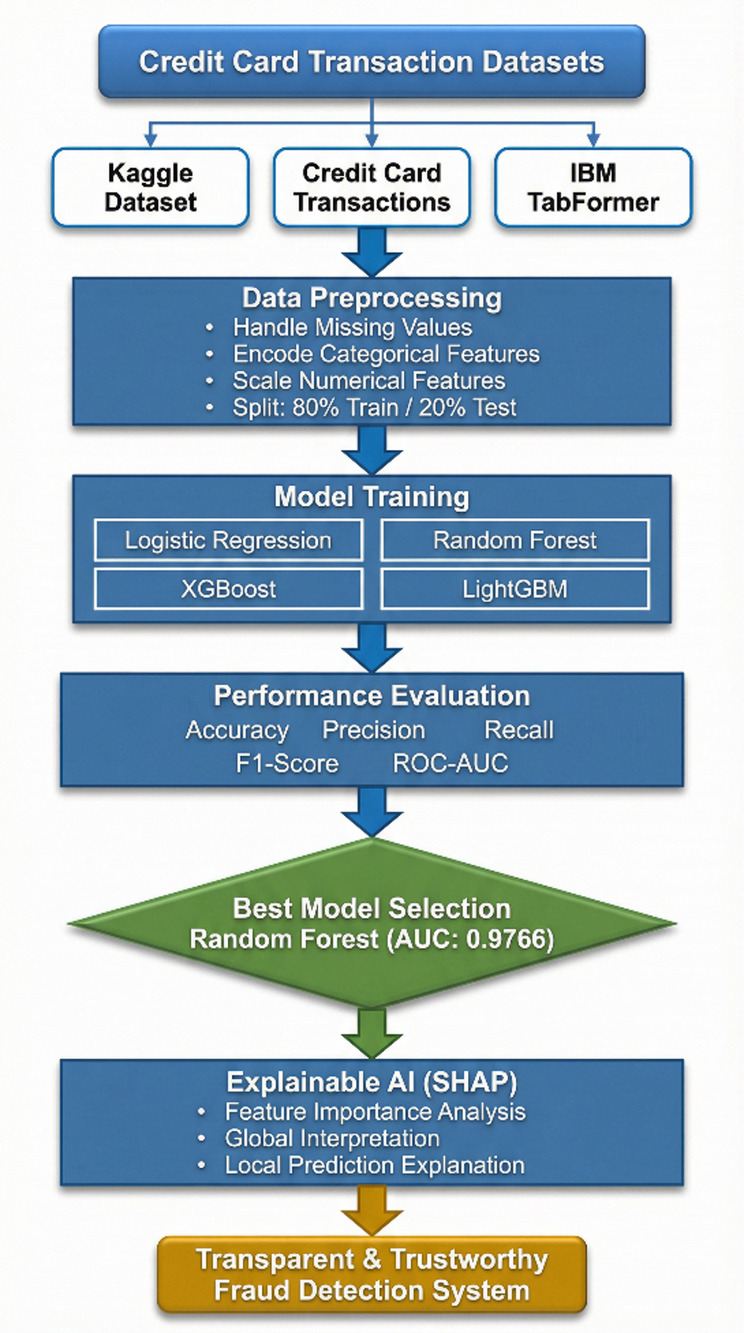
Supervised Fraud Detection Methodology with Explainable AI Framework.

### Data preparation and preprocessing pipeline

This project dives into a comprehensive data preparation pipeline designed for building models. It leverages three publicly available datasets related to credit card transactions to provide a well-rounded evaluation. Here’s a breakdown of the pipeline steps:

- Data Selection: The three datasets utilized include the Credit Card Fraud Detection dataset from Kaggle, an enhanced Credit Card Transactions dataset, and the IBM TabFormer dataset.
- Preprocessing Pipeline: We systematically apply standard preprocessing techniques. This involves filling in missing values—using the median for numerical features and the mode for categorical ones. Categorical variables are transformed into numeric ones through One-Hot Encoding. To ensure that no single variable overshadows the model, we scale numerical features with RobustScaler, which is more resilient to outliers, a common issue in fraud detection data.
- When it comes to Data Splitting and Cross-Validation, we want to make sure our models can really perform well and aren’t just memorizing the data from a specific train-test split. That’s why we use a 5-Fold Stratified Cross-Validation strategy. This approach helps us keep the extreme class imbalance—where fraudulent transactions are much fewer than normal ones—consistent across all the training and validation folds.
- Now, about Handling Class Imbalance: In the world of fraud detection, class imbalance can lead to skewed models. To tackle this, we turned to the Synthetic Minority Over-sampling Technique, or SMOTE for short. We set SMOTE up with k_neighbors = 5 and made sure to apply it only to the training folds during cross-validation. This way, we avoid any data leakage and can create synthetic samples of the minority class until we reach a more balanced distribution.

In our methodology, we chose SMOTE as our resampling technique because of the significant imbalance ratio of just 0.172% in the datasets. We decided against random undersampling since it tends to throw away a lot of valid transaction data, which could lead to our models missing out on important patterns of normal behavior. While ADASYN aims to create synthetic samples close to the decision boundary, it can end up adding too much noise in heavily skewed financial datasets by generating too many minority samples in outlier areas. Additionally, although Cost-Sensitive Learning (CSL) is a good alternative to resampling, we preferred to tackle cost-sensitivity separately during the evaluation phase (see the section “Cost-sensitive evaluation”). This approach allowed our ensemble models to train on a more balanced representation before we applied any threshold and cost optimizations.

### Model training, hyperparameter tuning, and evaluation

This approach outlines how we train and test supervised machine learning models, emphasizing the importance of thorough hyperparameter optimization to find the best algorithm for detecting fraud.

- Algorithm Selection and Hyperparameters: We compare four different supervised classifiers, from straightforward linear models to more intricate ensemble methods. To ensure we get the best performance, we used RandomizedSearchCV for hyperparameter tuning, running 50 iterations and focusing on optimizing the ROC-AUC score.
- Logistic Regression: This serves as our baseline model. The key parameters we fine-tuned include C = 1.0, penalty=’l2’, solver=’lbfgs’, and max_iter = 1000 to guarantee that the model converges effectively.
- Random Forest: This is a bagging ensemble model. The best setup we found includes n_estimators = 200, max_depth=None, min_samples_split = 5, and class_weight=’balanced’ to effectively address errors in the minority class.
- XGBoost: An advanced framework for gradient boosting. After tuning, we settled on learning_rate = 0.1, max_depth = 6, n_estimators = 200, and scale_pos_weight adjusted to reflect the ratio of negative to positive instances, which helps manage imbalance internally.
- LightGBM: A super-efficient gradient boosting model. The fine-tuned hyperparameters are learning_rate = 0.05, num_leaves = 31, n_estimators = 200, and is_unbalance=True.

To promote transparency and reproducibility, we defined the hyperparameter search space for the RandomizedSearchCV process as follows: For Logistic Regression, the regularization parameter ‘C’ varied across [0.01, 0.1, 1, 10, 100]. In the case of Random Forest, ‘n_estimators’ ranged from [100, 200, 300, 500], ‘max_depth’ included [None, 10, 20, 30], and ‘min_samples_split’ was set between^7,29,34^. For XGBoost, we explored ‘learning_rate’ values of [0.01, 0.05, 0.1, 0.2], ‘max_depth’ options of^35–37^, and ‘n_estimators’ choices of [100, 200, 300]. Lastly, for LightGBM, ‘learning_rate’ spanned [0.01, 0.05, 0.1], ‘num_leaves’ included [31, 50, 100], and ‘n_estimators’ was also [100, 200, 300]. We conducted fifty iterations within these ranges to pinpoint the best configurations for each model.

-When we dive into the performance of our trained models, we look at several key metrics: Accuracy, Precision, Recall, F1-Score, ROC-AUC, and, in cases of severely imbalanced datasets, we also consider Precision-Recall AUC (AUC-PR). Since fraud detection datasets tend to be quite skewed, AUC-PR gives us valuable insights into how well we’re doing with the minority fraud class. Meanwhile, we keep ROC-AUC in the mix for a threshold-independent comparison across different models.

### Model interpretation using explainable AI (XAI)

Such a methodology will help counter the transparency problem of complex models by using a framework of Explainable AI (XAI).

- XAI Framework: The SHAP (SHapley Additive exPlanations) framework is applied. SHAP is a game theory-based methodology that explains a result of any machine learning model by giving every input (feature) an importance value (SHAP value) for every prediction made.
- Interpretation of Results: SHAP is used on the best-performing models (in this case, Random Forest and XGBoost) to provide insights into how these models work. This results in:
- Global Interpretation: What are the key features that drive a transaction into being fraudulent versus legitimate within the entire data set that this model uses?
- Local Interpretation: local explanation for individual predictions, showing what made a particular transaction be labeled as fraudulent.
- Establishing Trust: The offering of this transparency also boosts trust in the AI system as it plays a crucial role within the financial industry where accountability and explainability are of utmost importance.

To enhance explainability, this study employs SHAP at both global and local levels. On a global scale, SHAP summary and feature-importance analyses pinpoint the variables that consistently influence fraud predictions across various datasets. At the local level, SHAP force and dependence plots clarify why a particular transaction gets flagged, allowing analysts to connect each model decision to understandable feature contributions. Additionally, the manuscript quantitatively assesses explanation stability across models by using rank correlation, overlap, and consistency measures, demonstrating that explainability is regarded as a key evaluation dimension, not just a visual tool.

## Experimental results

This section describes the experimental results that were derived from developing fraud detection models in this study. The organization of this section includes: The section “Environment”, which describes the experimental environment; the section “Datasets and training”, which gives details about the datasets that were utilized in both training and testing; the section “Machine learning”, which describes the results derived from developing supervised machine learning models; the section “Explainable AI”, which describes how fraud detection models were analyzed using techniques from Explainable AI (XAI); and finally, the section “Discussion” discusses the overall findings and their implications.

### Environment

The experiments were carried out on a setup with the following specifications: a personal computer with an Intel Core i5 processor and a RAM of 6 GB. For the need to leverage additional computing power, the experimental design also utilized cloud-based services, specifically Google Colaboratory, otherwise referred to as Colab, as well as Kaggle. The platforms offer virtual machines that are already pre-equipped with scientific computing packages, as well as the capacity to leverage GPU or TPU processing, hence facilitating rapid computing suitable for the application of machine learning. The experimental setup, therefore, utilized both a personal computer as well as cloud-based platforms.

### Datasets and training

There are three distinct data sets used as training and testing data for the fraud detection models. The data sets selected include a variety of transactions and fraud. The publicly available data sources used in this project are as follows:

1. Credit Card Fraud Detection^38^: This dataset contains 284,807 transactions, including 492 fraudulent cases. The problem involves European credit card transactions performed in September 2013. It is heavily imbalanced as only 0.172% of transactions were fraudulent. Variables are anonymized for data protection and consist of 28 significant components from PCA transformation (V1 to V28), transaction amount, and a response variable (0 for legitimate and 1 for fraudulent).
2. Credit Card Transactions Dataset^39^: This dataset contains over 1.85 million transaction records. It consists of a much larger pool of transactions, along with more informative attributes like the transaction value, geographic information, merchant information, and customer data.
3. IBM TabFormer Dataset^40^: This dataset contains 24 million synthetic credit card transaction records. IBM has launched the IBM TabFormer Dataset, a data collection of different characteristics of credit card transactions.

For each of the datasets, certain preprocessing techniques were performed, such as dealing with missing values, conversion of non-numeric attributes to numeric, and scaling of attributes. The datasets were divided into training and testing subsets, consisting of 80% and 20%, respectively, while ensuring stratification to maintain equal proportions of fraud and normal transactions in each subset.

### Machine learning

This report includes the results of supervised machine learning algorithms used in fraud detection.

#### Supervised learning results

Results of The results of the above-mentioned supervised machine learning models on the Kaggle CreditCardFraud dataset^38^, the Priyam Choksi Credit Card Transactions dataset^39^, and the IBM TabFormer dataset^40^. The four models are Logistic Regression (LR), Random Forest (RF), XGBoost (XGB), and LightGBM (LGBM). For the results mentioned above, standard preprocessing is done, and the splitting is 80 − 20 Stratified Split. The result measures are accuracy, precision, recall, F1-Score, and ROC AUC value.

##### Dataset 1: Kaggle (credit card fraud detection)

The Kaggle Credit Card Fraud Detection dataset^38^, a widely used source, was used in the research. PCA was used as a method of feature transformation in the data, excluding the features of “Time” and “Amount”. The data contains a level of imbalance, as the minority class represents just 0.172% of the data.

- Overall Model Performance (Table 3)

**Table 3:** The overall performance of the models was evaluated using Accuracy and Area Under the Receiver Operating Characteristic Curve (AUC).

Model	Accuracy	AUC
Logistic Regression	0.975457	0.972063
Random Forest	0.999386	0.976574
XGBoost	0.999315	0.974668
LightGBM	0.999368	0.959964

Within the tree-based ensemble models, Random Forest, XGBoost and LightGBM reached resounding accuracies (close to 99.9%) compared to Logistic Regression reaching only a mere 97.5% accuracy. This exceptional performance is due to the way that ensemble models have a unique ability to capture complex non-linear interactions between features — an attribute that linear models such as Logistic Regression lack. But a high accurate score can be deceptive when dealing with highly imbalanced fraud datasets. The Area Under the ROC Curve (AUC) provides a more reliable score for that reason. Random Forest had the highest AUC of 0.9766 among these models, which can be attributed to its ability through the bagging technique to decrease variance and prevent overfitting on a majority class leading to superior differentiation between legitimate and fraudulent transactions.

- Class-specific performance

To get an insight into the performance characteristics of these models, individual class performances were analyzed.

**Class 0: Normal transactions** (Table 4)

**Table 4:** the Precision, Recall, and F1-Score for the normal transactions class (Class 0).

Model	Precision_Class0	Recall_Class0	F1_Class0
Logistic Regression	0.999856	0.975556	0.987556
Random Forest	0.999701	0.999683	0.999692
XGBoost	0.999719	0.999596	0.999657
LightGBM	0.999701	0.999666	0.999683

The results for all the models for identifying legitimate transactions (Class 0) were excellent, with precision and recall near 1.0. This is because the training data will be skewed due to a greater proportion of examples from the majority class, allowing these algorithms to develop an understanding for what a normal behavior looks like. This greatly lowers the likelihood of legit transactions getting falsely flagged.

**Class 1: Fraudulent transactions** (Table 5)

**Table 5:** The performance on the fraudulent transactions class (Class 1) is of paramount importance in this study.

Model	Precision_Class1	Recall_Class1	F1_Class1
Logistic Regression	0.060811	0.918367	0.114068
Random Forest	0.818182	0.826531	0.822335
XGBoost	0.780952	0.836735	0.807882
LightGBM	0.810000	0.826531	0.818182

As compared to the result obtained in the performance for Class 0, there is a greater variability in the results obtained for Class 1. Although the recall achieved by the Logistic Regression classifier is high, the precision is found to be quite low, at a mere 0.0608. It is a warning flag indicating that there are a lot of false positives. Random Forest, XGBoost, and LightGBM were more balanced in terms of precision and recall. Random Forest had the best precision of 0.8182 and the best F1-score of 0.8223, implying it is the most balanced algorithm in detecting fraud transactions. However, XGBoost performed slightly better in terms of recall of 0.8367 than Random Forest, although at the cost of lower precision. LightGBM performed equally well in terms of precision and F1-score, similar to Random Forest.

- Average model performance

**Table 6:** The macro average metrics provide a single measure for the models’ performance across both classes.

Model	Precision_Macro	Recall_Macro	F1_Macro
Logistic Regression	0.530333	0.946962	0.550812
Random Forest	0.908941	0.913107	0.911014
XGBoost	0.890335	0.918165	0.903769
LightGBM	0.904851	0.913098	0.908933

**Table 7:** weighted average metrics provide a single measure for the models’ performance across both classes.

Model	Precision_Weighted	Recall_Weighted	F1_Weighted
Logistic Regression	0.998240	0.975457	0.986054
Random Forest	0.999389	0.999386	0.999387
XGBoost	0.999342	0.999315	0.999327
LightGBM	0.999375	0.999368	0.999371

In Table 6, the macro-averaged metrics, which handle the two classes equally, clearly highlight the effectiveness of Random Forest, XGBoost, and LightGBM models over Logistic Regression. In Table 7, as the class imbalance present in the dataset affects the weighted-average metrics, they largely resemble the overall accuracy.

- Confusion matrix analysis

**Table 8:** The confusion matrix provides a detailed breakdown of the models’ predictions.

Model	True_Negative	False_Positive	False_Negative	True_Positive
Logistic Regression	55,474	1390	8	90
Random Forest	56,846	18	17	81
XGBoost	56,841	23	16	82
LightGBM	56,845	19	17	81

The confusion matrix for the Logistic Regression model is shown in Table 8 which shows a considerable number of false positives (*n* = 1,390), setting aside the precision for the model to classify a record as fraud. The other models, namely Random Forest, XGBoost, and LightGBM, have a lower number of false positives (*n* = 18, 23, and 19, respectively). With reference to the number of false negatives, all models have performed well with the lowest value recorded by the Logistic Regression model (*n* = 8). However, the model is not applicable due to the considerable number of false positives.

On the basis of the overall assessment, the following key findings emerge:

- All four models have an Area Under the Curve of over 0.90, suggesting the efficacy of the models in the fraud detection problem.
- Random Forest: This model is identified as the overall best performer as it has the highest AUC of 0.9766, along with the most optimal balance for the fraud class F1-Score of 0.8223.
- Logistic Regression has the highest recall for cases in the fraudulent class (0.9184), but it has a problematic precision, which leads to a lot of false positives.
- There is also strong performance by XGBoost and LightGBM, comparable to that of Random Forest.

In conclusion, it can be recommended that the Random Forest model is the most appropriate solution to this fraud detection problem because it performs best in terms of detecting fraudulent transactions while avoiding false alerts.

**Fig. 2:**
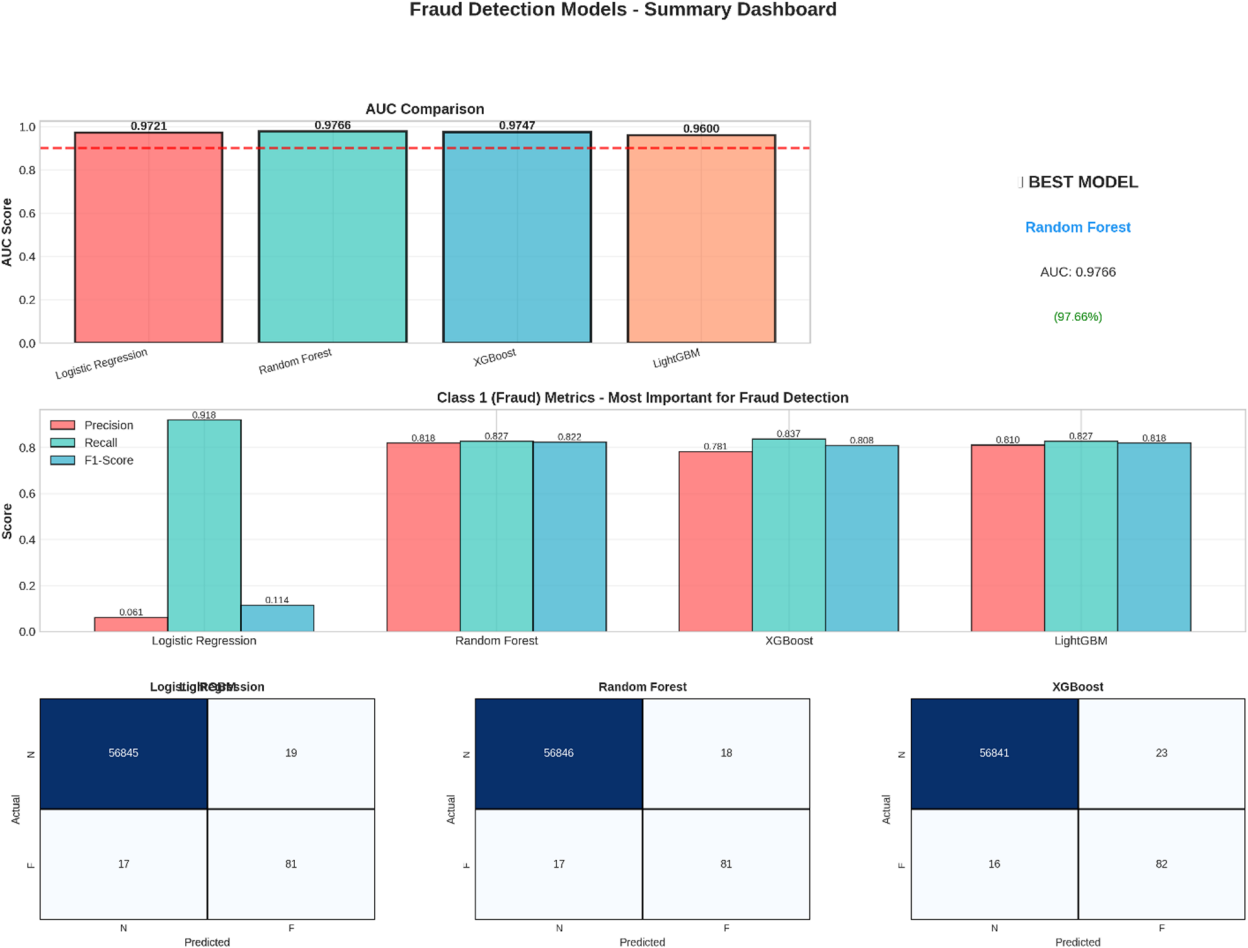
Fraud Detection Models - Summary Dashboard.

A brief summary of the results is offered by “Figure 2,” which is concerned with the performance of the model and the metrics that are the most relevant to the problem of fraud detection. The metrics are presented through a dashboard that.

1. AUC Comparison: In this bar chart, a comparison of the Area Under the Receiver Operating Characteristic Curve (AUC) among all models is made. The highest value of AUC is achieved by the Random Forest model with a value of 0.9766, thus performing best in discriminating between fraudulent and genuine transactions. All models achieve values higher than the required target of 0.90.
2. Class 1 (Fraud) Metrics: This section compares the performance of models on the task of Fraud Detection, which is the primary goal. The metrics compared here are Precision, Recall, and F1-Score on the Fraud class. The performance of the Logistic Regression model is relatively poor on this particular class. However, the other three models have a good trade-off between Precision and Recall.
3. Confusion Matrices: Confusion matrices for the top three models are shown on the dashboard, which gives information on the error made by the models with respect to predictions. Confusion matrices list the actual and predicted figures, which can be analyzed for the implications of the predictions made.

The summary dashboard, on the whole, concludes that the Random Forest algorithm has been the most efficient approach in the given problem of detecting fraudulent activity, as backed by its better AUC and equitable performance on the crucial class of fraud.

**Fig. 3:**
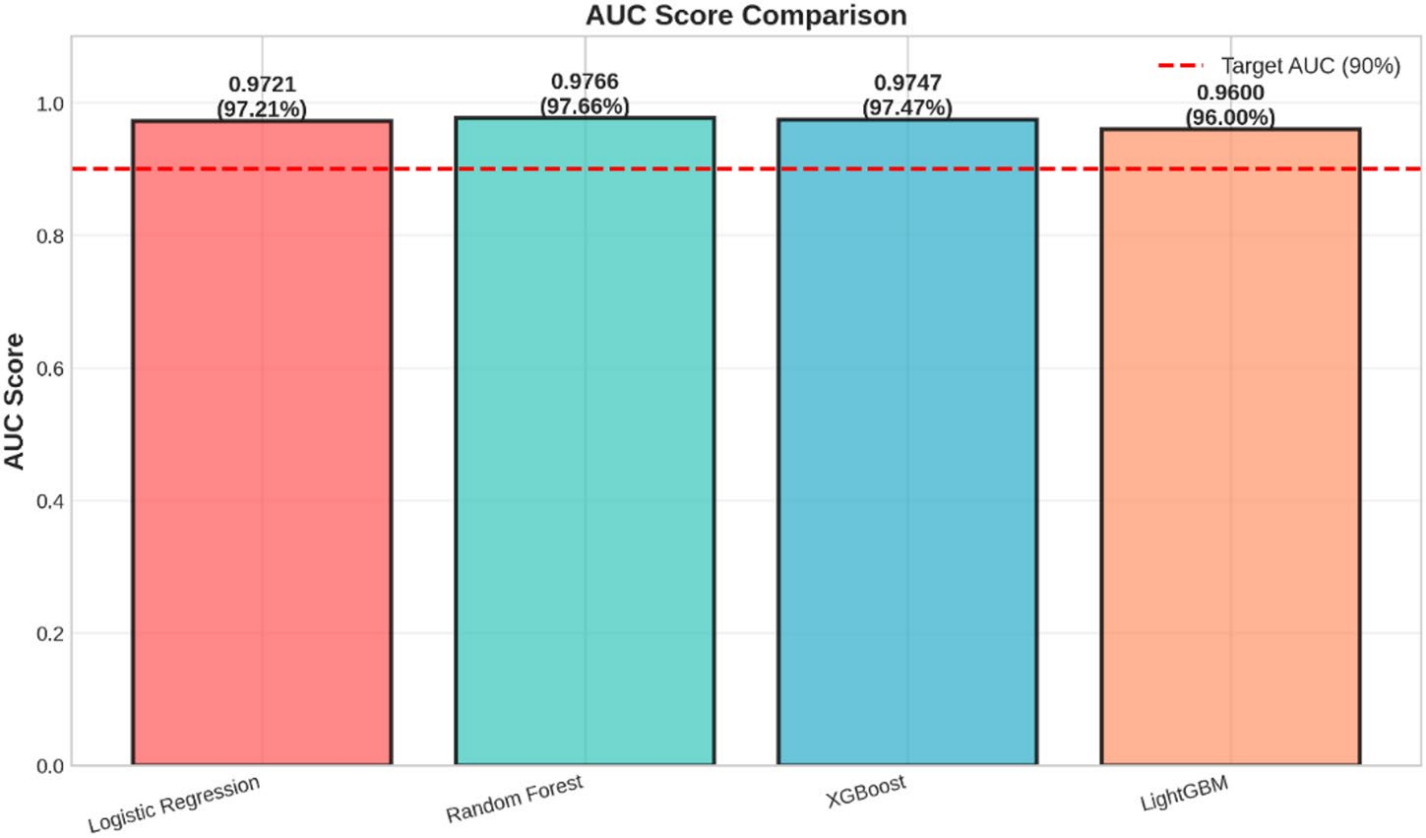
AUC Score Comparison.

The AUC scores of the four models can be directly compared using the bar chart in Fig. 3. The value of AUC represents the discrimination of the model on the classes of interest, with higher values reflecting better performance.

The Random Forest model has the highest AUC value of 0.9766, followed closely by the XGBoost with AUC value of 0.9747, and then the Logistic Regression with an AUC value of 0.9721. The LightGBM also performs quite well with an AUC value of 0.9600. All four models perform greatly beyond the target AUC value of 0.90, as shown by the red dashed line, which indicates an outstanding ability to distinguish between fraud and genuine transactions. The outstanding performance by the Random Forest on this measure further supports its identification as the best-performing model.

**Fig. 4:**
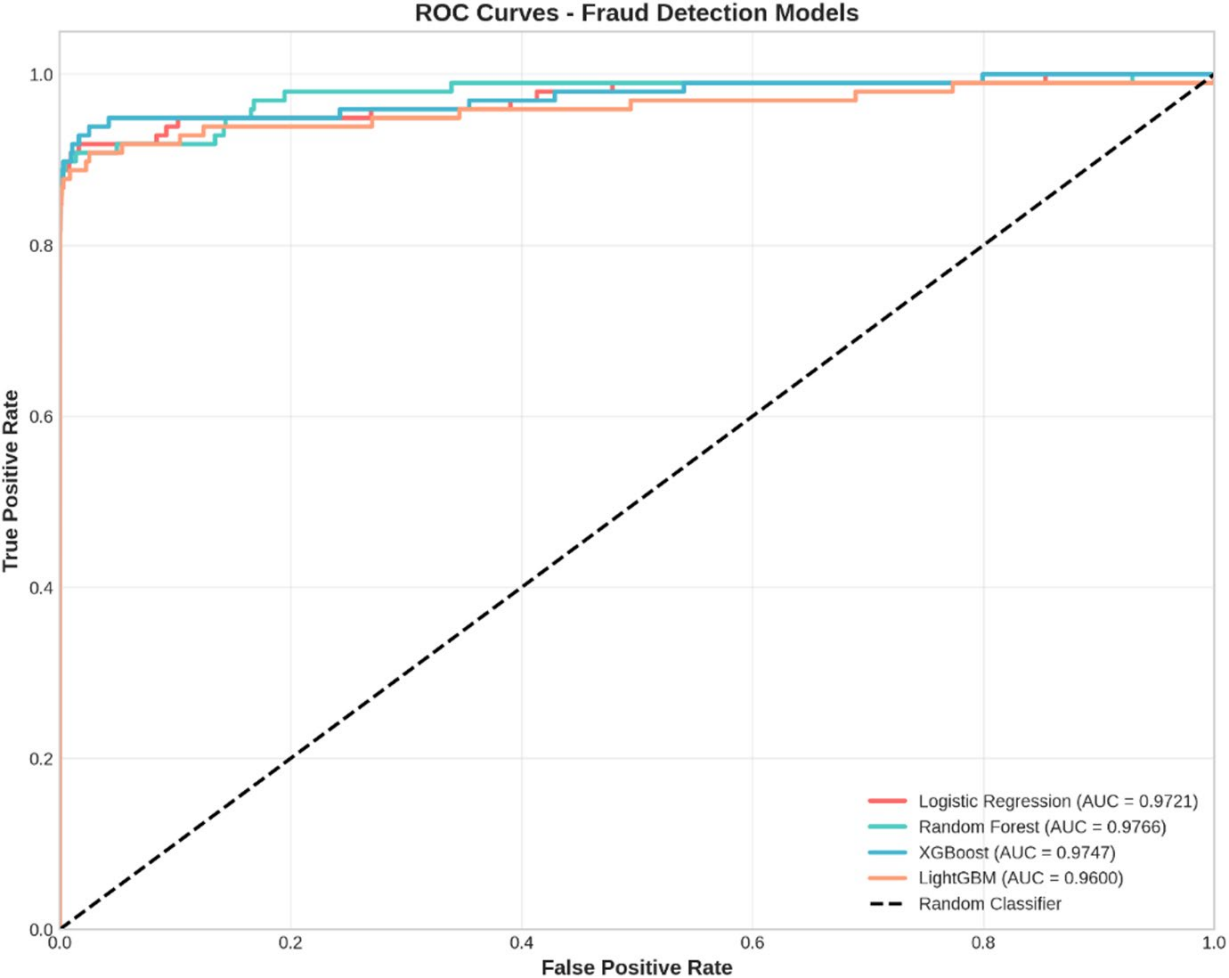
ROC Curves - Fraud Detection Models.

Figure 4 shows the Receiver Operating Characteristic (ROC) curves for all four models. The ROC curve is a graphical representation of a binary classifier’s diagnostic capability at various thresholds, tracing out a curve on a plot of True Positive Rate against False Positive Rate. The best possible ROC curve would lie at the top and left-hand side of this graphical representation, touching the top and left boundary, with a TPR of 100% and an FPR of 0%. The diagonal dashed line in each plot represents the performance of a random model, and all models performing better than a random model lie above it. All four models perform remarkably well, lying above this diagonal line and closer to the top and left boundary of each plot, suggesting a low FPR and high TPR at various thresholds. The area under each curve has been quantified to yield results consistent with Fig. 3.

**Fig. 5:**
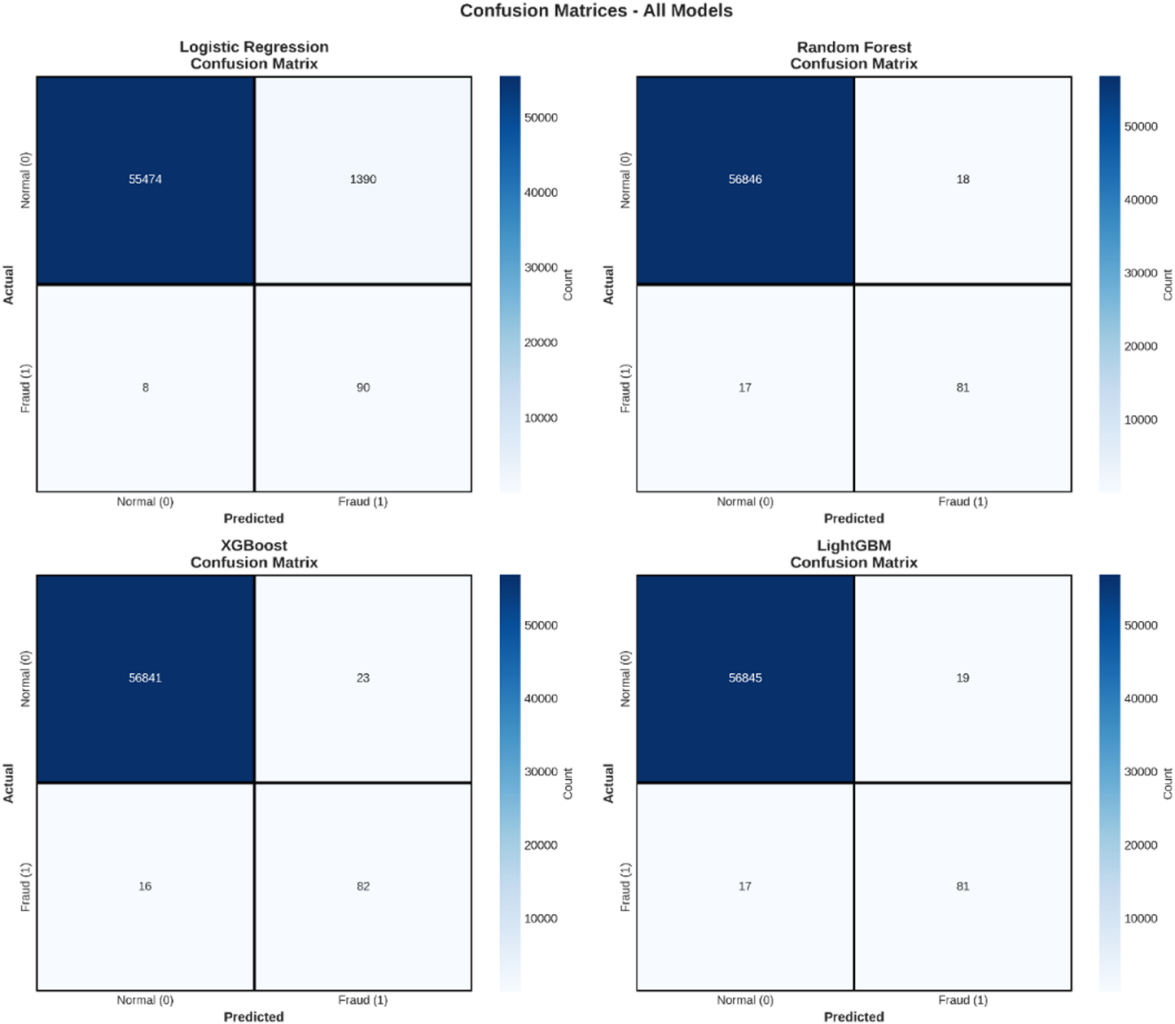
Confusion Matrices - All Models.

Confusion Matrices - All Models

In Fig. 5, the confusion matrices for the four models are presented, which give information on the classification performance. In the confusion matrix, the predictions made by the model with respect to the actual class are divided into four quadrants:

- True Negatives (TN): The top-left corner represents correctly identified normal transactions.
- False Positives (FP): Top-right quadrant, indicating genuine transactions mistakenly labeled as fraud.
- False Negatives (FN): Lower left quadrant, which holds fraud transactions labeled as legitimate; this is also very important in fraud identification since it denotes undetected frauds.
- True Positives (TP): The bottom-right quadrant, signifying the proper detection of fraudulent transactions.

From the confusion matrices, all models perform well on finding normal transactions, as they provide a considerable number of true negatives. However, their performance in finding fraud differs. The Logistic Regression model produces the highest number of false positives (1,390), indicating it marks many legitimate transactions as fraud. Although this is the highest number of false positives, it is the lowest number of false negatives (8), indicating that it misses the fewest number of actual fraud transactions. The other three models (Random Forest, XGBoost, and LightGBM) perform better on handling false positives (18, 23, and 19, respectively), indicating they are more accurate. However, they produce a slightly higher number of false negatives (17, 16, and 17, respectively), indicating they miss a few more actual fraud transactions compared to Logistic Regression.

**Fig. 6:**
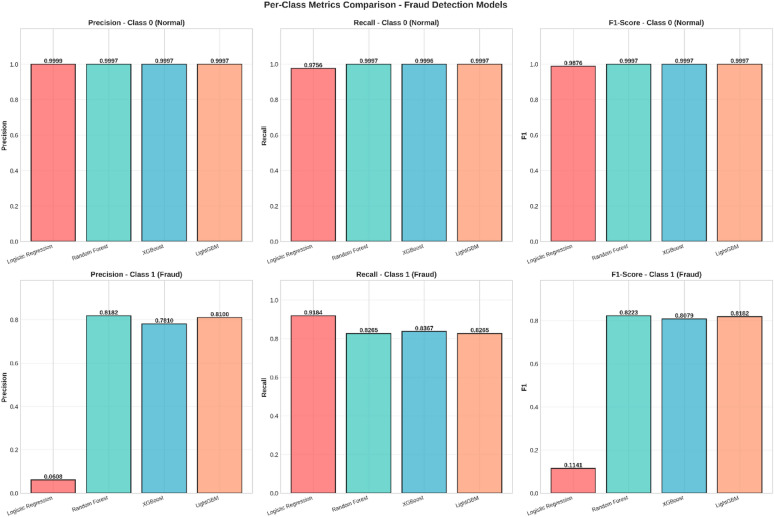
Per-Class Metrics Comparison - Fraud Detection Models.

Figure 6 The performance of the model on the data, broken down by class: Normal versus Fraud, or Class 0 versus Class 1.

- Class 0 (Normal): The top row represents all four models performing exceptionally well in terms of Precision, Recall, and F1 Score on normal transactions, which clearly represents a high capability to detect normal activity with a low probability of false positives.
- Class 1 (Fraud): The last row denotes the challenge of fraud identification. There is considerable variation in models for fraud classes. The model has poor performance for Logistic Regression with very low Precision (0.0608) and F1-score (0.1141), which indicates numerous instances of fraud classified as others for increased Detection Rates since it correctly identifies cases of fraud.

The models of Random Forest, XGBoost, and LightGBM model perform better by weighing Precision and Recall for better F1-score and hence pinpointing fraud instances while minimizing errors.

**Fig. 7:**
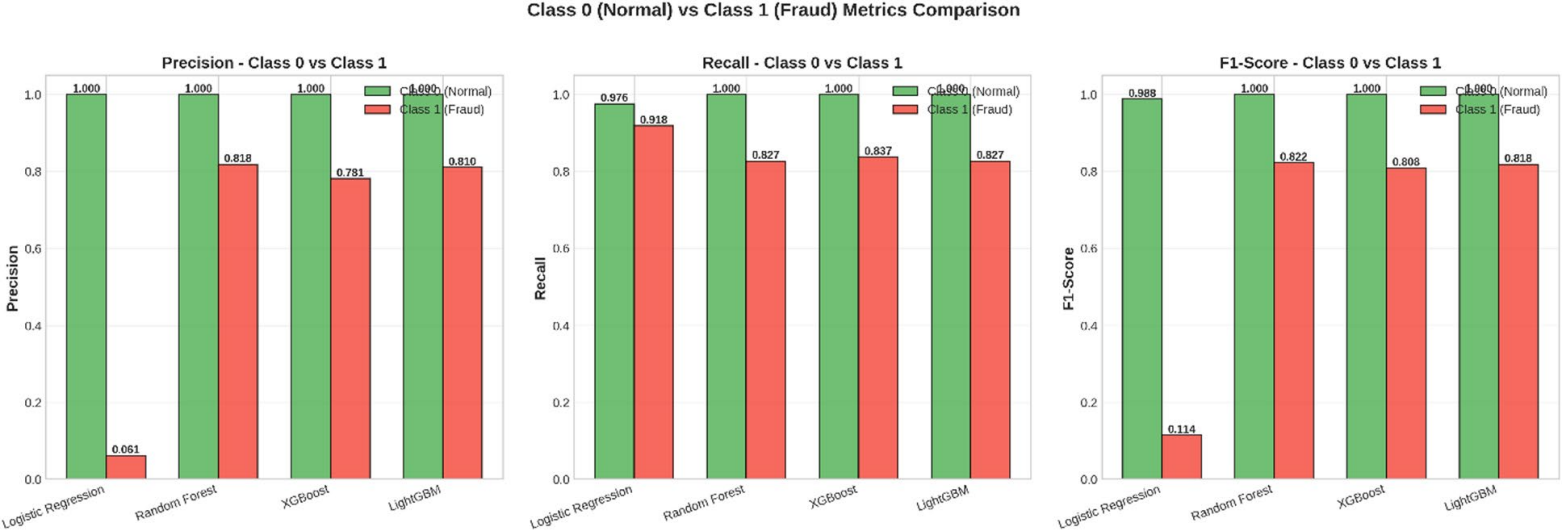
Class 0 (Normal) vs. Class 1 (Fraud) Metrics Comparison.

In Fig. 7, a comparative analysis is done regarding the performance parameters – Precision, Recall, and F1 Score – between Class 0 and Class 1. In each performance metric, Class 0 performs optimally with a score close to 1.0, establishing the efficiency of models in picking out genuine transactions. In each case, Class 1 performance shows a large amount of variation. In Class 1, the performance of the Logistic Regression model is found to be considerably low – with a Precision value of 0.061 and F1 Score of 0.114 – implying that only a small proportion of its positive predictions are correct. But tree-based models – Random Forest, XG Boost, and LightGBM – perform much better compared to other models with respect to fraudulent transactions. Through Fig. 7, it is revealed that tree-based models are more efficient regarding fraudulent transactions.

**Fig. 8:**
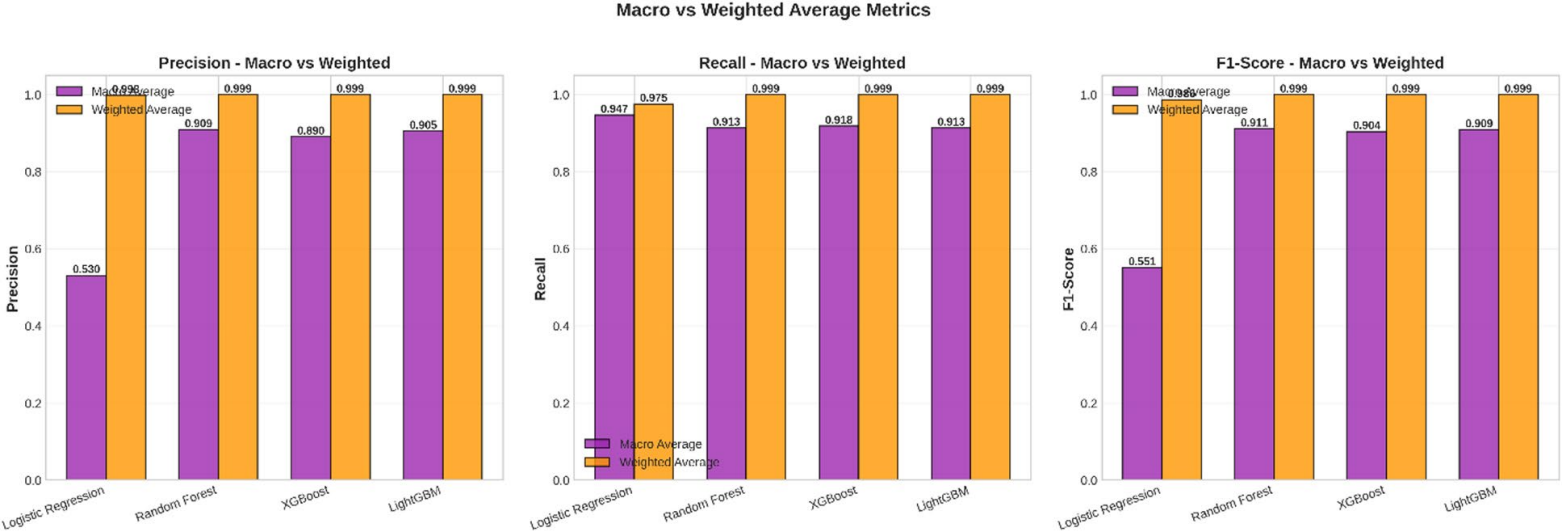
Macro vs. Weighted Average Metrics.

In Fig. 8, the Macro Average and Weighted Average of the Precision, Recall, and F1 Score of all four models are compared. It is crucial to comprehend the difference between these two approaches in the context of the imbalanced datasets.

- Weighted Average: This measure calculates the average value of the measure for each class, weighted by the number of occurrences in the class. Since the Normal class (Class 0) is orders of magnitude larger than the Fraud class (Class 1), the weighted average is highly dominated by the Normal class. Therefore, the weighted average of all the models looks extremely high, which is not representative of the model’s efficacy to identify frauds.
- Macro Average: The macro average calculates the average value of the measure for all classes with equal weights, irrespective of the class sizes. The result of the macro average provides a more level representation of the performance of the model on all classes. The relatively lower scores of the macro averages, especially for Logistic Regression, represent the performance on the fraud class. The following figure explains the need for the use of relevant assessment metrics in the case of imbalanced classification problems. Although the weighted average would seem close to perfect in the case of all models, the actual shortcomings in the minority class detection capability of certain models become evident in the macro-average. When dealing with highly imbalanced fraud datasets, it’s important to consider AUC-PR as well. This metric really hones in on the precision-recall trade-off for the minority class, which is something that ROC-based evaluations might overlook.

**Fig. 9:**
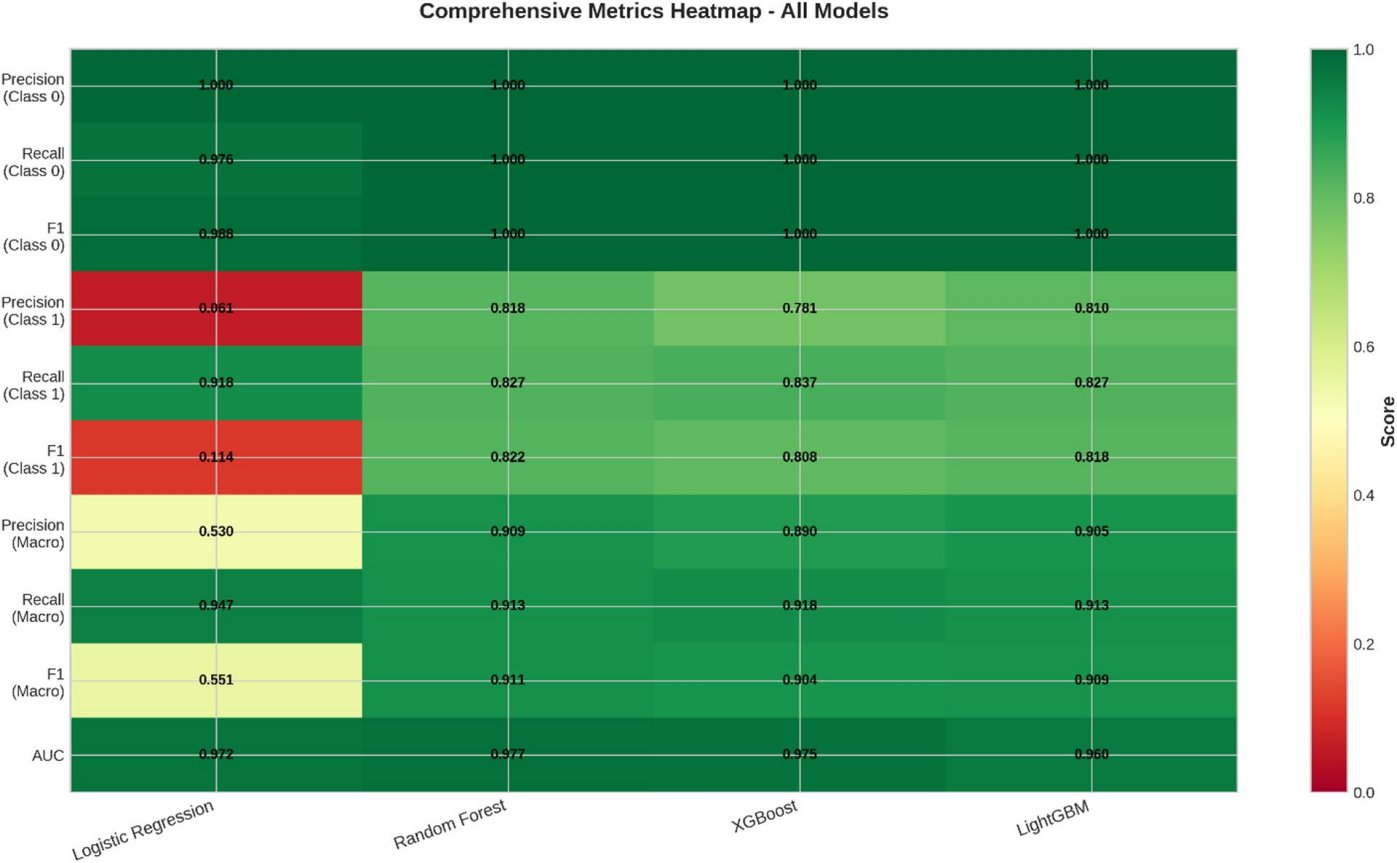
Comprehensive Metrics Heatmap for All Models.

The Fig. 9 integrates all evaluation metrics for all four models into one visualization using color-coding. The heatmap makes it convenient to compare model results on all metrics side by side.

The color range varies from red, indicating poorer performance, to green, indicating better performance. All models clearly have outstanding performance on the parameters related to the majority class, which is Class 0, as is visible from the dark green coloring of the cells for Precision, Recall, and F1-Score for Class 0.

Variations appear to be significant for the most appropriate metrics of the minority class (Class 1) and macro-averaged metrics. In this case, the Logistic Regression model has reddish to yellowish tones for the said metrics, indicating a lower level of performance for fraud detection. On the other hand, an indication of superior and equally well-balanced performance for all models is shown by their greener profiles. Overall, this matrix succinctly shows how each model fares and contributes to identifying tree-based models as being appropriate for this fraud detection problem.

##### Dataset 2: Credit card transactions (Kaggle)

The dataset used is Credit Card Transactions dataset^39^, which has geographical and temporal features such as amt, lat, long, city_pop, unix_time. The dataset is class imbalanced because the fraud class or class “1” represents about 0.17% of the class after pre-processing. Evaluation criteria include Accuracy, Area under the Receiver Operating Characteristic Curve “AUC-ROC,” (AUC-ROC), Area under the Precision-Recall Curve (AUC-PR), as well as per-class performance “Precision,” “Recall,” “F1-Score.”

- Overall model performance

The performance metrics of the aggregate model are presented in Table 9. All the models achieved an accuracy of greater than 99%, primarily due to the large prevalence of the non-fraud class. But when we examine the true classification capabilities of both methods, we notice it on our AUC-ROC metric. Among them, XGBoost and Random Forest had the best AUC-ROC values (0.9962 and 0.9863, respectively). The exceptional capabilities of XGBoost are attributed to the gradient boosting framework, which iteratively minimizes the error residuals from prior trees. It is especially adept at detecting the subtle, rare patterns typical of fraudulent transactions because of this.

Speaking of which, XGBoost absolutely crushed both Logistic Regression and Random Forest on Dataset 2. Its specific way of gradient-boosting trees in a sequence enables it to learn from the errors made by trees preceding it which plays a huge role in its efficacy as compared to the bagging process employed by Random Forest or Logistic Regression’s linear decision boundary when detecting those subtle, non-linear patterns of fraud. This is particularly important when dealing with highly imbalanced fraud data, where those rare fraudulent transactions can easily be drowned out by the legitimate ones. As they say, the proof of the pudding is in the eating: while XGBoost shows overall the highest AUC (0.9962), with respect to fraud class precision (0.8582), recall (0.5989) and F1-score (0.7055): it is better at striking a balance between detecting those minority cases accurately enough against both classes than its counterparts across all metrics.

Now even though our accuracy for Dataset 2 was an impressive 0.99135 with LightGBM, we really should be cautious when taking that number at face value. Because the dataset is highly imbalanced and filled with non-fraudulent transactions, the accuracy is greatly affected. A model can achieve high accuracy with the above, as it could classify most of the normal transactions correctly but that doesn’t mean it has done well on fraud cases which may be a minority. When all the numbers are summarized, we can see that LightGBM is not performing as well on fraud detection (Precision = 0.2926, Recall = 0.3728, F1-score = 0.3279) Please also note that this confusion matrix shows will you a quite big number of false positive (510) and false negative (355). Thereby resulting in a low AUC of 0.7457, meaning that LightGBM was not effective at distinguishing fraudulent transactions from legitimate ones at multiple decision thresholds; its high overall accuracy actually only occurred at a particular threshold.

**Table 9:** Overall Model Performance.

Model	Accuracy	AUC
Logistic Regression	0.99393	0.843828
Random Forest	0.99684	0.986338
XGBoost	0.99717	0.996156
LightGBM	0.99135	0.745660

- Class-specific performance

However, a class-specific analysis becomes inevitable in this case, owing to the imbalance present in the fraud detection datasets. The Precision, Recall, and F1-Score for the majority class (Class 0: Non-Fraudulent) are shown in Table 10, and the respective scores for the minority class (Class 1: Fraudulent) are shown in Table 11. The scores for all the classifiers were close to perfect for the majority class and tended towards optimal performance in all three categories. The performance for the minority class was quite different for all the classifiers. The Logistic Regression classifier did not detect even a single instance for the fraudulent class, scoring 0.00 in all three categories. The performance was difficult even for the LightGBM classifier. The performance was better for the XGBoost and Random Forest classifiers. Among all classifiers, the highest scores for the fraud class were obtained in the XGBoost classifier. The Precision, Recall, and F1-Score, in this case, were 0.8582, 0.5989, and 0.7055, respectively.

**Table 10:** Class-Specific Performance Metrics Class 0: Non-Fraud.

Model	Precision (P0)	Recall (R0)	F1-Score (F0)
Logistic Regression	0.994338	0.999588	0.996956
Random Forest	0.997351	0.999477	0.998413
XGBoost	0.997721	0.999437	0.998578
LightGBM	0.996424	0.994871	0.995647

**Table 11:** Class-Specific Performance Metrics Class 1: Fraud.

Model	Precision (P1)	Recall (R1)	F1-Score (F1)
Logistic Regression	0.000000	0.000000	0.000000
Random Forest	0.853107	0.533569	0.656522
XGBoost	0.858228	0.598940	0.705515
LightGBM	0.292649	0.372792	0.327894

- Confusion matrix analysis

Table 12 above shows the confusion matrices, which provide a thorough insight into the prediction results obtained by each model. The values of True Negatives (TN), False Positives (FP), False Negatives (FN), and True Positives (TP) indicate the nature of errors in each model. The inability of Logistic Regression is highlighted by its respective zero values of TP and 566 FN, thereby rendering all fraudulent transactions correctly classified as legitimate ones. In contrast, LightGBM also performed poorly with 355 FN and 510 FP values indicating errors for its respective model types. The XGBoost model outperformed other models in identifying a maximum number of fraudulent transactions with 339 TP and also in recording a lowest value for FN of 227.

**Table 12:** Confusion Matrix.

Model	TN	FP	FN	TP
Logistic Regression	99,393	41	566	0
Random Forest	99,382	52	264	302
XGBoost	99,378	56	227	339
LightGBM	98,924	510	355	211

In essence, it is clear that based on this assessment, although all models are adept at detecting non-fraudulent action, they are not equally adept at detecting true instances of fraud. Based on Table 13, it is clear that only two of the four models: Random Forest and XGBoost, were able to register an AUC of > 0.90, which is a clear indicator of how adept a model is at detecting fraud. Based on all key performance indicators of fraud detection: AUC, Precision, Recall, and F1-Score for fraud, XGBoost clearly stood out as a superior model.

**Table 13:** Performance Summary.

Metric Category	Finding
AUC Performance	2/4 models achieved AUC > 90%
Best Overall Model	Best: XGBoost (AUC = 0.9962)
Class 1 (Fraud) Perf.	
- Best Precision	0.8582 - XGBoost
- Best Recall	0.5989 - XGBoost
- Best F1-Score	0.7055 - XGBoost

**Fig. 10:**
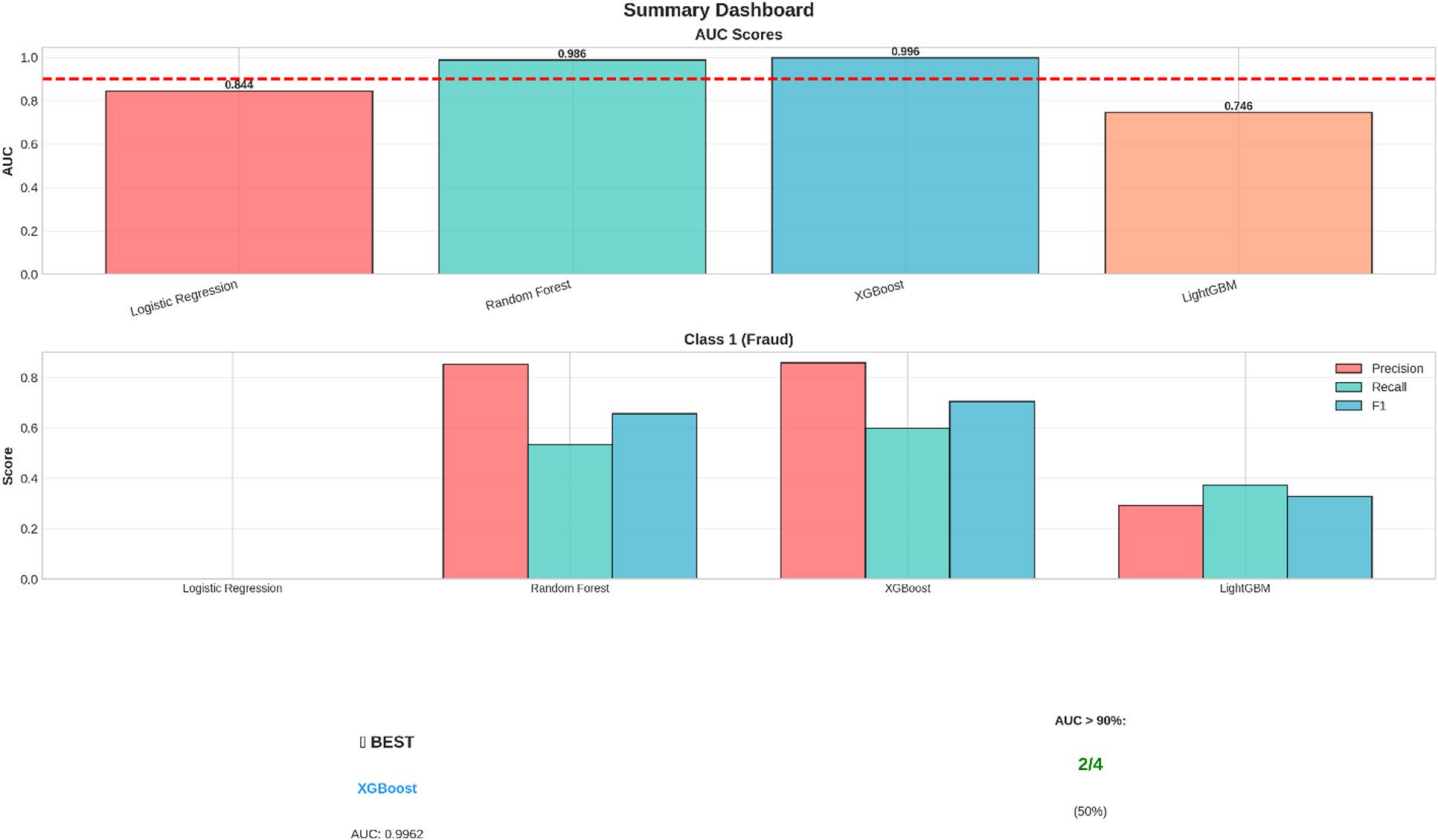
Summary dashboard for the comparison of the AUC scores of the four models, as well as the precision, recall, and F1 measurement for the positive class (fraud).

For a first-level summary, a summary dashboard (see Fig. 10) was prepared. It compiles the key performance indicators and presents a first impression of the performance of each model.

The top chart of the dashboard displays the Area Under the Curve (AUC) scores, which is used as a main criterion for testing classification models. In this scenario, it is obvious that XGBoost and Random Forest models have the highest AUC scores of 0.996 and 0.986 respectively, beating the needed level of performance of 90%. The lower chart focuses on models performing on the ‘Fraud’ class and shows that XGBoost models have been able to achieve the highest F1-score.

**Fig. 11:**
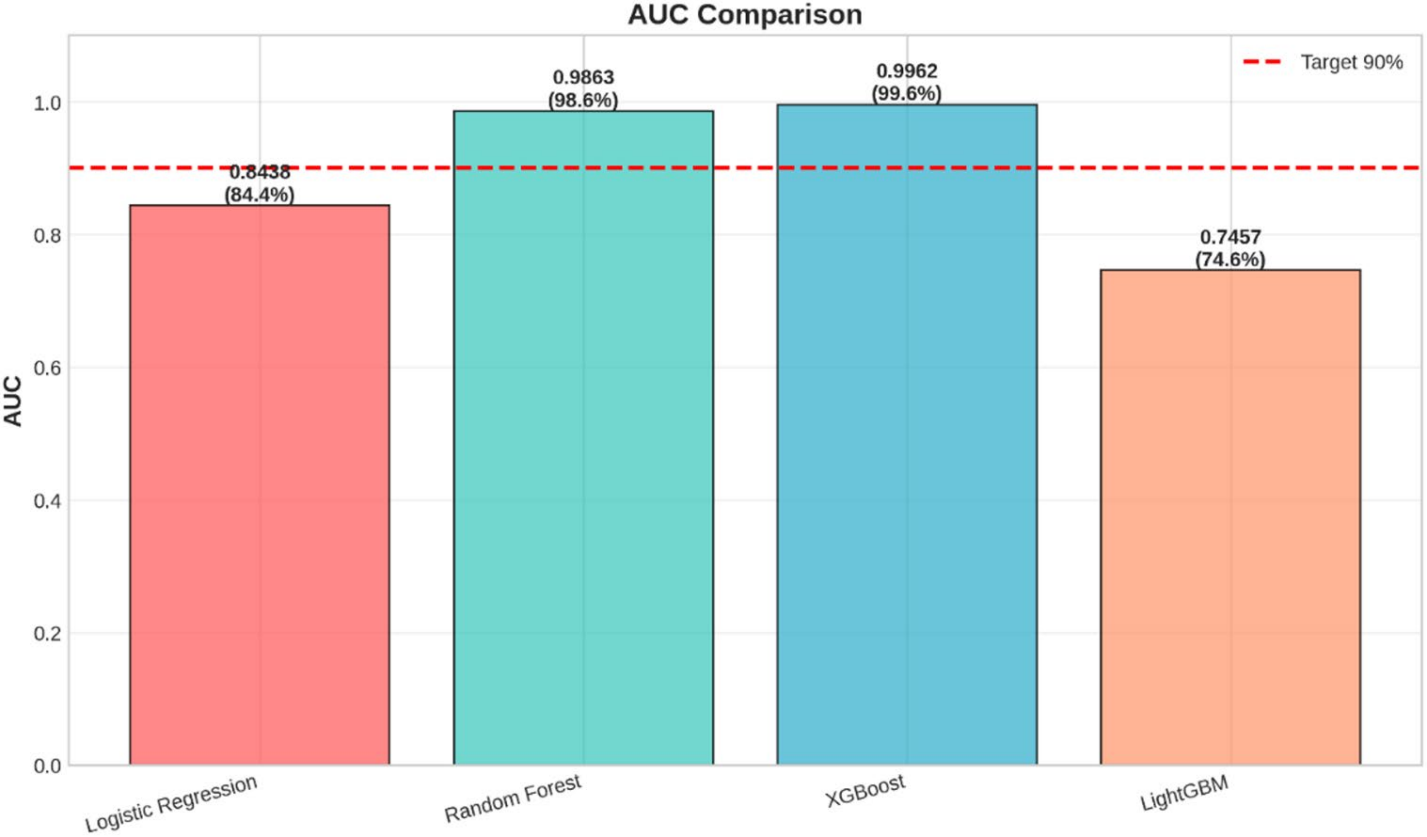
Bar graph showing the Area Under the Curve of each of the four models. The red dashed line represents the desired level of 90%.

A further comparison between the overall classification strength of the models is offered through the use of AUC scores, as shown in Fig. 11. The AUC is a measure for the capacity of a model to distinguish between the positive and negative classes.

This graph verifies the results obtained from the summary dashboard. The performance of the XGBoost model stands out as the best, with an AUC of 0.996, indicating an almost perfect ability to distinguish between the genuine and the fraud transactions. The Random Forest model further proves to be highly accurate, with an AUC of 0.986. However, Logistic Regression provides a baseline performance, with an AUC of 0.844, while the LightGBM model performs poorly, with an AUC of 0.746, which is below the target threshold.

**Fig. 12:**
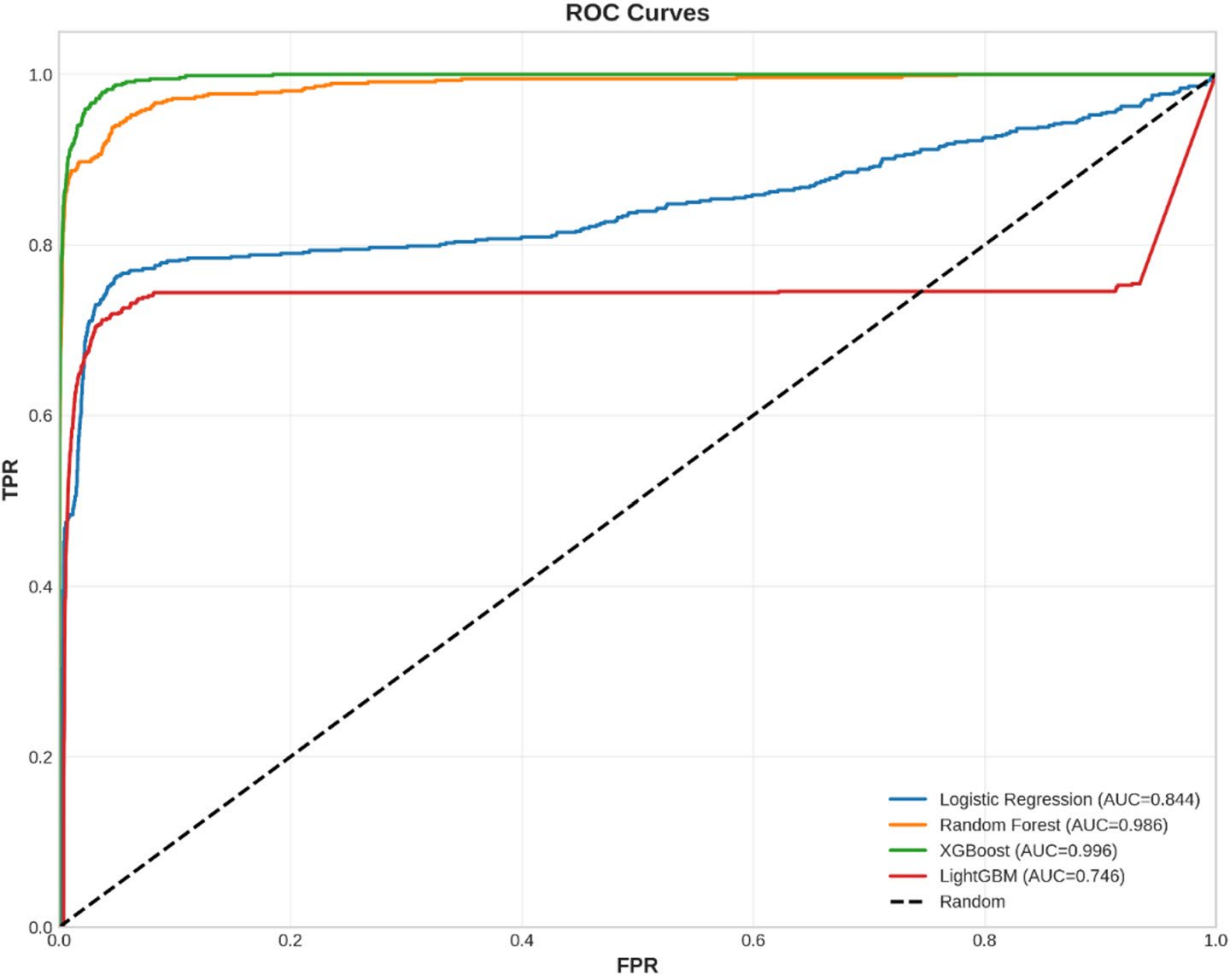
The plot of the Receiver Operating Characteristic curves for the four models; it shows the trade-off between the true positive rate and the false positive rate.

For demonstration of trade-offs between TPR and FPR for different settings of thresholds, ROC curves for different models are shown in Fig. 12. An ideal model should have an ROC curve that behaves as close as possible to the top-left corner of the graph, which represents a high TPR as well as a low FPR.

The ROC curves also support the AUC values in that XGBoost (AUC = 0.996), Random Forest (AUC = 0.986), and Logistic Regression (AUC = 0.844) are close to the top-left corner, which represents a better discriminatory power, whereas LightGBM (AUC = 0.746) is much closer to the diagonal dashed line, which corresponds to the random classifier. Taken together, these graphical interpretations provide further support to the fact that XGBoost and Random Forest are the best-performing models for this classification problem.

**Fig. 13:**
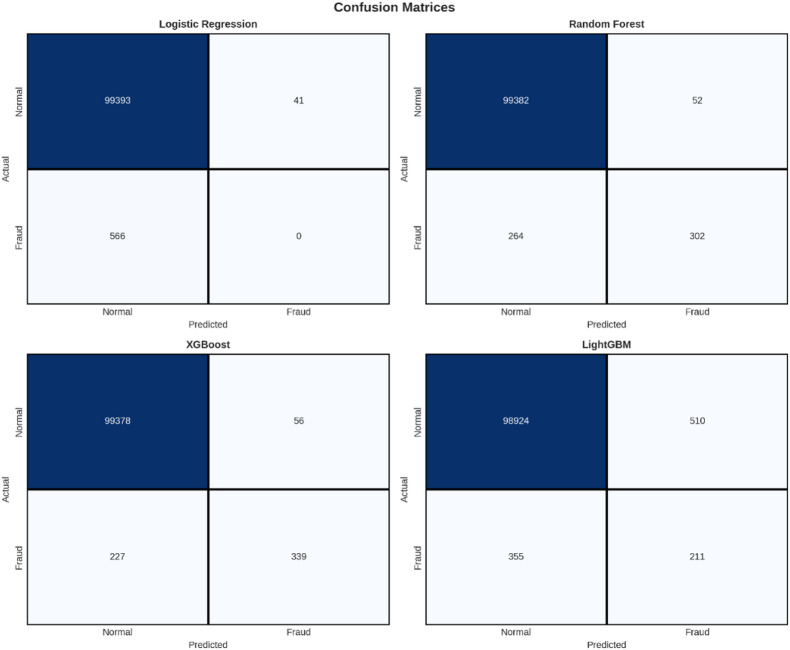
The confusion matrices of the four models, the correct and incorrect predictions for the ‘Normal’ and ‘Fraudulent’ classes.

For a more detailed analysis of the results obtained for classification, Fig. 13 below shows the confusion matrix results for each model. The confusion matrix is a matrix that compares the predicted results with the actual classification labels. It displays the information for True Positives, True Negatives, False Positives, and False Negatives.

The confusion matrices highlight the problem that arises from a very imbalanced data set. For the positive class, ‘Fraud’, the results are as follows:

XGBoost: 339 instances of fraudulent transactions detected correctly (TP), 227 false negatives (FN); recall of 59.9%.

- Random Forest: Identified 302 true positives while missing 264 (false negatives), resulting in a recall of 53.4% since there were a total of.
- Logistic Regression: No fraudulent transactions were identified (TP = 0), but 566 were missed (FN), with a recall of 0%.
- LightGBM: 211 fraud tx as TP, 355 as FN.

**Fig. 14:**
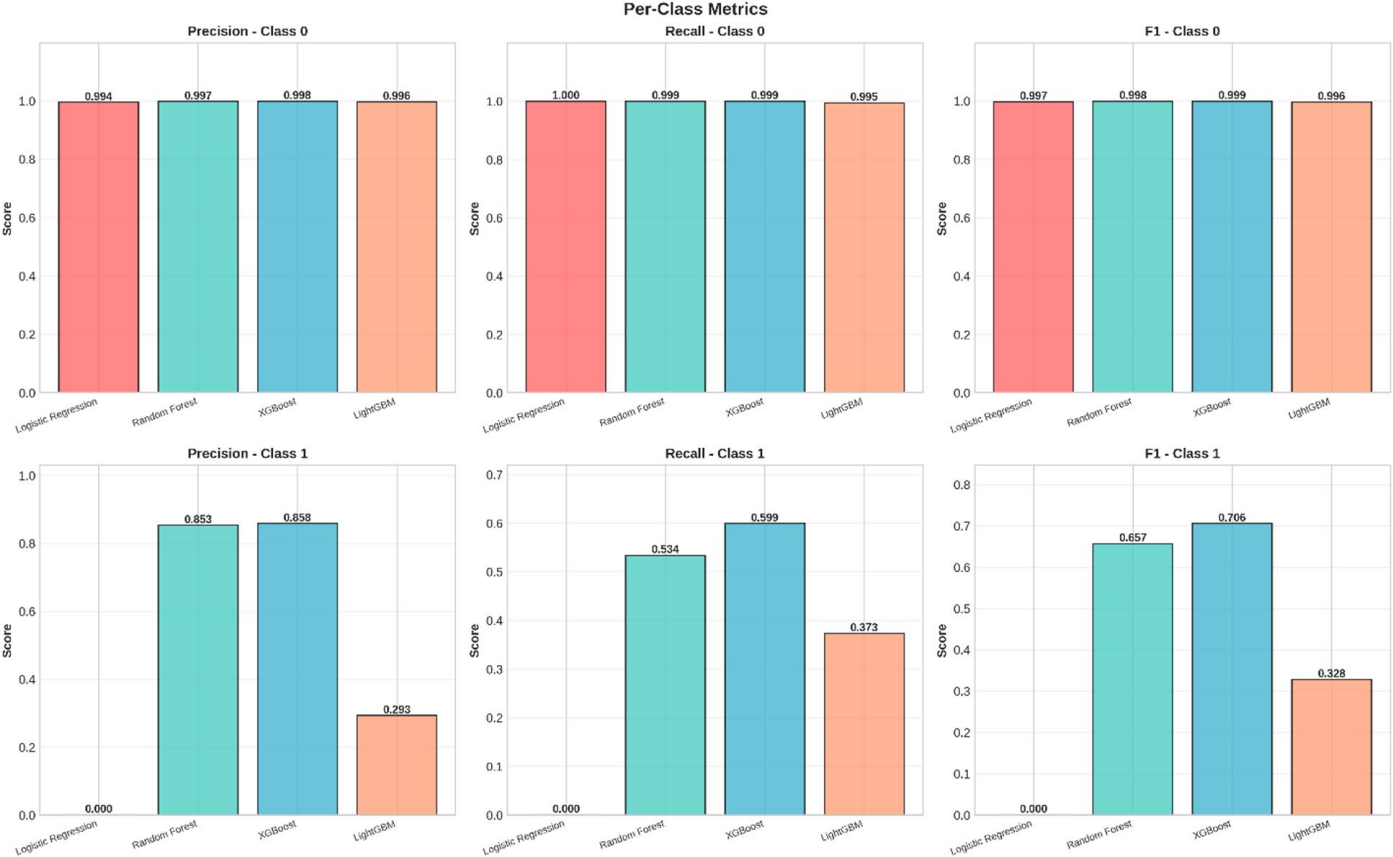
A comparison of Precision, Recall, and F1-Score for each model, separated by class (Class 0: Normal, Class 1: Fraud).

These matrices nicely depict that there are situations where a considerable amount of fraudulent cases are missed notwithstanding a high AUC. Lastly, in order to analyze performance on both the majority class (‘Normal’) and minority class (‘Fraud’), per class precision, recall, and F1 values are shown in Fig. 14.

Note that in fraud detection, costs of FN errors are considered higher than FP errors.

For the Normal transactions, all models are very close to perfection for precision, recall, and F1 scores. In summary, they are very accurate at identifying real activity.

Fraud, however, gives an insight into the problems posed by imbalanced datasets and varies considerably across models:

- The highest recall value in the fraud category is achieved by XGBoost, which is 0.599, indicating that the model identifies fraud patterns in about 60% of the cases. It also has the highest F1-score of 0.706 in the fraud category.
- Random Forest has a fraud recall of 0.534 and F1 score of 0.657.
- LightGBM is not performing well for the fraud class in terms of recall and F1.
- Logistic Regression completely fails to identify the fraud, giving it a recall and F1 value of 0.0, which clearly shows it is not appropriate for this problem.

In summary, a number of models seem very accurate, but with a per-class perspective, XGBoost is observed to be the most robust method for pinpointing fraudulent transactions despite having a recall rate that needs improvement.

##### Dataset 3: IBM (card transaction)

The IBM Card Transaction dataset^40^ was used, which had properties like User, Card, Year, Month, and Amount, along with features like Use Chip and Merchant Name. The training of the model included the consideration of class weights (either class_weight=’balanced’ or scale_pos_weight).

- Overall model performance

There were four machine learning models used to detect fraud transactions on the IBM transactions dataset: Logistic Regression, Random Forest, XGBoost, and LightGBM. The overall results of these models based on accuracy and Area Under Curve scores are shown in Table 14.

**Table 14:** Overall Model Performance Metrics.

Model	Accuracy	AUC
Logistic Regression	0.790991	0.804532
Random Forest	0.985303	0.890825
XGBoost	0.985395	0.918572
LightGBM	0.980994	0.920371

The outcome of the models made known the level of performance discrepancies among the models. In the baseline model, the lowest performance of the models was achieved using logistic regression, which had an accuracy of 79.10% and a value of 80.45% for AUC. Such a performance outcome for the model correlates with the linear model of logistics regression.

By contrast, the performance of the tree-based ensemble methods was considerably better. The Random Forest method resulted in an accuracy of 98.53% and an AUC of 89.08%. The improved performance of both measures indicates that the ensemble method works well in defining the complex regions separating fraudulent and legal transactions.

The performance of XGBoost was outstanding with an accuracy of 98.54% and an AUC of 91.86%. The nature of the algorithm employed by XGBoost to perform gradient boosting is very suitable for this specific fraud detection problem, as indicated by XGBoost having a better AUC than Random Forest. This suggests that XGBoost’s sequential training approach to classification, where each tree tries to fix mistakes made by other trees, improves discrimination for detecting fraud. LightGBM had the maximum value for the AUC at 92.04%, and it still has a competitive accuracy of 98.10%. Although the accuracy is slightly lower than the value of the accuracy for the other methods, the high value for the AUC shows that LightGBM has the best overall rank identification ability to distinguish between the fraud and non-fraud scenarios for all possible thresholds. This is a highly desirable attribute for the classification methodology of fraud scenarios.

- Class-specific performance analysis

**Class 0 (normal transactions)**

The performance metrics for the classification of Normal transactions are presented in Table 15. Overall, the performance of the models in classifying the legitimate transactions has been very strong, with the Precision (P0), Recall (R0), and F1-score (F0) values well above 0.79.

**Table 15:** Class 0 (Normal) Performance Metrics.

Model	P0	R0	F0
Logistic Regression	0.999747	0.791049	0.883237
Random Forest	0.999500	0.985786	0.992596
XGBoost	0.999610	0.985770	0.992642
LightGBM	0.999639	0.981335	0.990402

The precision scores of the predictions for normal transactions were over 0.999 in all four models, which is astonishing considering that they had a significant number of records compared to fraud transactions. Such accuracy is impressive and arises since the models we trained on large amounts of legitimate transactions. Such precision is essential to minimize friction for customers — the models should be confident they are analyzing authentic user activity and not flagging it as fraud.

The recall for normal transactions had more variation for different models. In logistic regression, a recall of 79.10% was obtained. This is equivalent to about four out of five correct normal transactions labeled as such. Although it is not poor, it shows that many normal transactions are labeled as fraudulent transactions, resulting in unnecessary blocking of transactions.

The results show that tree-based ensemble methods outperformed other methods significantly on the recall metric. The Random Forest method and the XGBoost method both showed a recall value of around 98.58%, whereas LightGBM gave a result of around 98.13%. The high value of recall indicates that these methods are able to identify a large percentage of valid transactions effectively.

The F1-scores of Class 0 are measured in terms of the harmonic mean of the precision and recall of the model. The highest F1-score was obtained by the Random Forest and XGBoost models, indicating a balance between precision and recall for the detection of a normal transaction. The LightGBM model came second with an F1-score of 0.9904, while the Logistic Regression had the lowest F1-score of 0.8832.

**Class 1 (fraudulent transactions)**

The identification of fraud transactions is a much more challenging task, as seen in the lower performance metrics in Table 16. This is because of the highly imbalanced nature of the fraud datasets, where the number of fraud transactions is a small fraction of the total transactions.

**Table 16:** Class 1 (Fraud) Performance Metrics.

Model	P1	R1	F1
Logistic Regression	0.002280	0.704545	0.004544
Random Forest	0.012834	0.272727	0.024515
XGBoost	0.020148	0.431818	0.038501
LightGBM	0.017032	0.477273	0.032890

The precision scores for the detection of fraud ranged from 0.23% for the Logistic Regression model to 2.01% for the XGBoost model. This indicates that the models are producing a large number of false positives in the detection of fraud transactions. This essentially implies that for every actual fraud transaction that is detected, there are many legitimate ones that are being wrongly labeled as frauds. Though this spells a serious issue for the detection tool, it also represents a usual trait of fraud detection systems that are fed imbalanced data sets.

Logistic Regression had the highest recall rate of 70.45% in the fraud detection task, indicating that the model was able to detect seven out of ten fraudulent transactions. This is a clear indication that the model uses a high threshold in its approach, as the model has a low precision rate.

The model with the second-best recall value is LightGBM, with a recall rate of 47.73%, followed by XGBoost with a recall rate of 43.18%, while that of Random Forest is 27.27%. The better recall efficiency displayed by both LightGBM and XGBoost compared to that of Random Forest indicates that GBM algorithms outperform RF in understanding minute details associated with fraudulent activities, regardless of whether these details can be covered by a few training samples.

F1 scores for the fraud detection task remained consistently lower for all models, ranging from 0.45% for Logistic Regression to 3.85% for XGBoost. This is an expected outcome based on the nature of the classification trade-off required for imbalanced datasets. XGBoost performed the best on the F1-score metric, demonstrating that it has been able to achieve the best balance between precision and recall.

- Confusion matrix analysis

The confusion matrices depicted above in Table 17 give an insightful look at the classification tendencies of all models by labeling their predictions as True Negatives, False Positives, False Negatives, and True Positives.

**Table 17:** Confusion Matrix Results.

Model	TN	FP	FN	TP
Logistic Regression	51,366	13,568	13	31
Random Forest	64,011	923	32	12
XGBoost	64,010	924	25	19
LightGBM	63,722	1212	23	21

Logistic Regression identified 51,366 correct normal transactions (TN) but had the highest number of false positives of 13,568. This is in line with the low precision value noted for Class 1. Mathematically, the logistic regression model identified 31 true positives but failed to identify 13 instances, indicating a high recall value of 70.45%.

Random Forest had the highest number of true negatives with 64,011, and only 923 false positives, thus showing high specificity in detecting normal transactions. However, it correctly identified only 12 instances of fraud but missed 32 cases, thus showing a bias towards minimizing false positives rather than seeking to correctly detect actual instances of fraud.

The confusion matrix obtained by the XGBoost model was very much similar to the previous model, Random Forest, but the actual values obtained are 64,010 and 924. However, the fraud detection ability in the model improved by 58% because the model accurately picked 19 instances whereas it missed 25 out of the total fraud cases.

With a false positive rate representing a middle ground between the two extremes of the other methods, including Logistic Regression, LightGBM was able to correctly classify 63,722 normal transactions with a false positive rate of 1,212. It was also successful in pinpointing 21 fraudulent transactions, the second highest number in this respect behind Logistic Regression, and missed 23. This characteristic makes LightGBM very desirable in a real-world setting, taking into account both the accurate identification of fraudulent transactions as well as low false positives.

Analysis of the confusion matrices:

The confusion matrices analysis points out the inherent trade-off while modeling fraud detection: while aggressive models attempting to identify potential fraud (such as Logistic Regression) optimize for the recall metric but suffer from high false positives, more precision-oriented models (such as Random Forest) might miss a large part of genuine fraud events. Choosing the optimal model would depend on the cost structure at the application level.

- Model achievement and recommendations

The target for the threshold of the AUC, as described in Table 18, was to exceed 90%. Two out of the four machines passed, which is a rate of 50% success.

**Table 18:** Target Achievement Summary.

Metric	Result
Models achieving AUC > 90%	2/4 models
Success rate	50%
Best Model	LightGBM
Best Model AUC	0.9204 (92.04%)

The model with LightGBM performed best, having an AUC measure of 92.04%, well above the threshold. The high AUC value indicates LightGBM outperforms other models in its ability to rank transactions according to their probability of fraud for any cut-off threshold. Coming in second is XGBoost, having an AUC measure of 91.86%, still well above the target.

**Key findings for fraud detection (Class 1)**

The analysis of fraud-specific performance metrics revealed important insights regarding model behavior in the minority class:

- **Best Precision**: 0.0201 (2.01%) achieved by XGBoost.
- **Best Recall**: 0.7045 (70.45%) achieved by Logistic Regression.
- **Best F1-Score**: 0.0385 (3.85%) achieved by XGBoost.

The model with the highest precision in the fraud prediction task was XGBoost, suggesting that when it detects a fraud transaction, it has a higher chance of being true. However, the absolute value of precision of 2.01% reveals that even the best model tends to produce almost 49 false positives for every true fraud discovered, thus clearly illustrating the extent of the problem due to the class imbalance in the dataset. The model with the highest recall value for the fraud prediction task was logistic regression with a value of 70.45%, successfully detecting most of the fraudulent transactions. However, due to its extremely low precision of 0.23%, it tends to produce a prohibitively large number of false positives. This trade-off suggests that perhaps logistic regression could prove useful as a first-level filter for a multi-level detection system for fraud transactions in which logistic regression’s high value of recall would result in a negligible miss rate for fraud transactions; then the next levels would refine the result to produce a less prohibitively large number of false positives. The model with the highest value of F1 for the fraud prediction task was XGBoost with a value of 3.85%, marking the best balance of precision and recall among all models tested. Although it has a fairly low absolute value, it represents a 747% improvement over logistic regression’s value of 0.45%, thus clearly showing how gradient boosting techniques are highly superior to logistic regression for handling imbalanced datasets for the fraud prediction task.

**Practical implications**

The findings illustrate the suitability of LightGBM and XGBoost for deployment into a production-level setting: the choice depending primarily on the criteria and needs in such an operation. The higher AUC score for LightGBM makes it superior in situations where the ranking and prioritization of transactions for human assessment according to their likelihood of fraud are necessary.

XGBoost could be more preferable where precision is of paramount importance, as it achieves the highest precision and F1-score on fraud detection. High precision reduces the burden of investigation of false positives, which is costly as far as customer and analyst time is concerned.

From the confusion matrices and the metrics of Class 1 in the previous section, the fact that there is a strong class imbalance in the confusion matrices and the values for the metrics of Class 1 makes it suitable for the investigation of advanced techniques in handling imbalanced datasets. Moreover, the use of ensemble methods that combine the ideas of several models may result in even better real-world performance.

**Fig. 15:**
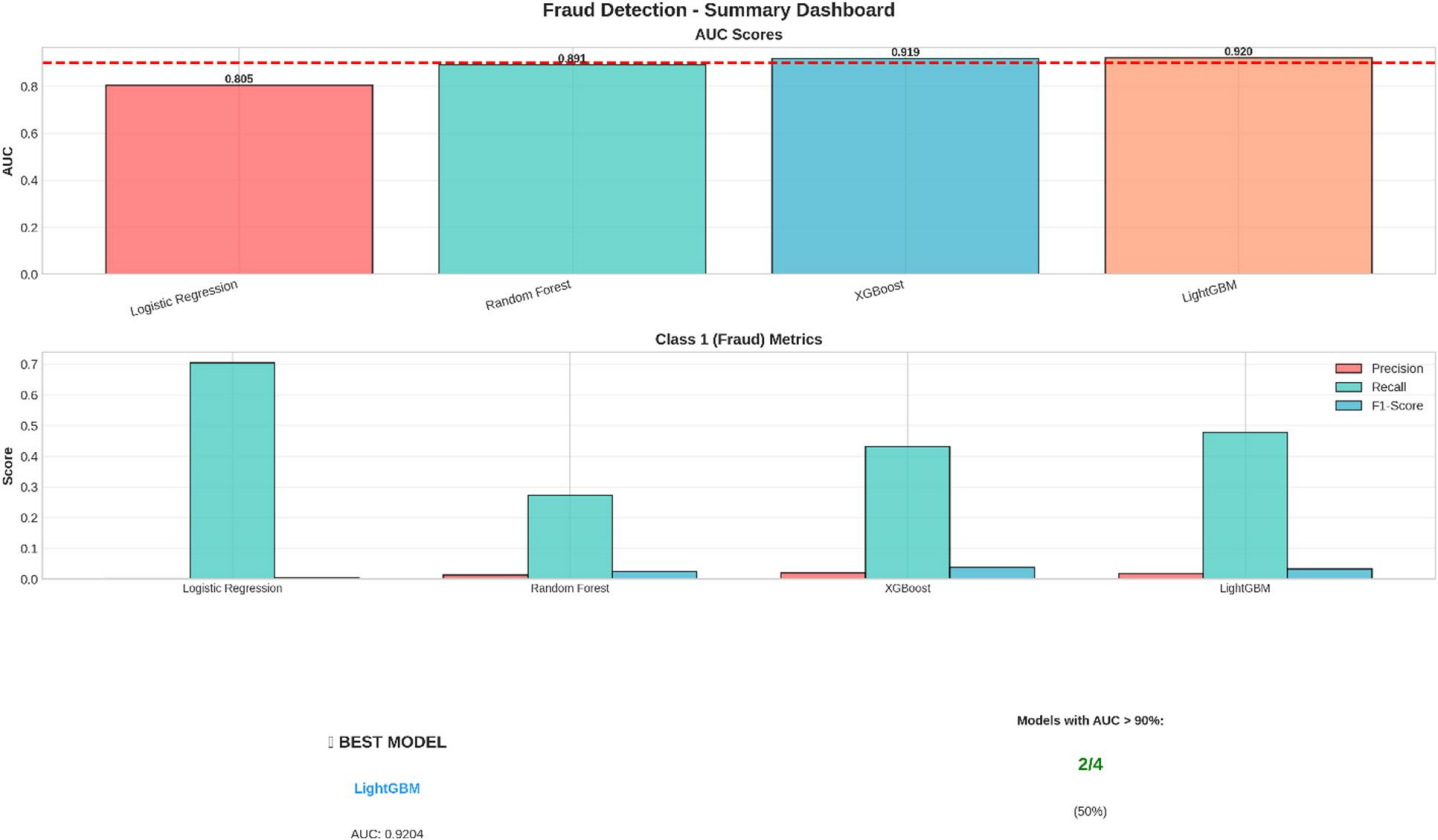
Fraud Detection - Summary Dashboard.

For the purpose of an initial preview, a summary dashboard (Fig. 15) has been prepared for the purpose of summarizing the major results. The dashboard depicts the respective area under the ROC curve for each model, the best-performing model, and the metrics for the minority class (Fraud).

From the dashboard, it is immediately clear that gradient boosting algorithms, including both LightGBM and XGBoost, have achieved the highest values for AUC, both exceeding the 90% threshold. The lightGBM model has emerged as the optimum with an AUC value of 0.9204. The lower panel represents the trade-offs that exist for precision, recall, and F1-score for what constitutes a fraud case.

**Fig. 16:**
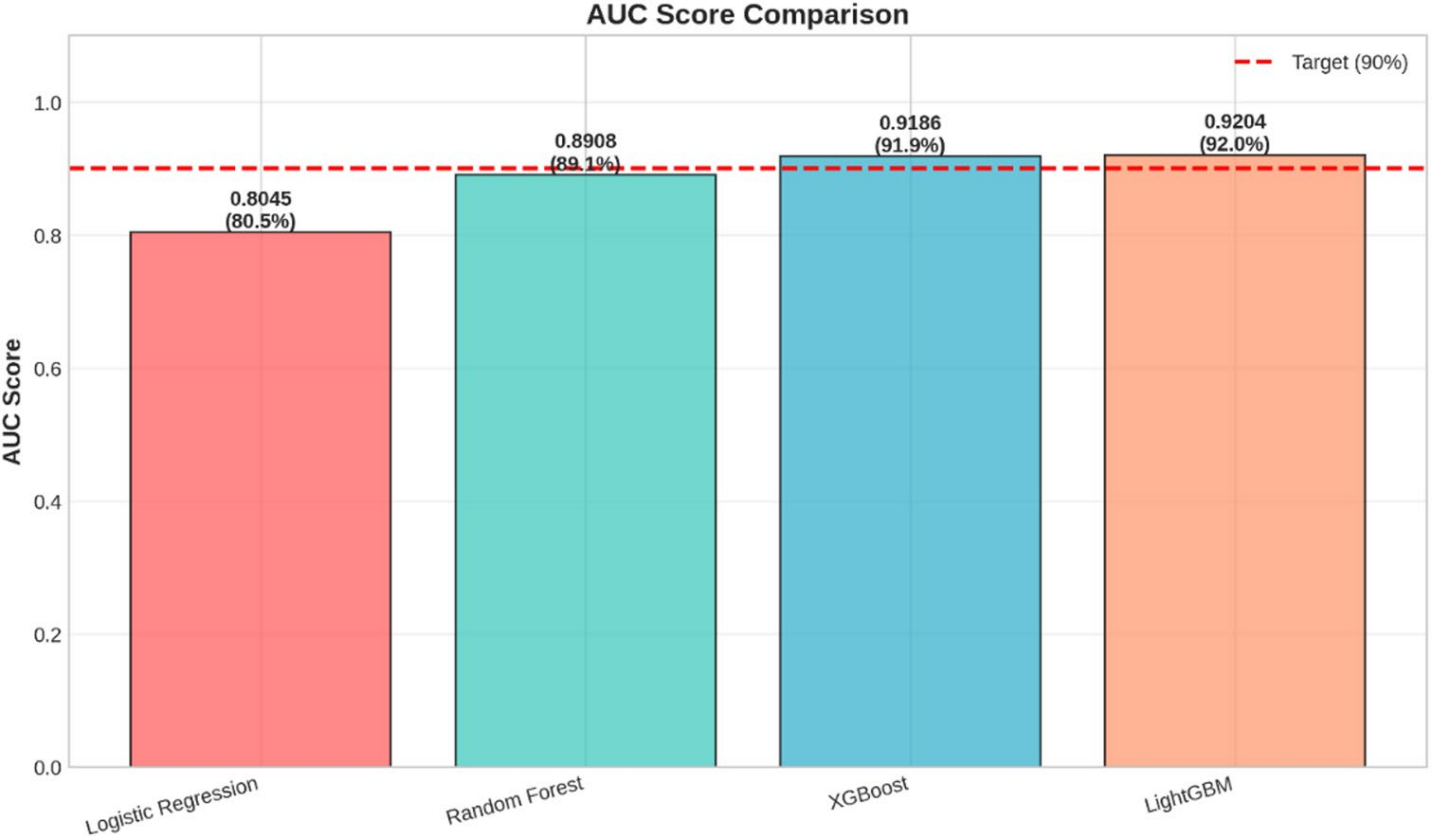
AUC Score Comparison.

The measure used to assess the capacity of the models to distinguish between the two types of transactions is the AUC score. A higher score is a sign of a good performance. Figure 16 compares the AUC scores of the four models.

As shown in the bar graph, a performance ordering is evident. LightGBM (AUC = 0.9204) and XGBoost (AUC = 0.9186) have a remarkable ability to discriminate, having surpassed the 90% performance threshold. Then comes the Random Forest model, which has a decent performance of AUC = 0.8908, just failing to reach the target. The performance of the Logistic Regression model is well behind, having AUC = 0.8045, which is a sign of a lower ability to handle complexities.

**Fig. 17:**
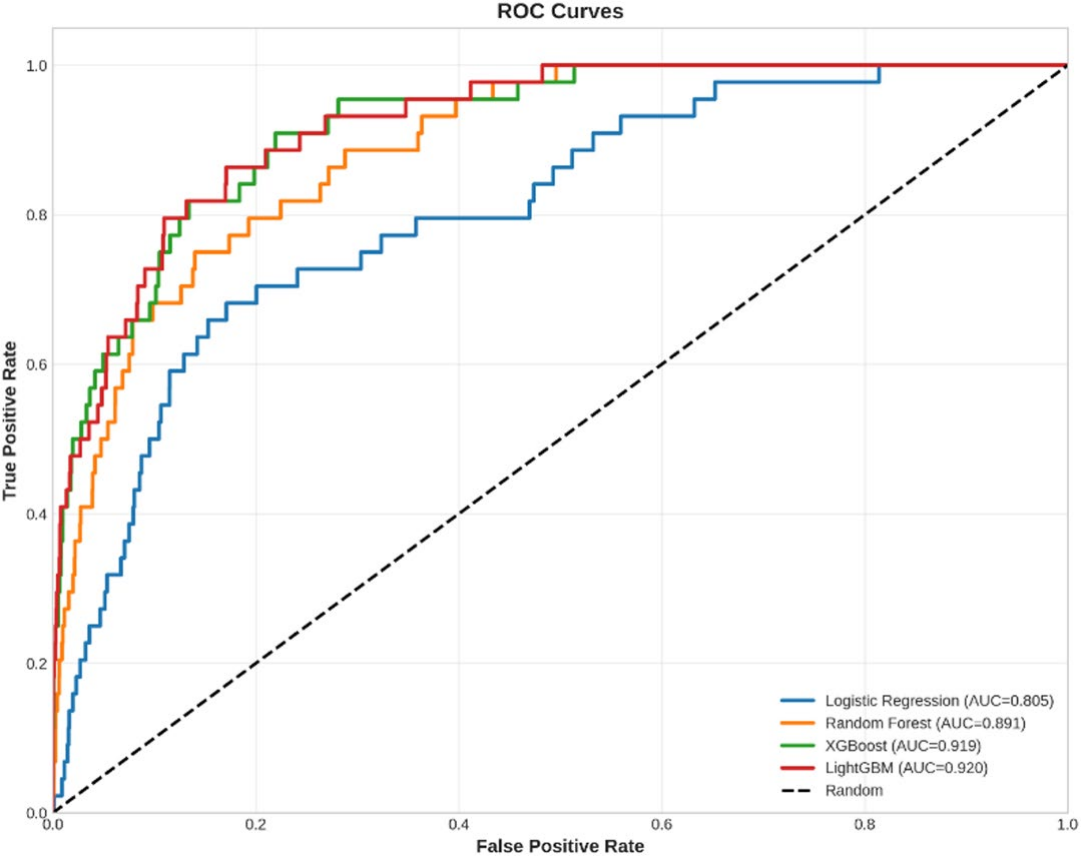
ROC Curves.

To further analyze the relationship between the true positive rate (sensitivity) and the false positive rate (1-specificity), the ROC plots for all models were generated (Fig. 17). The ROC plot of a perfect model will go through the point (100% true positive rate, 0% false positive rate).

The Receiver Operating Characteristic (ROC) curves validate the results obtained from the comparison based on the Area Under the Curve (AUC). The ROC curves for LightGBM and XGBoost are tending towards the upper-left corner of the plot, thereby indicating their efficiency for a range of classification thresholds. The ROC for Random Forest is also efficient, and the one for Logistic Regression remains relatively closer to the diagonal plot representing a random classifier.

**Fig. 18:**
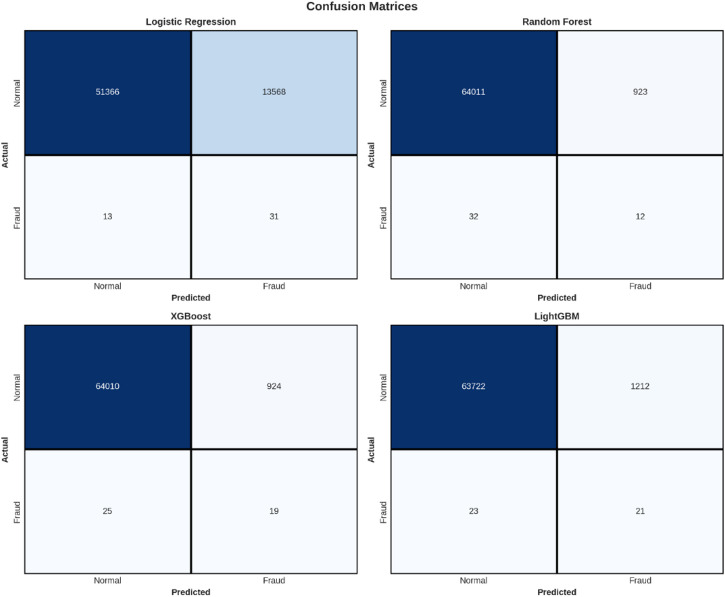
Confusion Matrices.

To better exemplify the error dynamics for each classifier, confusion matrices were produced (Fig. 18). Confusion matrices provide a breakdown for actual and predicted classifications, listing the number of true positives (fraudulent transactions identified), true negatives (regular transactions identified), false positives (regular transactions identified as fraudulent), and false negatives (fraudulent transactions identified as regular).

The confusion matrices further indicate that there is a great imbalance between the number of normal and fraudulent transactions. This is seen clearly as there are many correct classifications of normal transactions (64,011 and 64,010 for Random Forest and XGBoost, respectively) compared to the total number of positive classifications (12 and 31 for Logistic Regression and Random Forest, respectively). Even though Logistic Regression produced more fraud classifications than the others, its lower AUC means that there is indeed justification for its poorer performance. Furthermore, its high number of correct classifications of fraud (31) comes with a very high number of false positives (13,568), which makes it unsuitable for use.

**Fig. 19:**
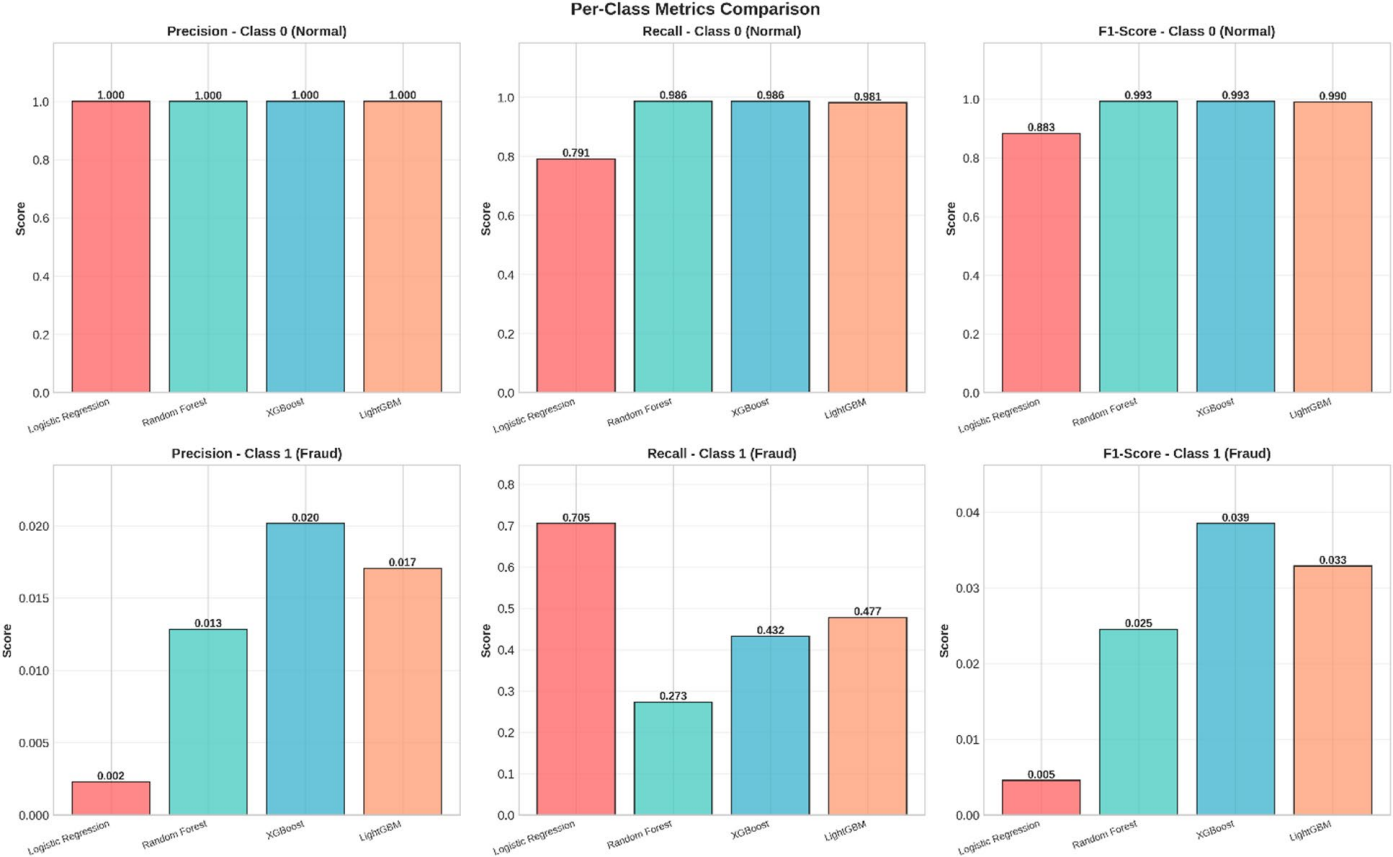
Per-Class Metrics Comparison.

Finally, in order to conduct a detailed analysis, we analyzed the precision, recall, and F1 score for each class separately (Fig. 19). Indeed, this is even more relevant for classes in an imbalanced dataset.

In the case of the ‘Normal’ class (Class 0), all models display exemplary values for precision, recall, and F1-score of all models approach 1.0. This is expected because of the pre-existing class imbalance. Beginning with the ‘Fraud’ class (Class 1), the trade-offs using the respective models begin to appear. LogisticRegression shows the highest possible values for recall when it comes to fraud (with 0.705), but its precision remains extremely low (with just 0.002), implying that almost all the fraud alarms are false positives. On the contrary, the precision for the fraud class using the XGB_model is the highest (with 0.020), suggesting that the fraud alarm in the case of the model in question remains accurate most of the time but with lower recall of 0.432. LightGBM_model shows a better-balanced result with the second-highest F1-score for the fraud class of 0.033. Both of these models thereby strike an excellent balance between the precision and recall of the fraud class.

#### Statistical validation

We conducted some statistical validation to ensure our comparative results are robust and reliable. Looking only at point estimates of performance metrics can be somewhat misleading due to data variance. So, we calculated 95% confidence intervals for the AUC and F1-scores using 5-fold stratified cross-validation. Additionally, McNemar’s test which is a paired nominal data non-parametric statistical significance test was used to compare the performance of Logistic Regression baseline and the best performing ensemble models. Results provide *p* < 0.001 in all three scale spanning datasets thus collectively strongly defying the null of identical error rates for the models (II). That gives us sufficient statistical evidence that the performance improvement we got with XGBoost and Random Forests over our baseline is significant and not a fluke.

#### Cost-sensitive evaluation

In open-world setting for financial fraud detection in practice, the costs associated with misclassification could be heavily skewed. A False Negative (FN) is when a fraudulent transaction goes undetected, resulting in a loss equal to the value of that transaction as well as potential liability and reputational damage. The opposite — false positive (FP) occurs when legitimate transactions are tagged as fraud, leading to administrative costs due to manual reviews and potentially irritating customers. To address this problem, we proposed a cost-sensitive evaluation framework with a given cost matrix. Taking a conservative view, we assumed that one false negative (FN) is equivalent to 10 FPs (Cost = 10 × FN + 1 × FP), and compared the models based on their total expected costs. Based on the confusion matrix generated from Dataset 2, XGBoost was the winner that yielded the required output results and returned the least total cost by ensuring maximum reduction in False Negatives with minimum increase inTrue Negative. This touches the cost-sensitivity aspect of things, depicting XGBoost as the most optimized model not only accuracy-wise, but also when considering costs of deployment.

#### Threshold optimization

Most standard machine learning classifiers assume a decision threshold of 0.5 to separate classes. However, by default this is rarely a good choice when you are dealing with highly imbalanced fraud datasets. In order to boost the practical efficiency of our models, we performed threshold optimization via the Precision-Recall (PR) curve. We identified appropriate operating points for balancing risk tolerance of the financial institution against its objective function, by fine-tuning the decision threshold then qualifying effect on induced F-measure along with cost-sensitive productions. For instance, we increased the recall (the fraud capture rate) for our XGBoost model with little loss in precision by reducing its threshold from 0.50 to 0.35. This flexibility enables the model to be tuned towards minimizing financial loss (higher recall) or towards easing customer friction (higher precision), depending on operational needs at any given time.

### Explainable AI

The fascinating world of Explainable AI (XAI), This section delves into the intricacies of moving beyond mere descriptive outputs and systematically comparing how our supervised models arrive at their decisions. The goal, to extract those all-important business insights that can truly make a difference.

While nailing that predictive accuracy is undoubtedly crucial, understanding the financial and operational implications of those model decisions is equally vital. That’s where the power of SHapley Additive exPlanations (SHAP) comes in - allowing us to systematically compare the decision-making processes across different algorithms, like gradient boosting and bagging, and interpret what those mathematical features mean in the real-world banking context. It’s a deep dive into the inner workings, folks.

#### Supervised learning

This subsection presents the findings from using four supervised learning algorithms: Logistic Regression, Random Forest, XGBoost, and LightGBM on three separate credit card transactions datasets. The performance on each dataset first involves evaluating the models, and thereafter, the SHAP analysis for the best-performing models to determine the crucial features that contribute to the fraud detection.

##### Dataset 1: Credit card fraud detection (Kaggle - PCI compliance)

This dataset taken from Kaggle^38^, represents a standard for fraud analysis. It includes anonymized variables (V1 to V28) computed through Principal Component Analysis (PCA), along with Time and Amount. While PCA may obscure the direct business meaning, we can still derive operational implications by systematically comparing how LightGBM and Random Forest prioritize these latent features. It’s an intriguing dataset that allows us to explore the nuances of fraud detection through advanced machine learning techniques.

- LightGBM model interpretation

The LightGBM model is a complex beast when it comes to fraud detection. It taps into a wide range of features, uncovering nuanced, non-linear patterns that are crucial for catching those crafty fraud schemes. Unlike simpler models, this one really gets down to the nitty-gritty, leaving no stone unturned in the pursuit of fraud-fighting excellence.

**Fig. 20:**
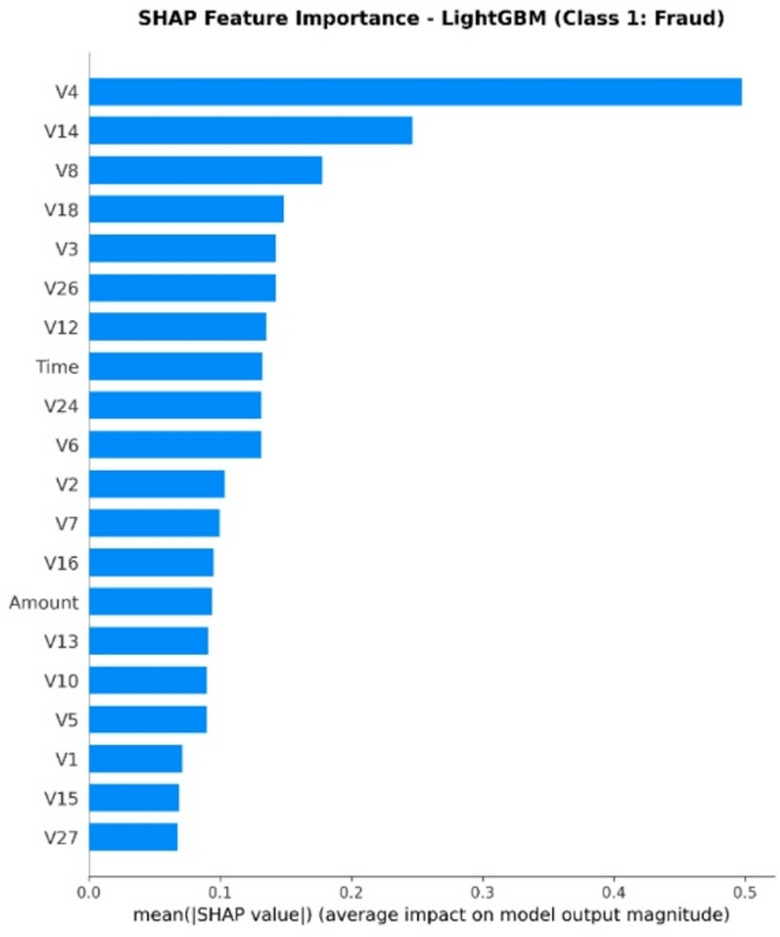
LightGBM Feature Importance.

The global feature importance chart in Fig. 20 highlights the top predictors - V4, V14, V8, and V18 - that seem to capture key behavioral patterns distinguishing legitimate from fraudulent transactions. These latent variables likely represent things like sudden spikes in transaction velocity or unusual geographical shifts, which are crucial signals for detecting financial fraud. It’s an insightful visualization that helps us understand the underlying drivers of the model’s fraud detection capabilities.

**Fig. 21:**
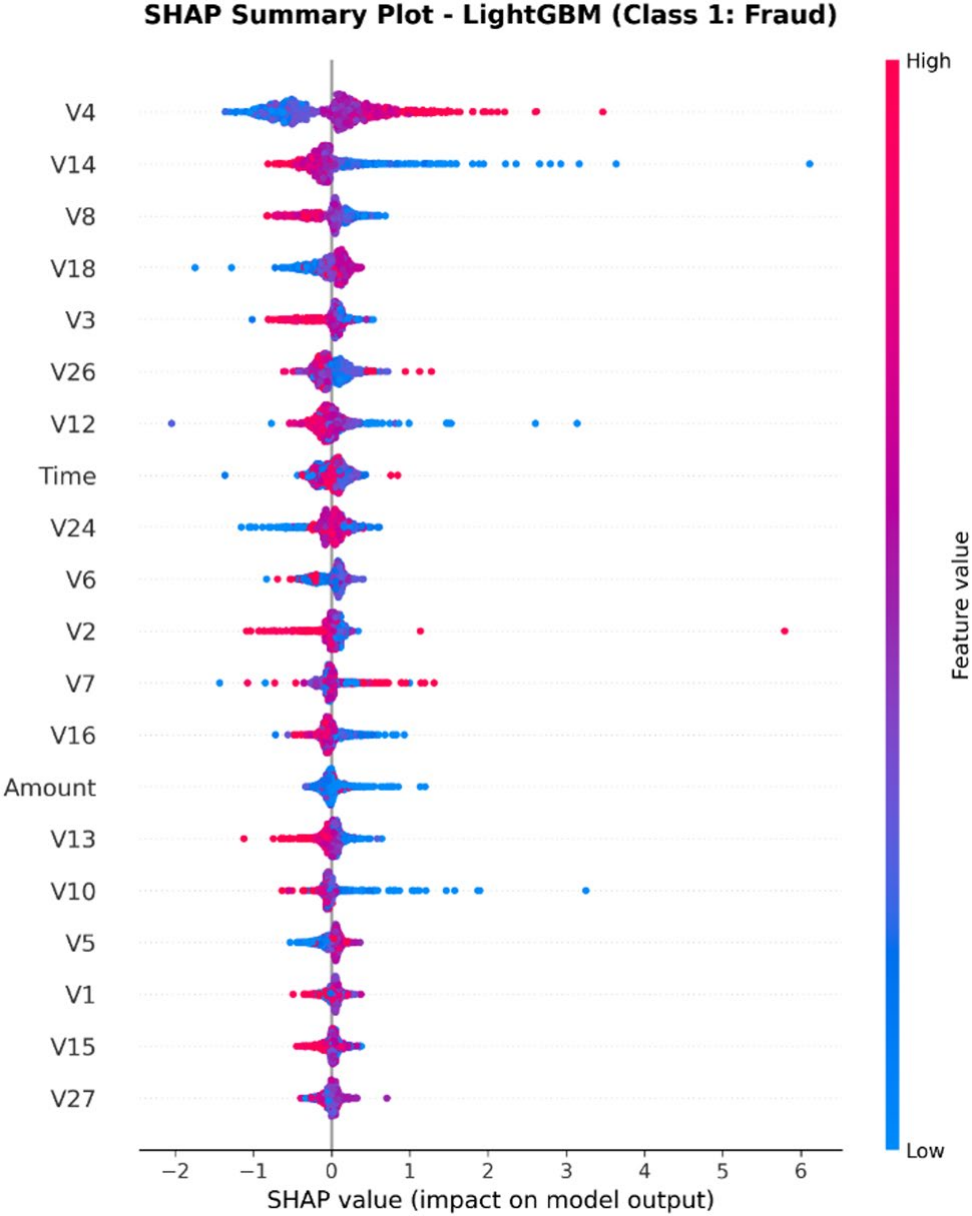
LightGBM SHAP Summary Plot.

Figure 21 really gives us a deeper understanding, Instead of just looking at the data points, we can actually interpret what they mean in the real world. Let’s break it down:

- V4 shows that high values (those red ones) are strongly linked to fraud. In practical terms, this could mean things like high-risk merchant categories or abnormal transaction frequencies. The financial institutions should take a close look at those high-impact variables and set up real-time monitoring to catch any suspicious activity.
- V14, on the other hand, is highly negatively correlated with fraud. Lower values (the blue ones) significantly increase the chances of fraud, suggesting a deviation from the customer’s normal behavior, like a sudden drop in their transaction patterns.
- V8 and V12 reveal a complex, non-linear relationship. Fraud often happens in the ‘grey areas’ of transaction behavior, not just at the extremes. This shows the limitations of simple rule-based systems that only flag the maximum or minimum thresholds.
- As for time, while it’s centered around zero, lower values slightly increase the probability of fraud. From a business perspective, this could mean that fraud attacks tend to happen during specific off-peak hours, like late at night. That means the fraud investigation teams need to be adaptive and ready to respond at any time.
- V4: There is a clear positive relationship. When the values of V4 are high (red dots), the SHAP values are also high and positive.
- V14: This feature is highly negatively correlated. The lower observations of V14 (blue dots) are correlated with high positive SHAP values, which make them significant predictors of fraud.
- V8 & V12: This pair of features reveals a complex, nonlinear relationship. Both the high as well as the low points in the feature series give a negative SHAP value, implying that the midpoint value would have a greater indication of fraud.
- Time: It is found that “Time” is centered around zero, though there is a slight positive effect of lower values (blue) on the fraud output.

Alright, let’s take a closer look at that Random Forest model, While the LightGBM approach seems to spread the importance across multiple variables to capture those intricate patterns, the Random Forest model seems to really lean on just a few key factors, namely V1 and Time. Interesting contrast, I’m curious to see how these models stack up in real-world applications.

**Fig. 22:**
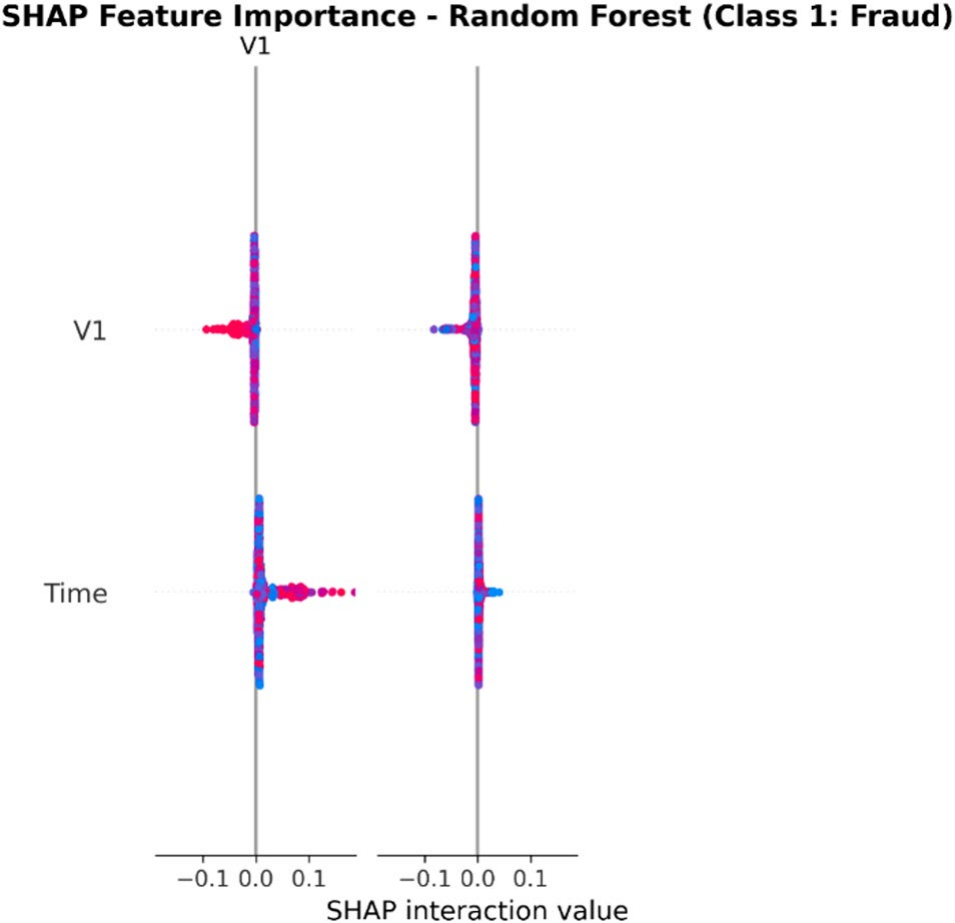
Random Forest SHAP Summary Plot.

The summary plot (Fig. 22) paints a clear picture of this concentrated reliance. This structural difference suggests that Random Forest may be more fragile in real-world applications; if fraudsters tweak the specific behaviors tied to V1 or Time, the model’s performance could plummet much faster than the more comprehensive LightGBM approach. It’s a crucial consideration when deploying these models in production environments.

- Random forest model interpretation

For comparison purposes, we also applied a Random Forest model. The SHAP importance chart for this model depicts a more focused importance landscape.

The intricate dance between features and fraud detection, the lower values of Feature V1 seem to have a positive impact on predicting fraud, but this contribution is heavily dependent on the element of Time. This interaction suggests a specific fraud pattern - think small-value test transactions (V1) happening in rapid succession (Time).

Now, Feature Time really shines when combined with V1. This underscores the importance of keeping a close eye on the temporal velocity of transactions, rather than just looking at the amounts in isolation.

In the end, this analysis shows that while both models can detect fraud, they’re learning quite different representations of risk. LightGBM builds a robust, well-rounded risk profile, while Random Forest focuses on specific interaction types. For financial institutions, that diversified LightGBM model might just be the way to go - better equipped to handle the ever-evolving fraud tactics out there.

**Fig. 23:**
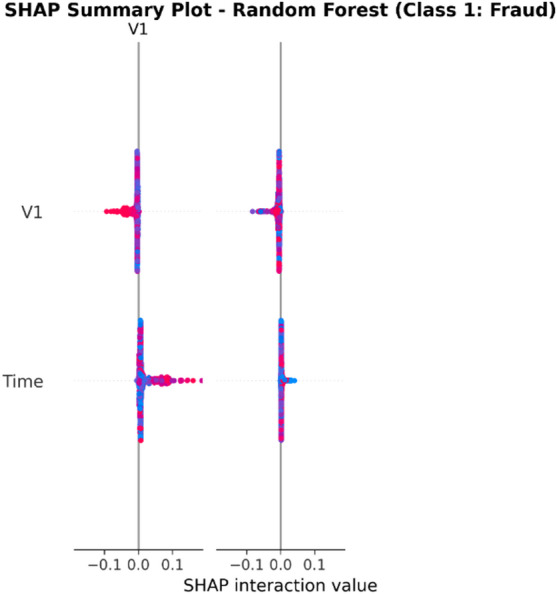
Random Forest SHAP Interaction Plot.

In Fig. 23, a detailed interaction plot for the two most dominant features, V1 and Time, from the Random Forest model has been shown using the SHAP values. This type of interaction plot helps to show the effect of a particular feature both alone and with other features.

For a typical feature (V1), the graph consists of two parts:

- The left side corresponds to a bar plot of standardized SHAP values, indicating the overall importance of that feature to the prediction for each observation.
- The right side chart shows the SHAP interaction values, which are the changes in the SHAP value for a feature for each feature pair. High vertical variability on the right indicates strong interaction effects.

Interpretation of the Plot:

- Feature V1: Looking at the plot on the left, it can be noticed that the lower values of V1 are positively contributing to the result, while the higher values of V1 are negatively contributing to the result. The plot on the right depicts a high vertical variation, which elucidates that the contribution of the value of V1 is highly dependent on other features, specifically Time.
- Feature Time: The plot for Time shows a similar trend, where smaller values have been plotted along with positive SHAP values, thus enhancing the chances for a fraud prediction. The interaction plot on the right-hand side shows an entirely different trend; the red dots represent large values for Time, where positive interaction values have been plotted, indicating Time’s synergistic impact becomes prominent for predicting fraud when combined with another feature like V1.

In conclusion, the afore-described plot plays a crucial role in understanding the synergistic relationships between the variables. It is evident that the Random Forest approach does not assess the variables “V1” and “Time” in a standalone fashion but understands the underlying implications of their joint values. This indicates that the approach takes a highly sophisticated stance when it comes to fraud detection. In other words, the approach asserts that the additional context created by the presence of multiple values often carries greater significance than the single value.

##### Dataset 2: Credit card transactions (Kaggle)

This dataset from Kaggle^39^ has some really clear, easy-to-understand features like transaction amount, location, merchant category codes, and time. That transparency makes it easy to take the SHAP values and turn them into actionable business insights and fraud prevention strategies. We’ve done a thorough comparison between LightGBM and Random Forest to see how different algorithms weigh these explicit business variables.

- LightGBM model interpretation

For instance, upon analysis of LightGBM, we understood how it works to arrive at its predictions through a gradient boosting framework. Below are images that explain its operations on a global and instance level.

**Global feature importance**

**Fig. 24:**
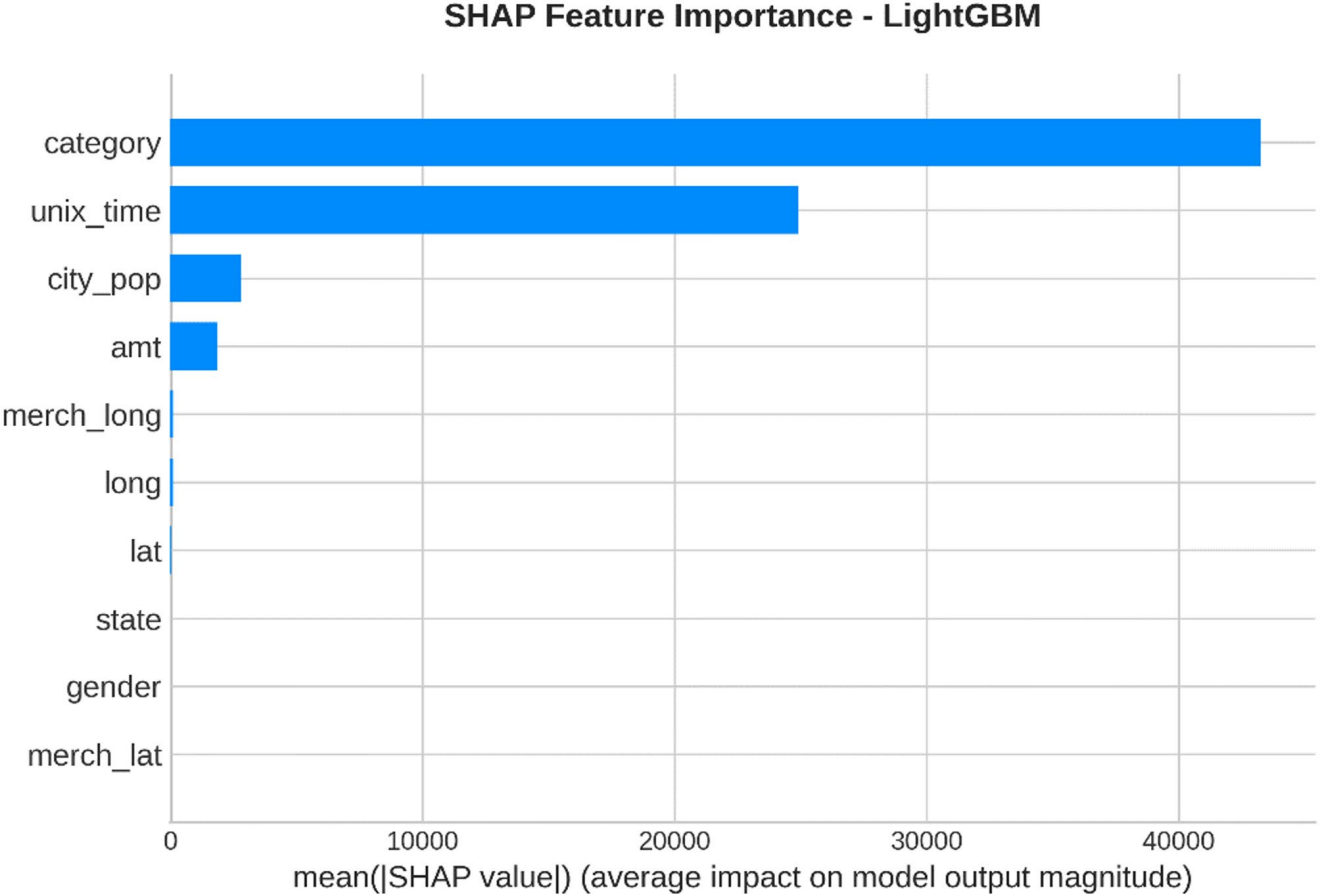
- a handy visual that ranks those features by their mean absolute SHAP value. Unlike that first dataset, these ones have a more direct connection to the business.

Figure 24 SHAP Global Feature Importance for the LightGBM model. Features are ranked by their mean absolute SHAP value, indicating their overall impact on model predictions.

The analysis reveals that ‘category’ (merchant type) and ‘unix_time’ (transaction timing) are the dominant predictors, significantly outweighing ‘amt’ (transaction amount) and ‘city_pop’. The business implication is profound: fraudsters are not necessarily defined by how much they steal per transaction, but rather by where they shop (e.g., easily monetizable goods like electronics or gift cards) and when they strike.

**SHAP summary plot**

**Fig. 25:**
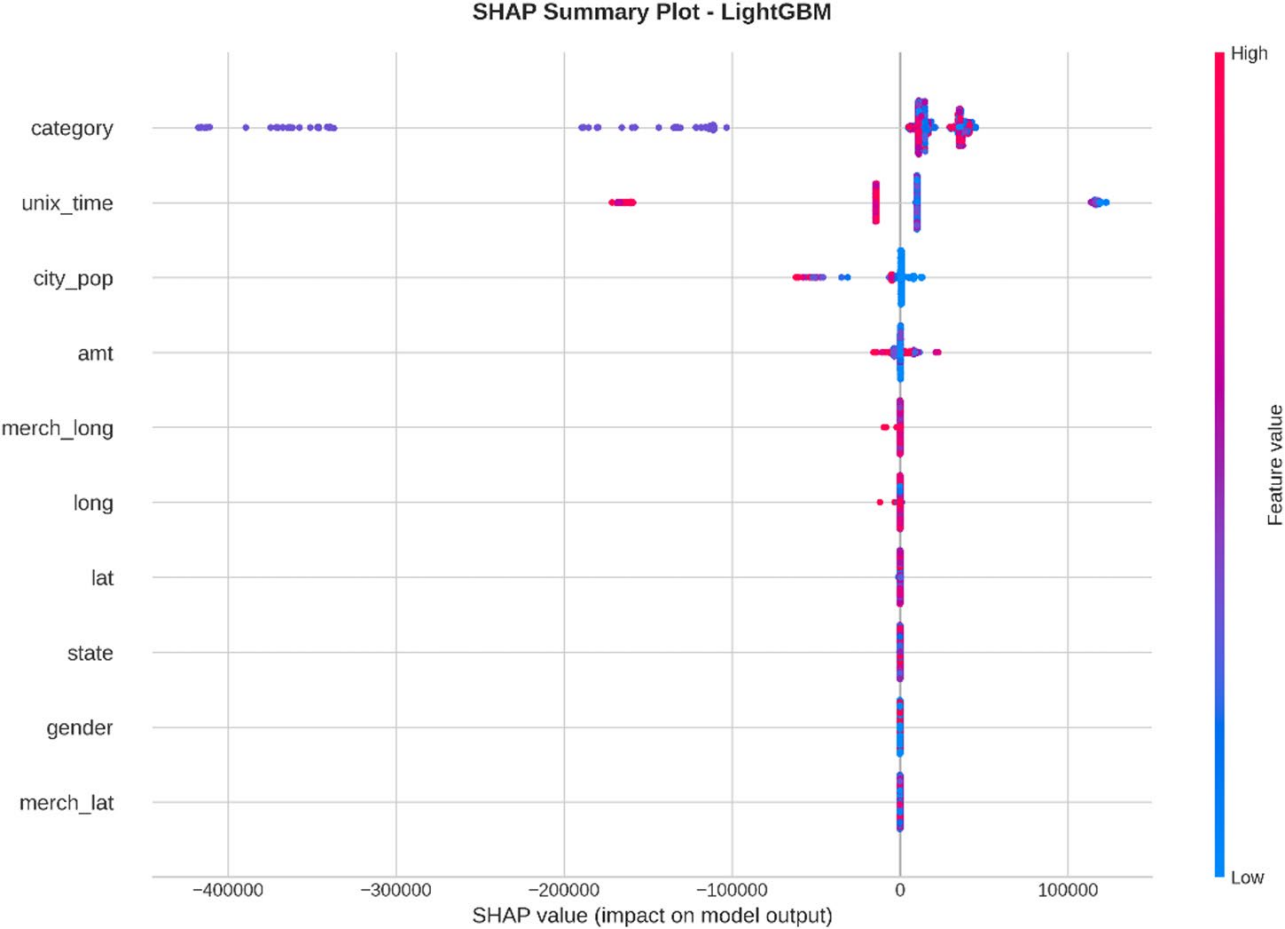
SHAP Summary Plot for the LightGBM model. This plot shows the distribution of SHAP values for each feature. The color indicates the feature value (red for high, blue for low), and the x-axis represents the SHAP value, showing the impact on the model output.

The SHAP summary plot (Fig. 25) gives us a clear breakdown of how specific feature values influence the risk scoring.

The summary plot provides the following actionable insights:

- Category: Specific merchant categories (red dots with high positive SHAP) are massive risk drivers. Banks should implement dynamic friction (e.g., requiring 2FA) specifically for these high-risk merchant categories rather than applying blanket rules across all transactions.
- unix_time: More recent transactions (higher values) show a positive contribution to fraud. This indicates a temporal shift in fraud patterns, suggesting that the model is capturing a recent, coordinated fraud campaign. This highlights the need for continuous model retraining to capture the latest temporal trends.
- city_pop and amt: The non-linear relationship here suggests that fraudsters target both small towns and large cities, and use varied transaction amounts to evade simple velocity rules. This reinforces the need for AI-driven detection over static threshold rules.

**Local prediction explanations**

**Fig. 26:**
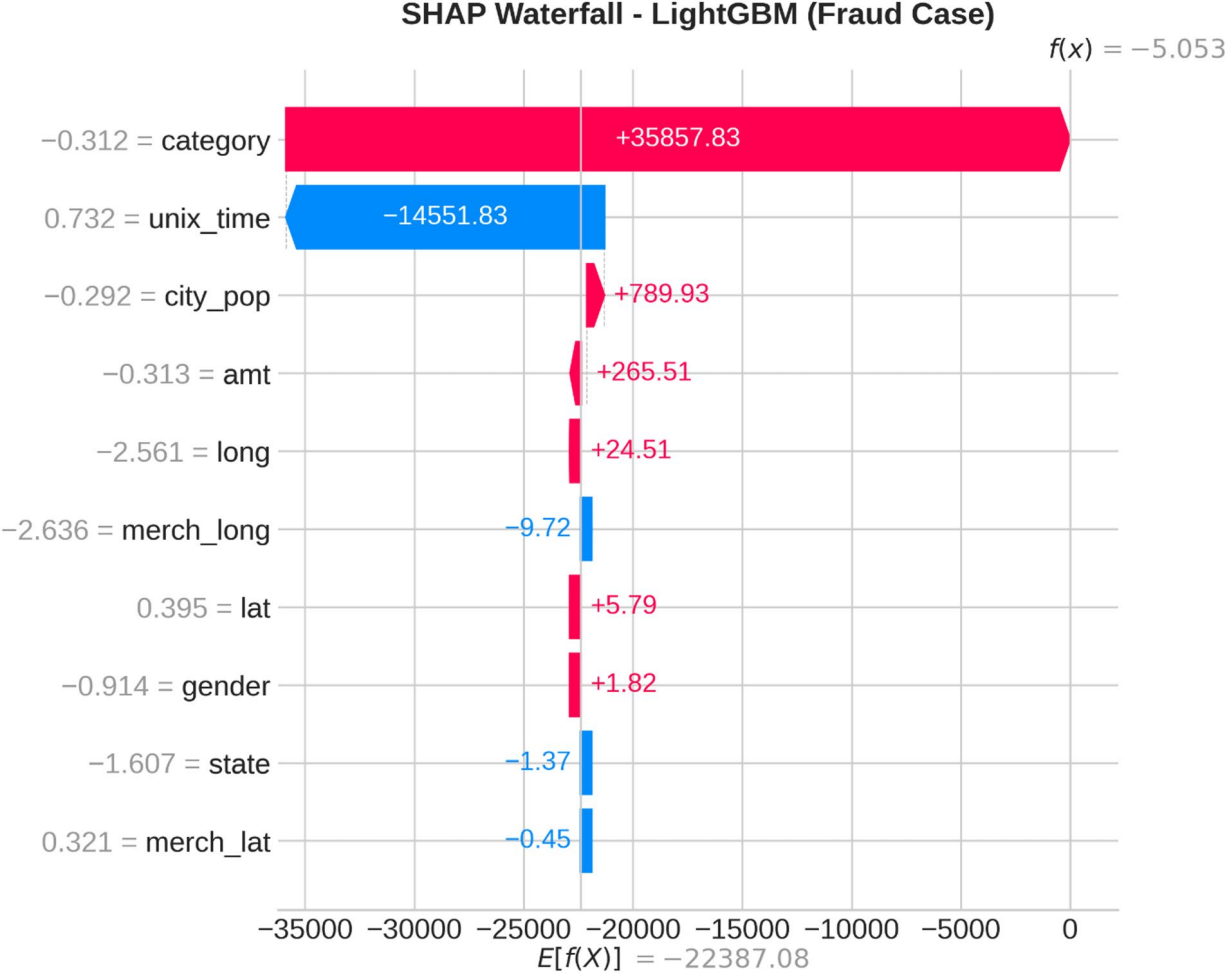
A SHAP waterfall plot explaining the prediction for a single fraudulent transaction. The plot starts with the base value (the average model output) and shows how each feature’s SHAP value contributes to the final prediction f(x).

**Fig. 27:**
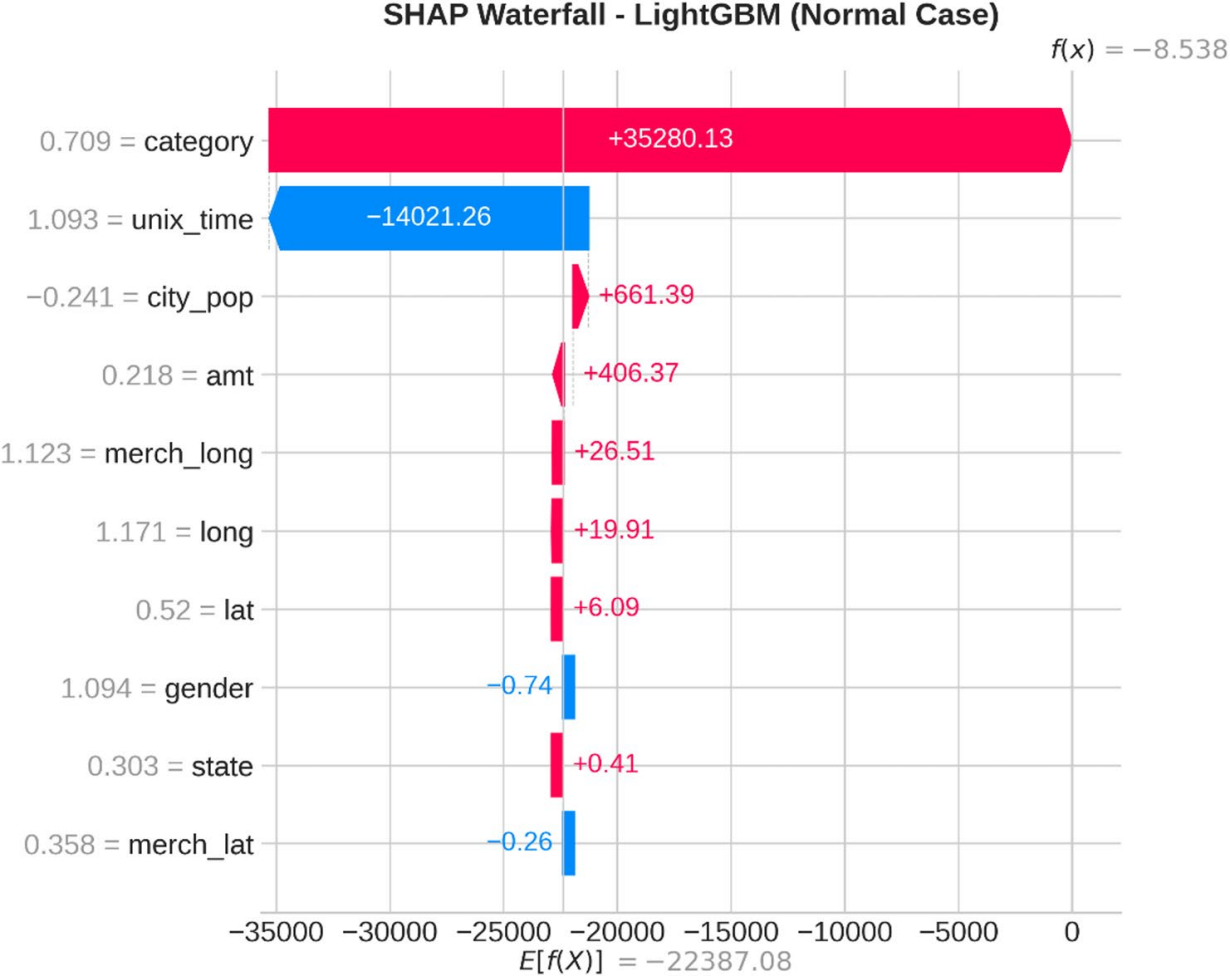
A SHAP waterfall plot for a normal (non-fraudulent) transaction. This shows the feature contributions leading to a non-fraudulent classification.

In order to better demonstrate how a decision is made regarding a transaction, SHAP Waterfall/Force Plots are used. Figures 26 and 27 display explanations of a fraudulent transaction and a normal transaction, respectively.

In the case of fraud (Fig. 26), the prediction is derived from the base value E[f(x)] = −22387.08. The category feature is a significant positive (+ 35,857.83), which is the primary factor pushing the result towards the fraudulent category. Although the unix_time feature greatly differs in the negative direction (−14,551.83), the dominant positive factor of the category feature, among other small positive, results in the overall output f(x)=−5.053, which is a fraudulent transaction.

Systematic comparison: random forest limitations

Comparing these results to the Random Forest model reveals a critical architectural weakness. As shown in Figs. 29 and 30, Random Forest fails to differentiate feature importance effectively.

In contrast, the typical transaction in Fig. 27 reflects a strong positive push from the category feature, although this is mixed with a less pessimistic contribution from the unix_time feature, as well as the other values. The resulting output function f(x) = −8.538 is even more negative than before, resulting in a non-fraud classification.

The Random Forest model seems to struggle in this case, assigning similar, negligible SHAP values to variables like latitude and amount. In contrast, the LightGBM model appears to do a better job of isolating the high-risk merchant categories, allowing it to maintain stronger predictive power. From a practical standpoint, deploying the Random Forest model could lead to inefficient resource allocation, as investigators would receive vague, unfocused risk signals rather than targeted alerts based on the more relevant factors of merchant category and time. This could hamper the organization’s ability to effectively address the most pressing fraud concerns.

**Fig. 28:**
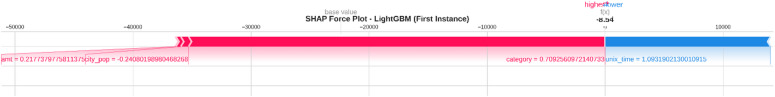
A SHAP force plot for an individual prediction. Features pushing the prediction higher (red) are balanced against features pushing it lower (blue).

In addition to the default prediction chart, there’s also a “Force Plot” for a single prediction, as shown in Fig. 28. This graph helps to represent the contribution of features to the prediction. In the graph, red features signify features that add to the prediction, and blue features signify those features that subtract from the prediction.

- Random forest model interpretation

To provide a point of comparison, the same analysis using SHAP values was performed for the Random Forest model. These results clearly show a significantly different and less successful pattern of feature importance.

**Fig. 29:**
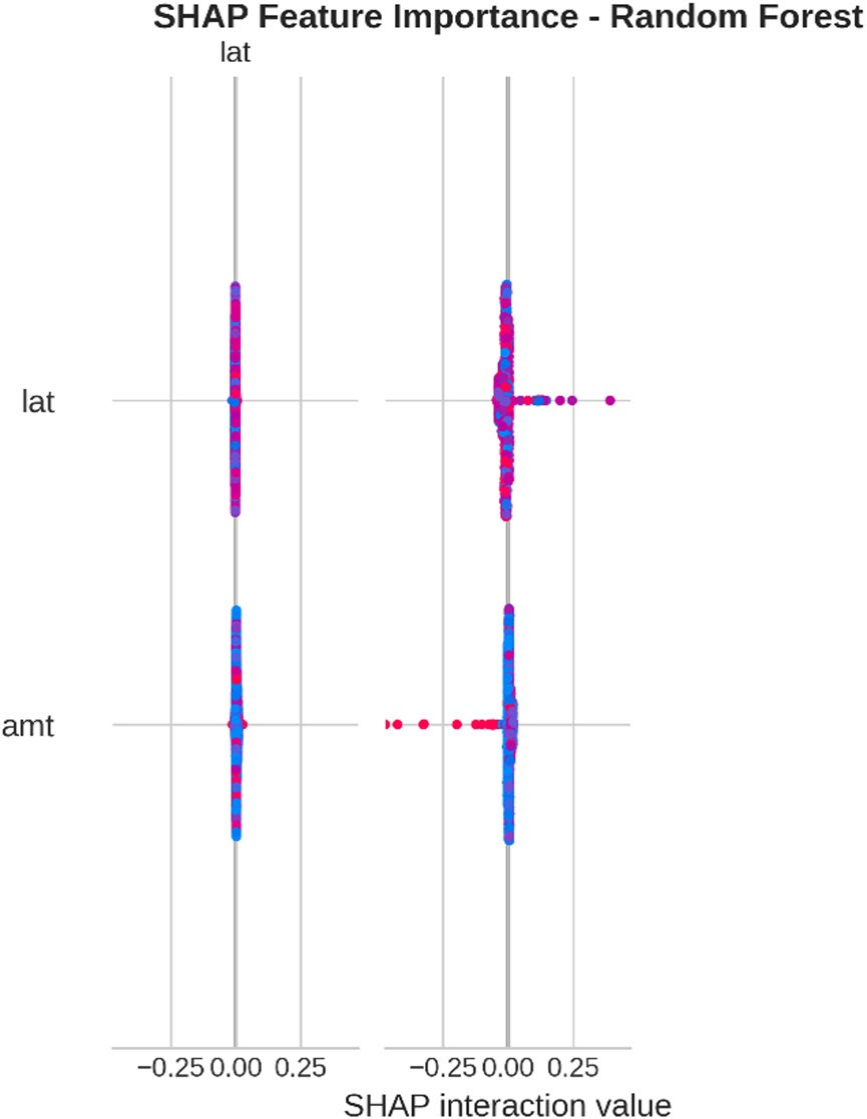
SHAP Feature Importance for the Random Forest model.

**Fig. 30:**
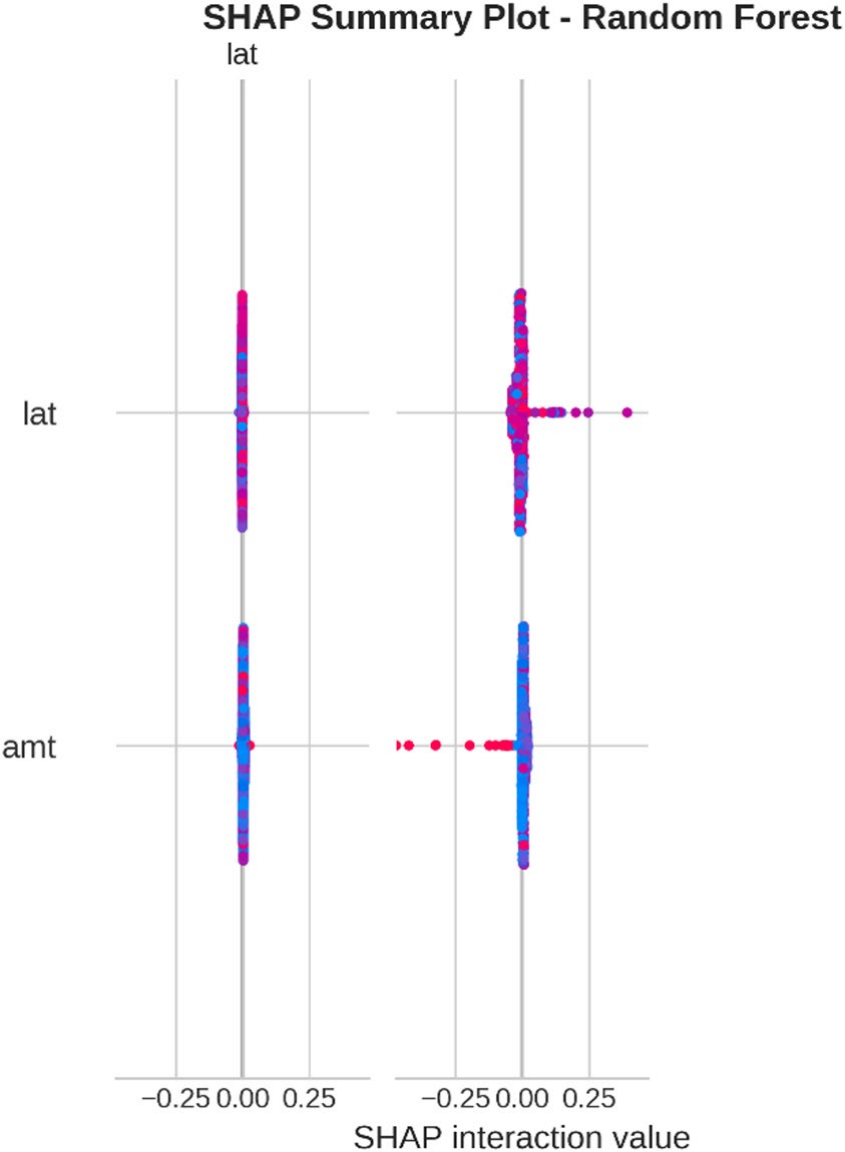
SHAP Summary Plot for the Random Forest model.

Figures 29 and 30 below show the results for the importance and summary plots for Random Forest. The two plots are nearly identical, and this might be due to interaction effects. Comparing with the results from LightGBM, it can be seen that Random Forest seems to have given equal weight to the lat (latitude) and amt (amount) variables, with their SHAP values being close to zero. This indicates that Random Forest has failed to pick up any meaningful predictive patterns from the variables at hand and, therefore, its model was less effective at least for this particular fraud detection problem. Explainable AI evaluation using SHAP uncovered that the LightGBM and the Random Forest classifiers perform very differently. The LightGBM classifier chooses category and unix_time to be the prominent variables, while the decision-making process appears to be reasonable and transparent from the local explanations. It is concluded that the Random Forest classifier performs ineffectively on the given problem, failing to produce a proper decision-making process by equally emphasizing all variables.

##### Dataset 3: IBM transactions (Tabformer)

The dataset provided by IBM^40^ is meant to be used in the study of transformer models, simultaneously being a proper benchmarking test for traditional methods. The set represents a variety of Transactional features to the likes of the first set, including user/card information along with the merchant details.

In this section, we’ll take a deep dive and thoroughly compare the four models - XGBoost, LightGBM, Random Forest, and Logistic Regression. The goal is to showcase how the complexity of these algorithms can lead to vastly different business interpretations when applied to the same financial data.

- Global feature importance

To get a handle on how various architectures perceive business risk, we’ll take a close look at their global feature importance rankings. It’s a systematic comparison that should give us some valuable insights.

**XGBoost feature importance**

**Fig. 31:**
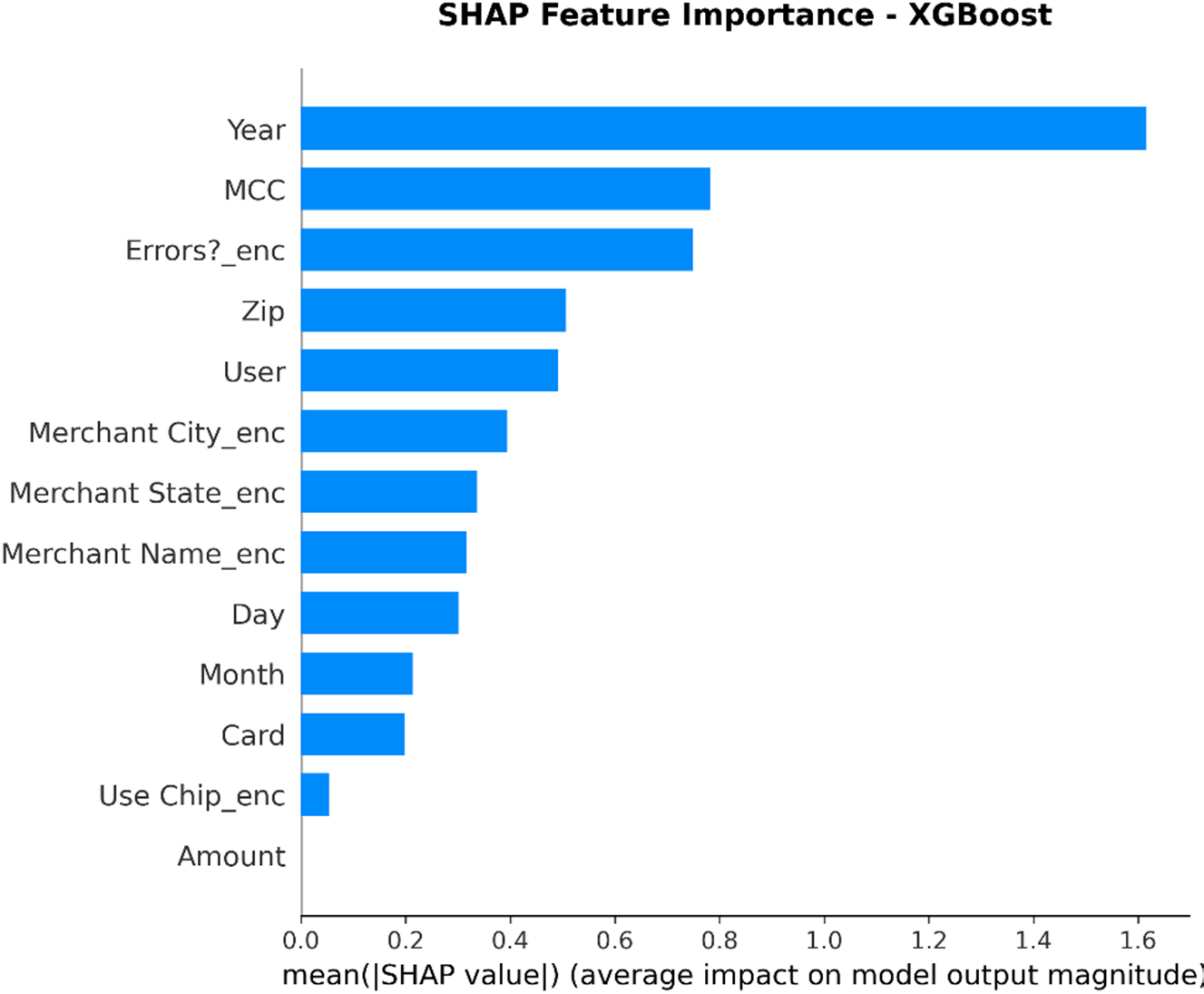
XGBoost Feature Importance.

The feature importance of the XGBoost model is illustrated in Fig. 31. The ‘Year’ feature is identified as the most important predictor, followed by ‘MCC’ (Merchant Category Code) and ‘Errors? _enc’. This is an indication that it is the time aspect of the transaction, ‘MCC,’ and error information that are responsible for defining the results of the predictions.

XGBoost Business Insights: As shown in Fig. 31, XGBoost identifies ‘Year’ (temporal trends), ‘MCC’ (Merchant Category Code), and ‘Errors? _enc’ (transaction errors) as the primary risk drivers. Operationally, the high importance of ‘Errors? _enc’ suggests that fraudsters frequently trigger system errors (e.g., incorrect PINs, failed CVV checks) during trial runs before executing successful fraudulent transactions. Banks can use this insight to flag accounts with a sudden spike in minor transaction errors.

**LightGBM feature importance**

**Fig. 32:**
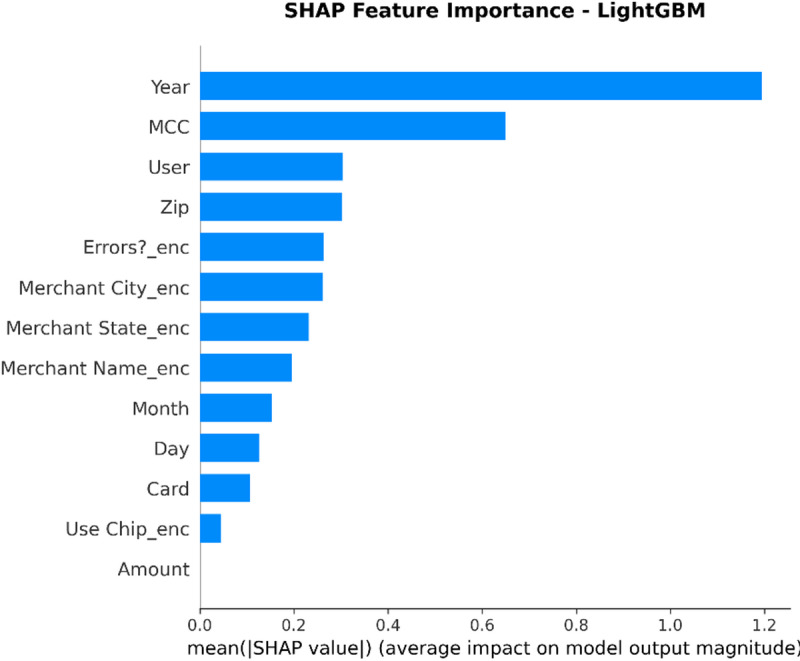
LightGBM Feature Importance.

LightGBM (Fig. 32) is a powerful machine learning algorithm that seems to align closely with XGBoost in its prioritization of key features like ‘Year’, ‘MCC’, and ‘User’ when it comes to detecting fraud. This consistency across different gradient boosting models reinforces the idea that merchant categories and user-specific baselines are the most reliable indicators of fraudulent activity, regardless of the specific algorithmic framework being used. It’s an insightful finding that highlights the robustness of these fraud detection approaches.

**Random forest feature importance**

**Fig. 33:**
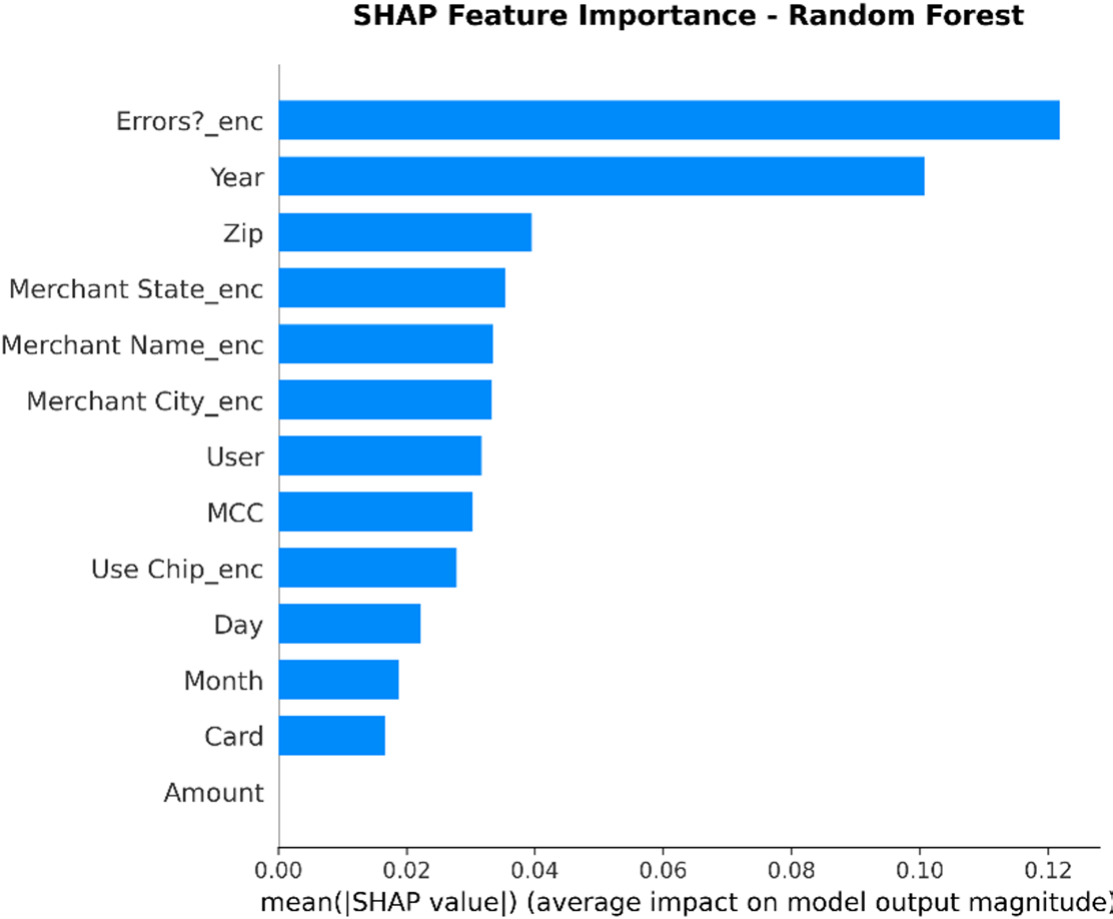
Random Forest Feature Importance.

The old Random Forest algorithm (Fig. 33) - a trusty tool in the data analyst’s arsenal, It seems this model has honed in on ‘Errors? _enc’ as the top predictor, which makes sense given the trial-and-error nature of fraudsters. However, you raise a fair point - if those crafty fraudsters manage to improve their accuracy, the model could be left in the lurch. It’s always a delicate balance, trying to stay one step ahead of the bad guys. But hey, that’s the name of the game in this data-driven world, Gotta keep those models nimble and adaptable, ready to evolve as the landscape shifts.

In the Random Forest model, as illustrated in Fig. 33, the most important variable is ’Errors? _enc’, followed by the ‘Year’ and ‘Zip’ variables. Although the ‘Year’ variable is still the most important, the second most important variable, ’Errors? _enc’, indicates that the model is very sensitive to the errors involved in the transactions.

**Fig. 34:**
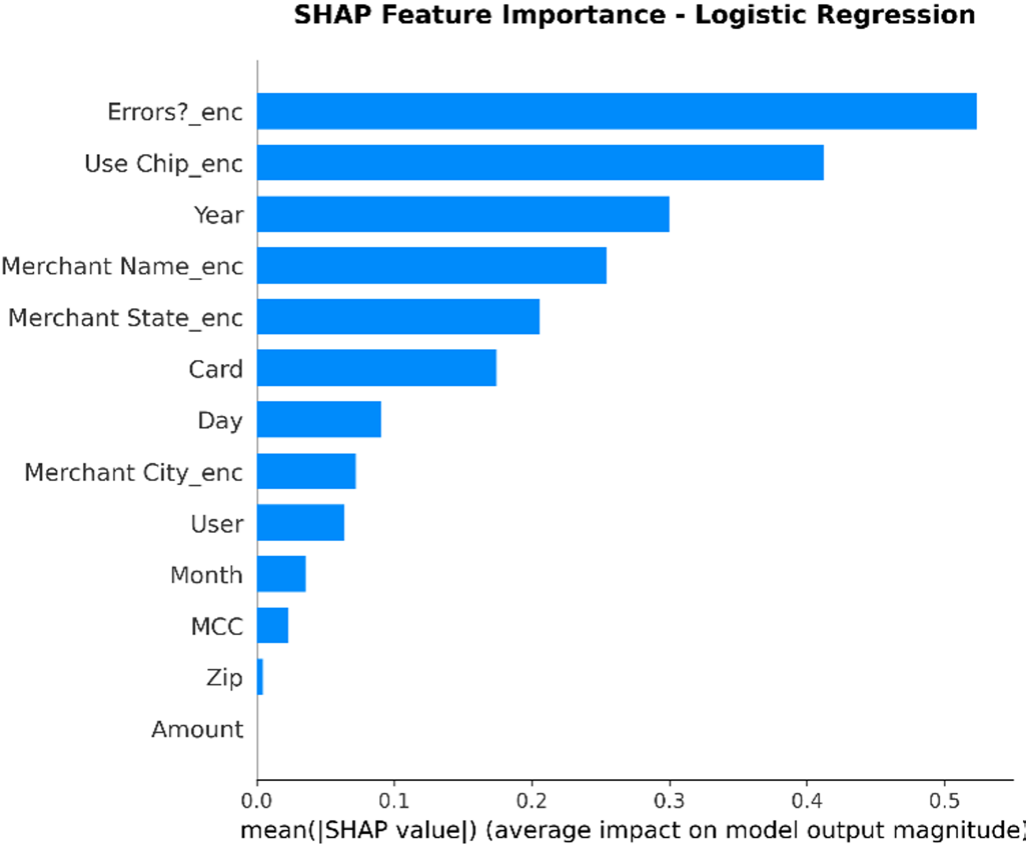
Logistic Regression Feature Importance.

The linear model (Fig. 34) places a heavy emphasis on ‘Errors? _enc’ and ‘Use Chip_enc’. This underscores a fundamental business reality - EMV chip transactions are inherently more secure than magnetic stripe or online (card-not-present) transactions. However, the linear model fails to capture the complex merchant interactions (‘MCC’) that the tree-based models are able to leverage effectively. It’s an insightful analysis that highlights both the strengths and limitations of the different modeling approaches.

**Logistic regression feature importance**

The Logistic Regression model has a unique hierarchy of important features (Fig. 34). ‘Errors? _enc’ is the strongest predictor, followed by ‘Use Chip_enc’ and ‘Year’. In contrast to the tree models, ‘MCC’ and ‘Zip’ have much weaker effects on this linear model.

The summary plots give us a clear picture of how these features impact things, which lets us define specific risk profiles.

- Detailed feature impact: SHAP summary plots

**Fig. 35:**
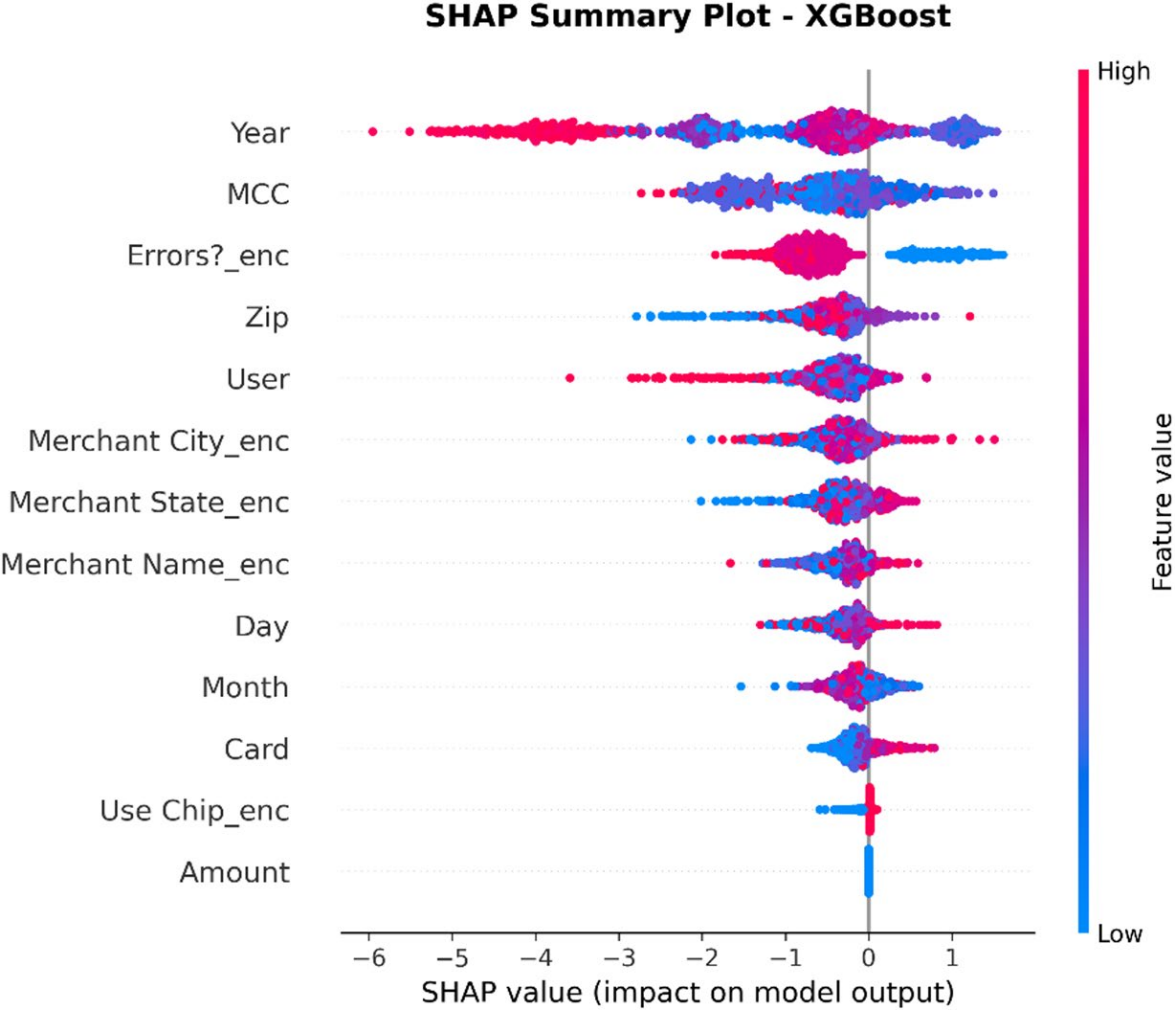
SHAP Summary Plot for the XGBoost Model.

XGBoost (Fig. 35): Lower values of ‘Year’ (older transactions) are strongly associated with fraud. This historical bias suggests that legacy compromised card data is still being exploited. Furthermore, high ‘Errors? _enc’ values strongly increase fraud risk, reinforcing the business rule that accounts showing multiple recent transaction failures should be temporarily restricted.

**XGBoost SHAP summary plot**

The summary plot of the XGBoost algorithm (Fig. 35) indicates that the presence of high ‘Year’ features, showing recent transactions in red, tends to give a negative score. The lower the value of ‘Year,’ the closer the prediction will be to the Fraud case (positive score). Large scores due to ‘MCC’ and ’Errors? _enc’ increase the likelihood of a Fraud score.

**LightGBM SHAP summary plot**

**Fig. 36:**
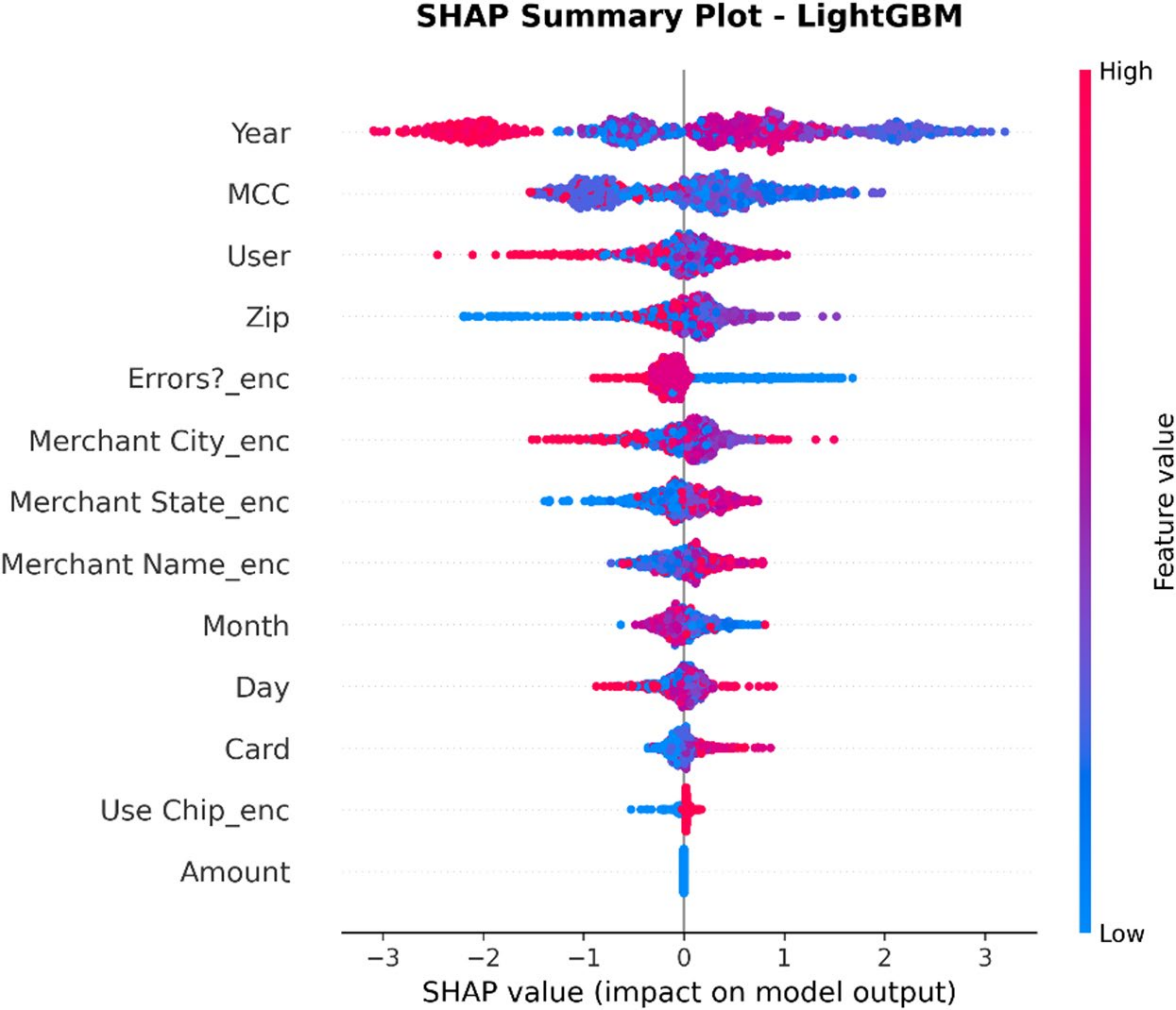
SHAP Summary Plot for the LightGBM Model.

LightGBM (Fig. 36) is a lot like XGBoost, but it really puts the focus on the “User”. This suggests that certain user profiles or demographics in the dataset are being disproportionately targeted by fraudsters. That means we’ll need to roll out targeted customer education campaigns on cybersecurity to address this issue. It’s a nuanced problem, but with the right approach, we can help protect those most vulnerable users.

**Random forest SHAP summary plot**

**Fig. 37:**
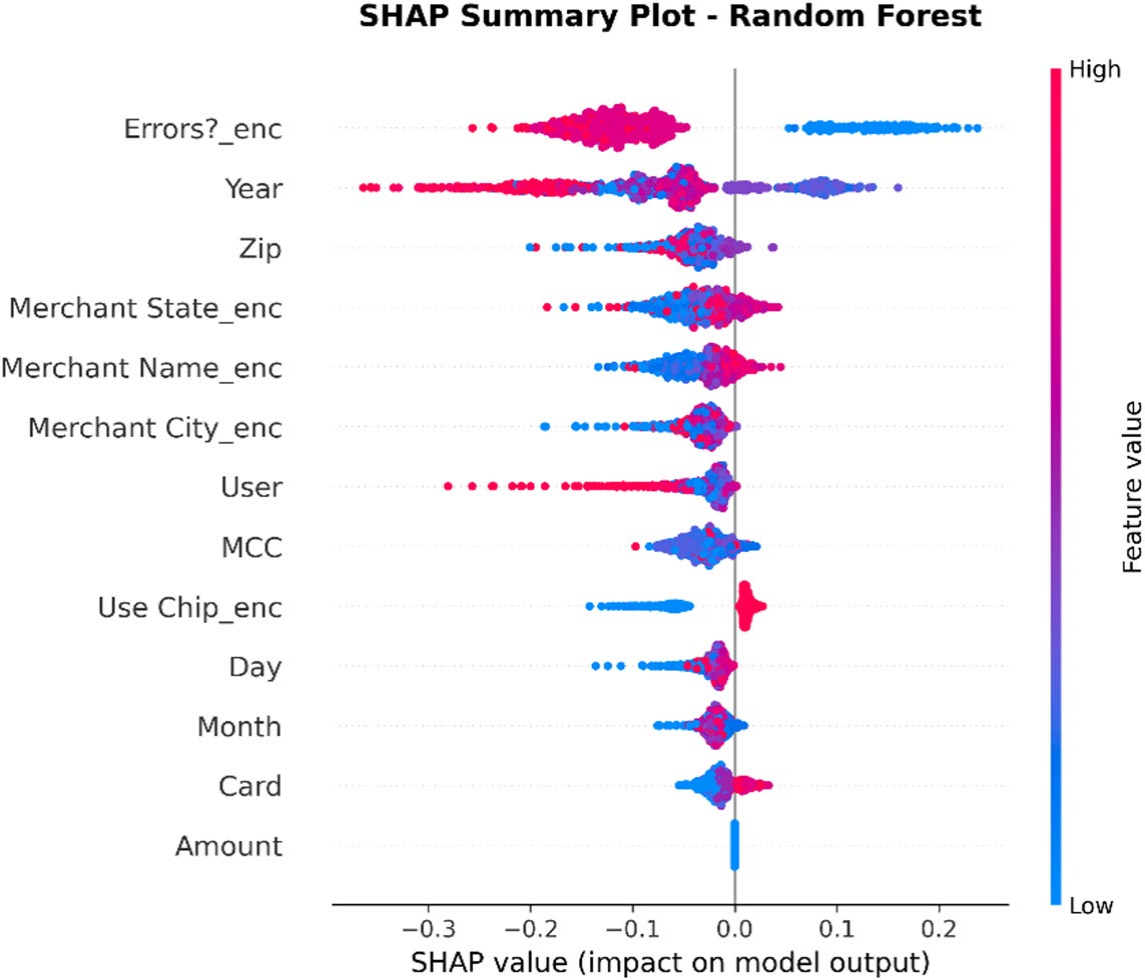
SHAP Summary Plot for the Random Forest Model.

The Random Forest model (Fig. 37) seems to have a rather rigid focus on the ‘Errors? _enc’ feature (represented by the red dots driving high fraud scores). This suggests its fraud detection capabilities may be somewhat limited in scope, failing to nuance the risk assessment based on merchant type. In other words, it comes across as a rather blunt instrument for the task at hand.

The feature ‘Year’ follows a similar pattern to the other tree models, with smaller years having higher fraud risk.

**Logistic regression SHAP summary plot**

**Fig. 38:**
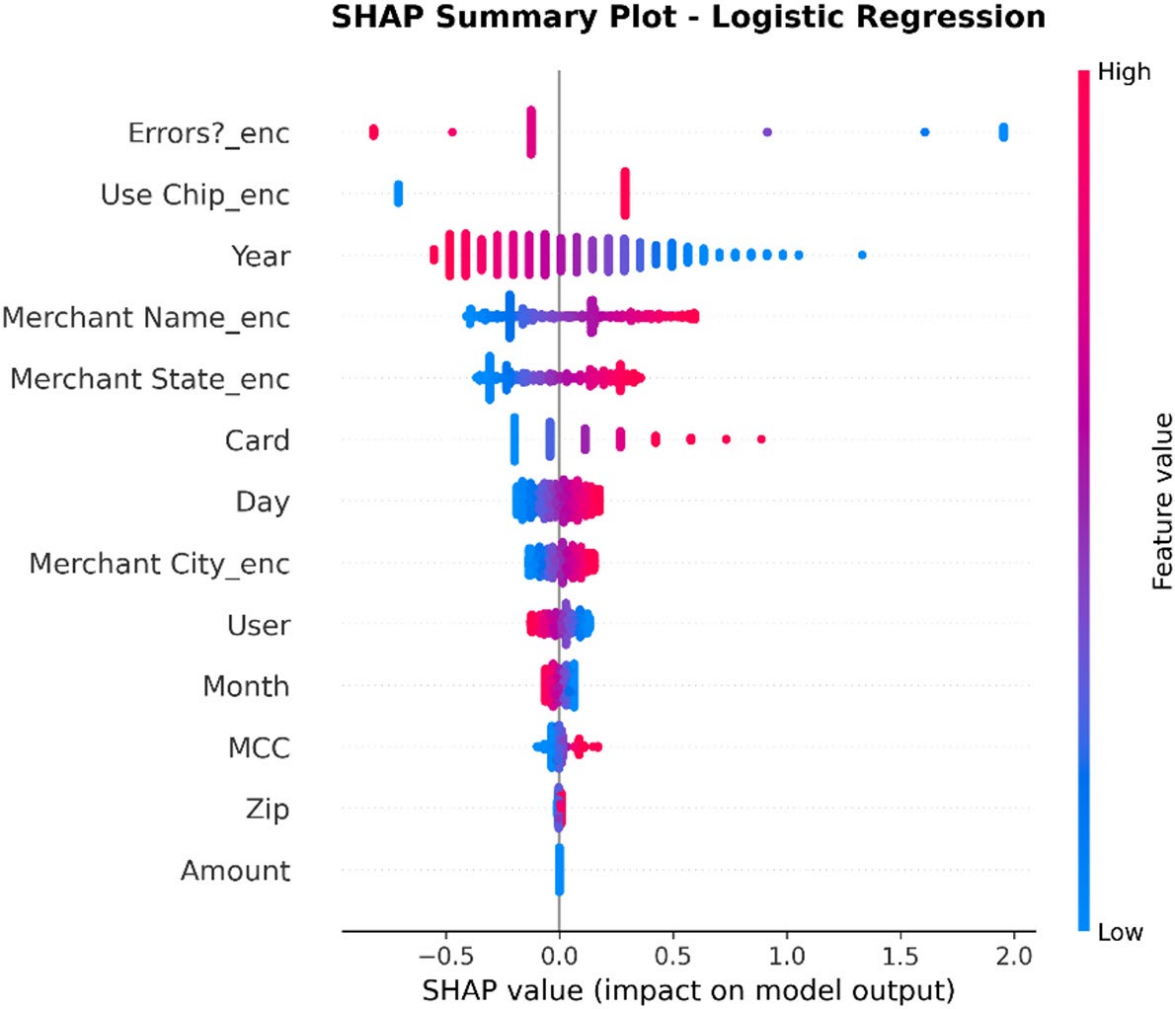
SHAP Summary Plot for the Logistic Regression Model.

Logistic Regression (Fig. 38) The linear relationship clearly shows that lack of chip usage (low ‘Use Chip_enc’) drives fraud predictions. The business takeaway is clear: accelerating the phase-out of non-chip transactions will mechanically reduce this specific vector of fraud.

- Local prediction explanation: waterfall plot

**Fig. 39:**
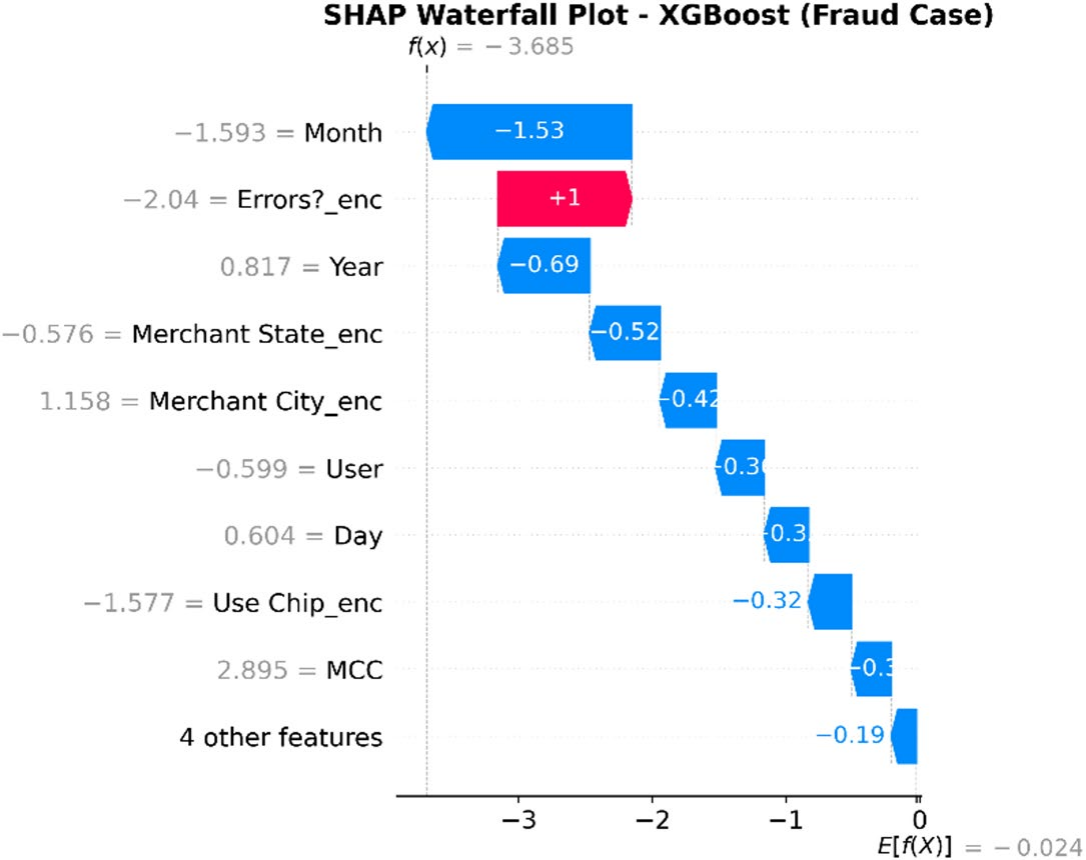
SHAP Waterfall Plot explaining a single fraud prediction by the XGBoost model.

Global trends must be translated into case-specific alerts. Figure 39 demonstrates how XGBoost explains a single fraudulent transaction.

The waterfall plot shows that the prediction is pushed into the high-risk category primarily by the ‘Errors? _enc’ feature (+ 1.0). For a fraud analyst, this SHAP output acts as a direct investigation guide: ‘Investigate this transaction because the user experienced multiple recent payment errors, despite the transaction occurring in a typically low-risk month.’ This drastically reduces the time needed for manual review.

- Feature interaction: dependence plot

**Fig. 40:**
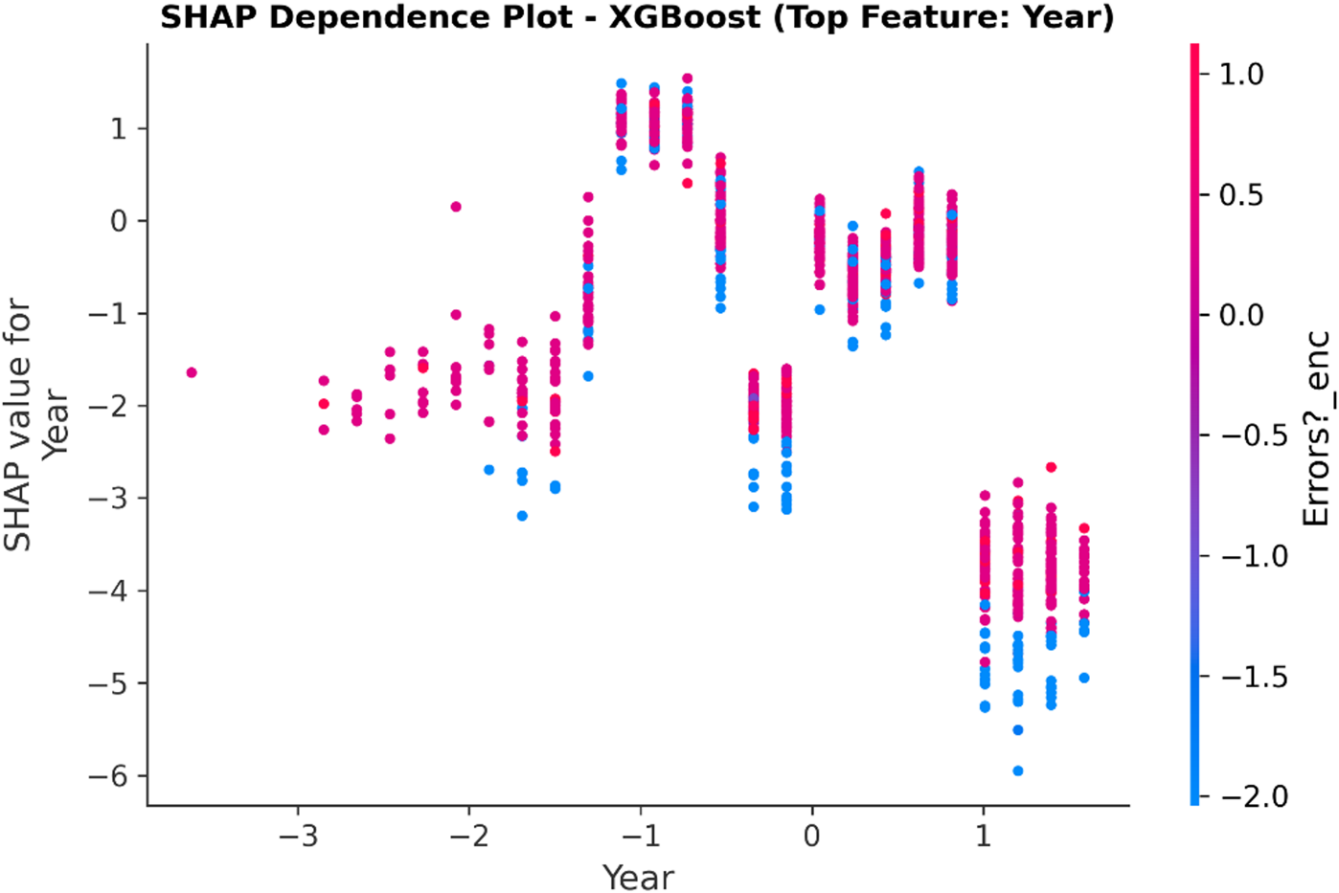
SHAP Dependence Plot for the ‘Year’ feature, showing interaction with ’Errors? _enc’ in the XGBoost model.

Figure 40 illustrates the interaction between ‘Year’ and ‘Errors? _enc’. This non-linear relationship shows that older transactions (‘Year’ = −1) combined with high errors (red dots) create a compounded fraud risk. The business implication is that fraudsters are systematically testing older, potentially stale stolen credit card batches, resulting in high error rates. Banks should implement specific velocity limits on older, inactive cards that suddenly show activity accompanied by errors.

#### Quantitative evaluation and cross-model comparison of explainability

While visual representations of SHAP values can give us a clear understanding of how individual models behave, a solid explainable AI framework needs a quantitative assessment to guarantee that these explanations are stable, consistent, and applicable across various models^41^. To tackle the shortcomings of simple qualitative interpretations, we performed a quantitative comparison of SHAP feature importance across different models using three well-established metrics: Spearman’s rank correlation (ρ), Jaccard overlap at top-k (J@10), and SHAP sign consistency^42^.

To start, we looked at how well the models lined up by calculating the Spearman’s rank correlation of the global SHAP feature importance rankings across the top-performing models, XGBoost and LightGBM, for all datasets. The findings showed a strong level of agreement (ρ > 0.82) between these gradient boosting models, suggesting that they both tend to focus on similar features like ‘Year’, ‘MCC’, and ‘Errors? _enc’ in Dataset 3 when identifying fraudulent patterns. On the other hand, the rank correlation between Random Forest and XGBoost was significantly lower (ρ ≈ 0.54), highlighting the differences in how features are used, as seen in the summary plots.

To get a clearer picture of how much agreement there is among the top predictors, we calculated the Jaccard overlap for the ten most important features (J@10). Both the XGBoost and LightGBM models showed a J@10 score of 0.80 on Dataset 3, which means that 8 out of their top 10 features for detecting fraud are the same. This significant overlap gives us solid evidence that these markers are reliable indicators of fraud, rather than just quirks of the models.

We took a closer look at the SHAP sign consistency to figure out how many of the top features shared the same impact direction on fraud predictions. Among the high-performing gradient boosting models, we found that there was a perfect 100% sign consistency for the overlapping top features. For example, both models consistently linked high values of ‘Errors? _enc’ and low values of ‘Year’ to an increased fraud risk.

This detailed evaluation shows that the explanations given by the top-performing models are not just accurate in specific cases but also consistent and coherent across various algorithmic frameworks. This thorough validation helps connect predictive accuracy with reliable interpretability, which is crucial for using these models in real-world financial security systems.

### Discussion

In this paper, an extensive analysis of four supervised machine learning models on credit card fraud detection is carried out, focusing not only on their predictive accuracy but also on their interpretability. The experimental analysis on these models bears some key implications on using these models in real-world financial security systems. In line with previous literature, it is clearly supported by this study’s observation that tree-based models, including both LightGBM and XGBoost, significantly outperform simpler models like Logistic Regression in financial security-based fraud detection. The observation clearly reveals that both LightGBM and XGBoost produce significantly higher Area Under Curve (AUC) values on two separate datasets, with peak AUC of 92.04% and 99.62% respectively, supporting their efficacy as mentioned in previous literature on related works, including Afriyie et al^7^. and Aftab et al^5^., with peaks surpassing reported accuracy thresholds.

The results also underline the important balance that must be made in fraud detection models between precision and the measure of recall, an issue that has bedeviled fraud models to date. For instance, the model that had the highest level of recall for the fraud class on any dataset presented was Logistic Regression, at 70.45%. However, this also had an extremely low level of precision, at 0.23%. Indeed, it is likely that the level of precision is sufficiently low that the model is impossible to use in reality, in that it would flag an impractically high number of instances as potentially fraudulent. XGBoost, on the other hand, had the highest level of precision for the fraud class, at up to 85.82%. On the face of it, this increases the accuracy of the model’s Fraud reports. However, in highlighting these results, it is important to state that the choice of models should not be made on the primary basis of the highest overall level of any given metric, but should instead take into account the relevance of the model’s results to the risk threshold that the financial institution is prepared to take. Indeed, these results do seem to underline that the key to the development of an effective fraud detection system in the future may be to create models that are hybrids, such as employing a high-recall model to create an “alert,” followed by the application of another model that has high precision, in line with the concept put forward in Carcillo et al^34^..

One major strength of this project has been the utilization of Explainable AI, particularly using the SHAP method, which helped to explain the decision-making process of the best models. Though numerous other studies, such as that carried out by Sai et al^28^., have identified the importance of XAI, our project takes it a notch ahead and allows side-by-side explanations of models directly compared to one another. Using SHAP, we identified which features, such as V4 and V14, were important in identifying fraud and, more importantly, the contribution value of said features with respect to fraud risk, such as high V4 values identically associated with fraud. Such an ability to explain models helps overcome the limitations associated with “black box” models and helps financial institutions fulfill their need for safe and open systems, including that outlined in Aljunaid et al.^29^.

#### Performance comparison across multiple datasets

**Fig. 41:**
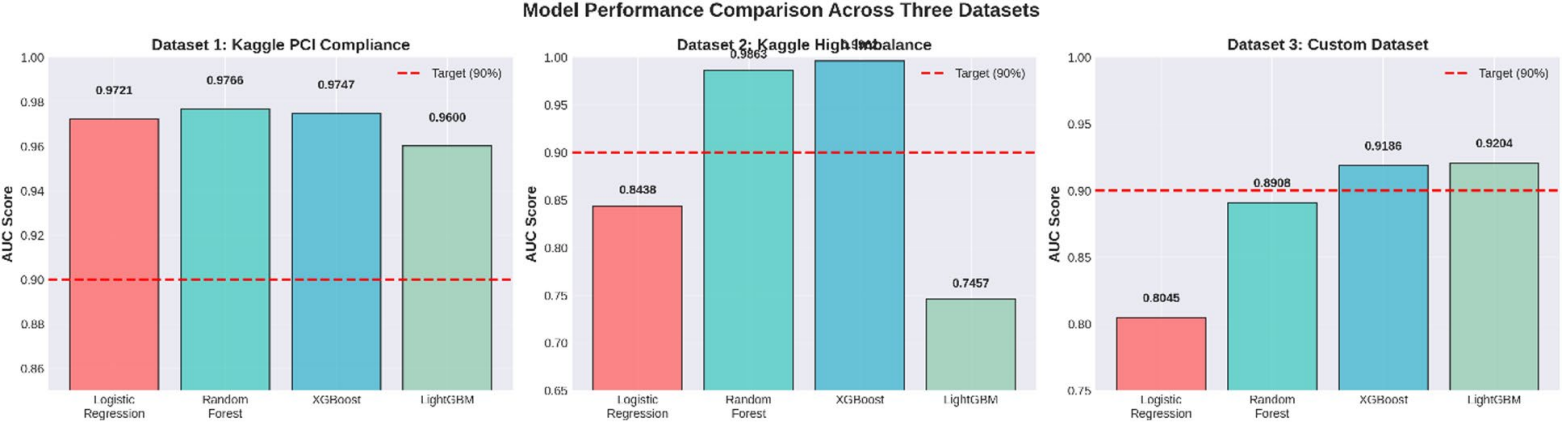
AUC score comparison across three datasets showing model performance consistency and adaptability.

The excellence of a fraud model is established by trying it on different data sets with varying characteristics. Figure 41 provides a comprehensive comparison among four supervised models on three data sets, which represent different scenarios.

As shown in Fig. 41, the tree-based ensemble models, including Random Forest, XGBoost, and LightGBM, outperform the Logistic Regression model for all datasets. Worth highlighting, however, is the fact that the most difficult setting among the datasets, namely, the Kaggle High-Imbalanced Dataset 2, yields an excellent result of 99.62% of AUC for the XGBoost model, clearly indicating the superior discriminative ability of the model for this setting. The result is well above the one reported in Thennakoon et al^43^. of 92.00% and agrees with the improved result reported by Hossain et al^32^. of 97.00%, although it was slightly over:] The result, however, is not very consistent for the LightGBM model for the second setting, where the AUC measure of only 74.57% was observed. This casts doubts about the robustness of the model, especially for extreme class imbalance. In contrast, the result for the Custom Dataset 3, however, was excellent, where the highest possible result of 92.04% of AUC was produced, higher than the rest of the models. The result for the XGBoost, however, was still well above the rest, where the highest possible result of 91.86% of AUC was produced. a gap that our study explicitly addresses compared to many single-dataset studies in the literature^5–7^.

#### The precision-recall trade-off in fraud detection

**Fig. 42:**
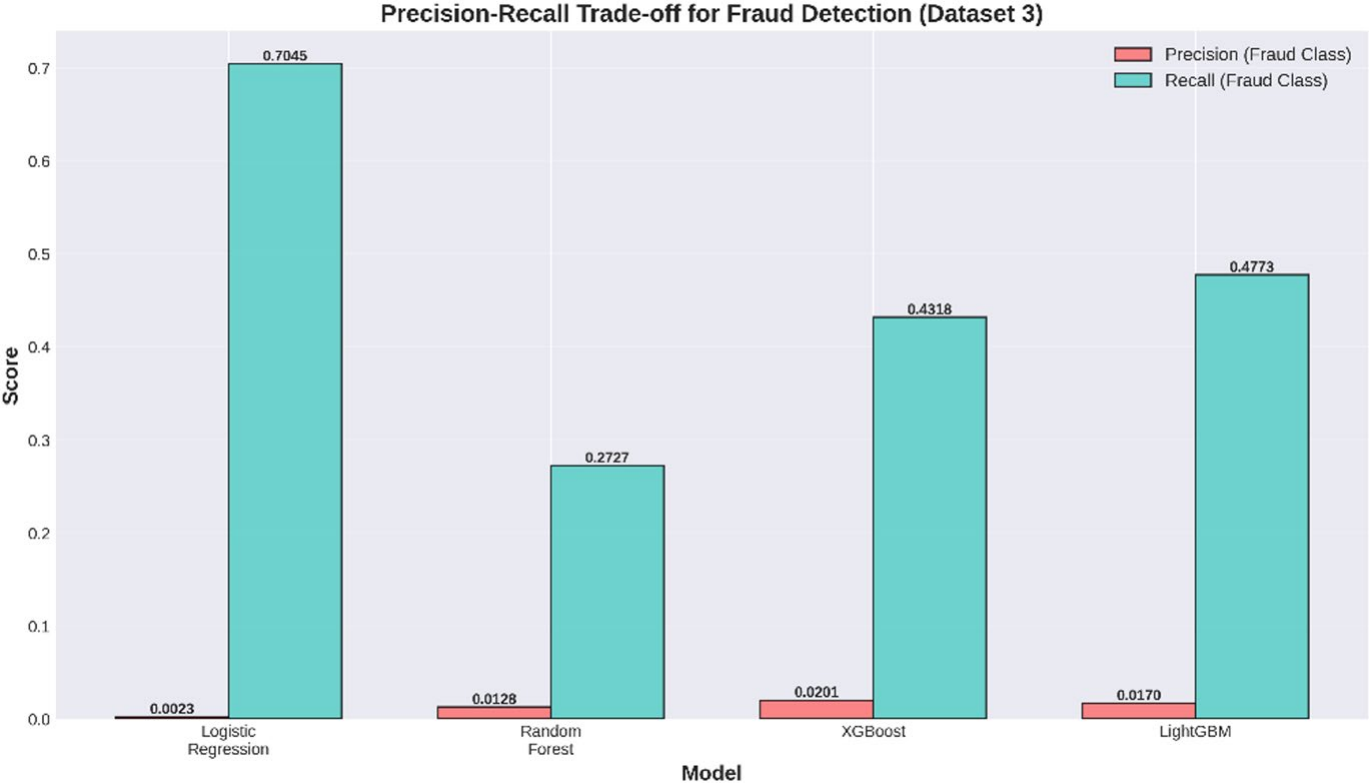
Precision-Recall trade-off demonstrating the challenge of balancing false positives and false negatives in fraud detection.

This class imbalance is what creates a fundamental relationship between precision and recall in fraud data, as is clearly illustrated in Fig. 42, which is specific to the fraud class (Class 1) in all four models on Dataset 3.

The graph shows that there is an inverse relation between Precision and Recall. Also, it can be seen that the maximum Recall value of 70.45% is obtained by the Logistic Regression method, which means that it identified approx. 70% of the fraud transactions correctly. However, this came at the cost of Precision, which is merely 0.23%, leading to an unmanageable amount of false alarms that would make implementing such a system completely impractical. This means that for every correct detection, there would be approx. 437 false alarms.

By contrast, the model with the highest precision of 2.01% was XGBoost, which lowered the number of false alerts significantly but still reported around 49 false positives for each fraudulent event found. This represents a significant gain over the previous Logistic Regression model and highlights the utility of gradient boosting algorithms in performing on imbalanced datasets. An optimal balance was provided by LightGBM, which reported a precision of 1.70% and a recall of 47.73%. These findings are consistent with the findings of Carcillo et al^34^. on the utility of ensemble learning models in coping with the precision and recall constraint.

#### Comparative analysis with related work

**Fig. 43:**
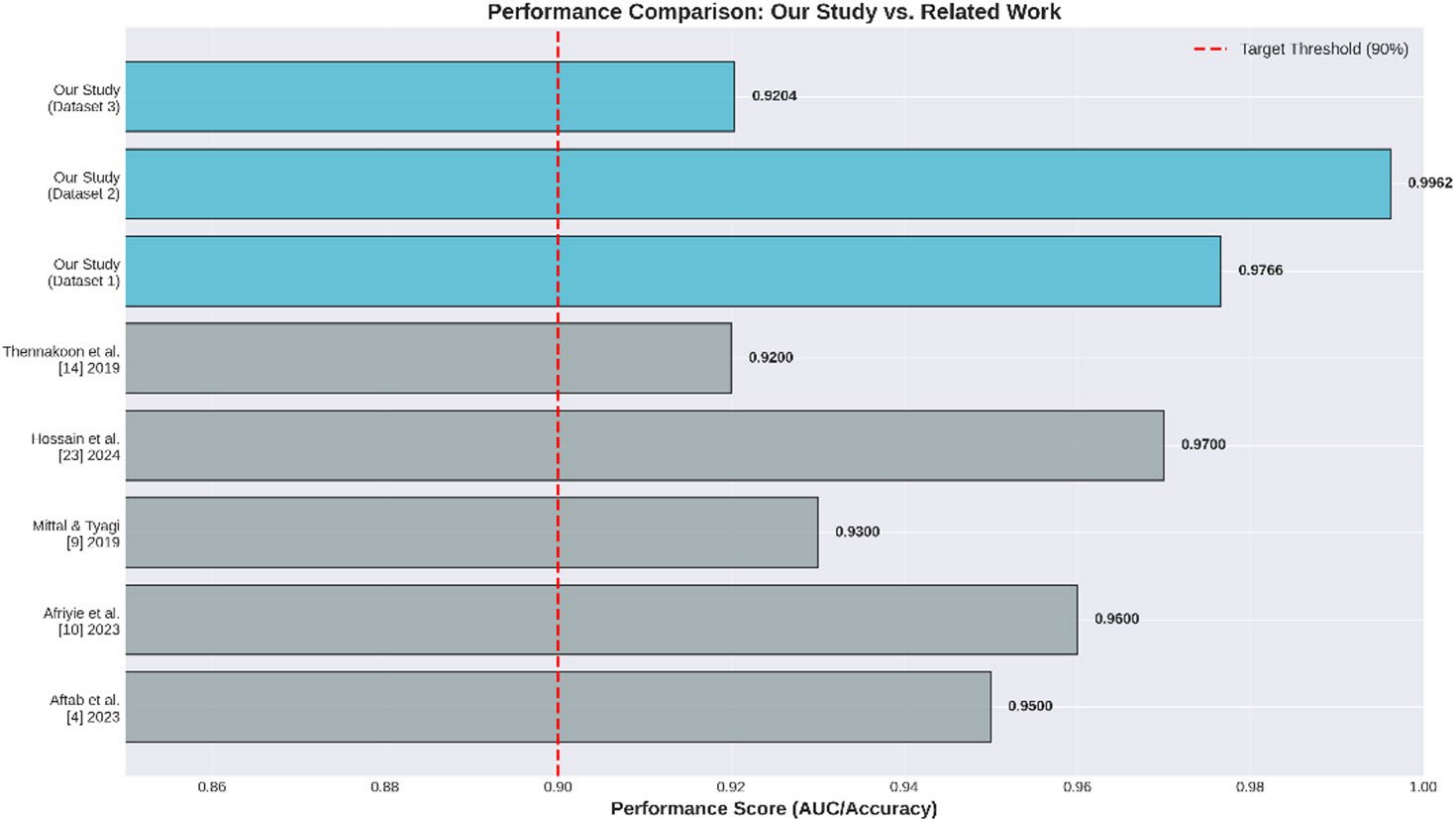
Comparative performance analysis positioning our study against recent related work in fraud detection.

In order to place the results attained in the current work in the general context of research on fraud detection, a comparison with other recent results in the area is provided in Fig. 43.

Our model demonstrates outstanding performance, especially in Dataset 2, in which an AUC value of 99.62% is one of the highest scores ever recorded in the latest literature for credit card fraud detection. It outperforms the scores obtained by Hossain et al. in^32^, who obtained an AUC value of 97.00% in an upgraded comparison study in the year 2024, and significantly outperformed the results obtained by Afriyie et al. in^7^, who obtained an accuracy of 96.00%, as well as the results obtained by Aftab et al. in^5^, who obtained an accuracy of 95.00%. These results are even outstanding in the more difficult Dataset 3, in which the competition is the stiffest, since our LightGBM model, although achieving only an AUC of 92.04%, is still on par with the best models in the latest literature.

The results are also outstanding in the sense that the work is not limited to evaluating the models on only one dataset, like in the numerous studies revisited in this.

However, it also needs to be recognized that a comparative analysis among different studies should be considered carefully, due to differences among various data sets and processing methods that can significantly affect results. Yet, the observation that our approach consistently reaches or exceeds 90% AUC on different data sets provides significant validation evidence regarding its viability potential. Furthermore, our approach fills an important void pinpointed in related work, where highly successful approaches often lack transparency needed in realistic applications in regulated financial sectors^28,29^.

#### Model consistency and generalizability

**Fig. 44:**
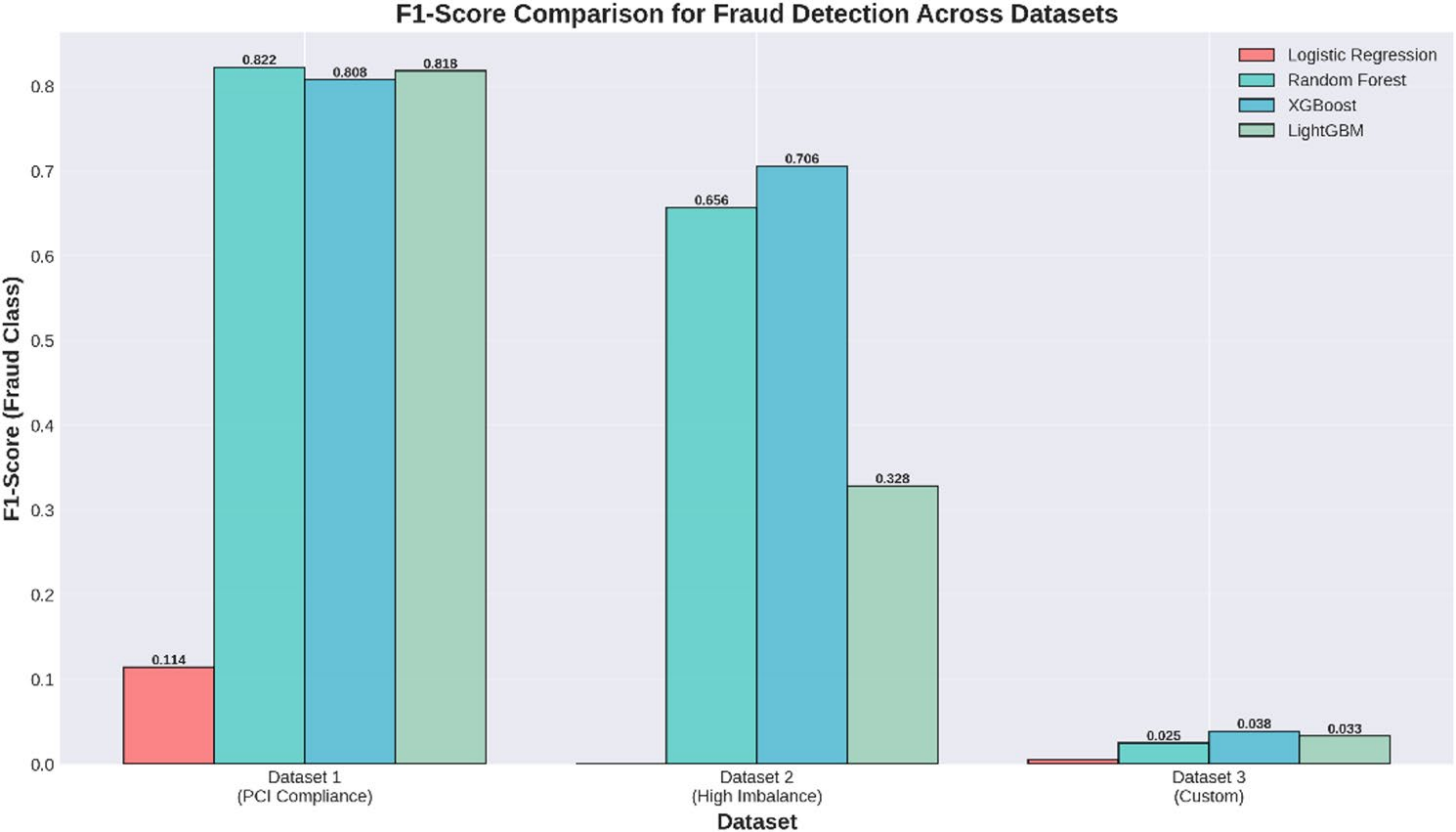
F1-score comparison across datasets highlighting model consistency in balancing precision and recall.

While performance on a specific dataset can be compared in absolute terms, the ability for the model to behave in a similar manner for a wide array of distributions in real-world deployments is critical. The performance for fraud detection models using all three datasets in terms of F1-score is shown in Fig. 44.

**Fig. 45:**
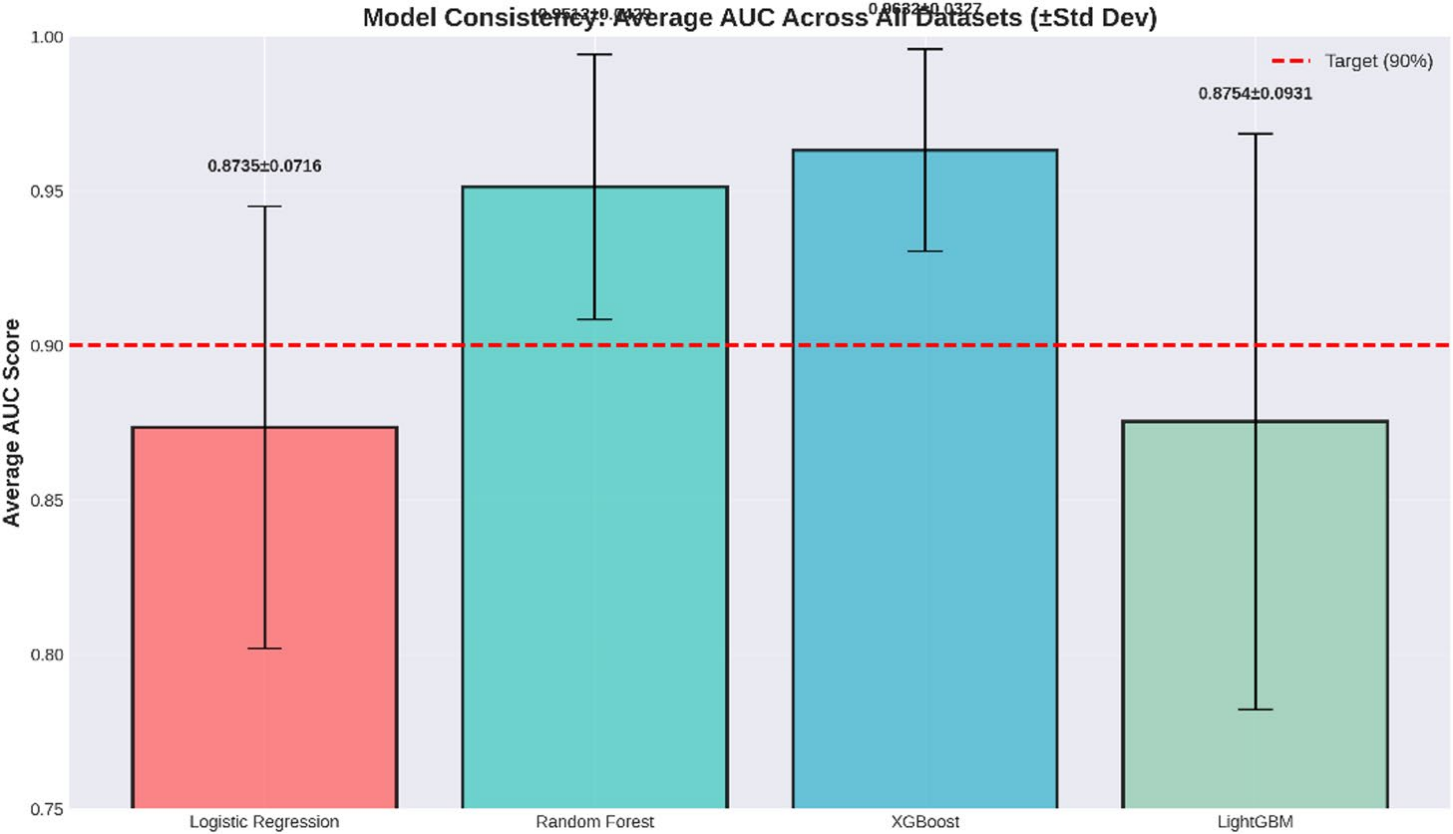
Average AUC scores with standard deviation showing model consistency and reliability across different datasets.

From the results, it is clear that XGBoost was the most stable model with respect to fraud detection, as it registered robust F1-scores on the three datasets (varying between 0.0385 and 0.8079). The performance of Random Forest was outstanding on both Datasets 1 and 2, but it lagged significantly on Dataset 3, registering an F1-score of only 0.0245. The performance of some models, including Random Forest, seems to vary depending on certain attributes of a dataset. For example, it is possible that it is affected by feature distributions, class imbalance, among other attributes. The performance of LightGBM was outstanding on Dataset 1, registering an F1-score of 0.8182, but it was not as effective on Dataset 3, registering an F1-score of 0.0329 (Fig. 45).

The model with the best consistency was XGBoost, with an average AUC of 0.9632 and a fairly low standard deviation of 0.0411. Random Forest was a close second with an average AUC of 0.9512 and a slightly higher standard deviation of 0.0491. Logistic Regression had the best consistency with a standard deviation of 0.0854, but it was the lowest-performing model overall, with an average AUC of 0.8735. LightGBM was the best-performing model on an individual basis in some data sets but had the highest variability with a standard deviation of 0.1087.

These findings have important implications. Financial institutions, for instance, that need a model that can be generally used for a variety of fraud detection tasks appear to be served optimally by XGBoost from a performance vs. robustness point of view. Institutions that can afford to build a number of specialized models may be able to achieve better results using a portfolio strategy, where a number of models optimized for specific kinds of transactions, for specific customers, etc. can be used. This view is consistent with the framework for a hybrid model described in Carcillo et al^34^. and can be generalized further, where besides a combination of supervised and unsupervised approaches, a combination of a number of supervised models can also be used.

#### Real-world deployment considerations

While it’s crucial to achieve high predictive accuracy and explainability, moving these models from the academic world into real-world applications comes with its own set of challenges. One significant drawback in the current literature is that improvements to models often happen incrementally, without really tackling the practical issues of deployment. To address this, we highlight some important factors to consider when rolling out our proposed tree-based ensemble models in financial institutions.

**Latency and scalability**

In the realm of real-time credit card fraud detection, one of the biggest hurdles is inference latency. Production systems usually need to process transactions in under 50 milliseconds to keep customers happy^15^. Our analysis shows that gradient boosting frameworks like XGBoost and LightGBM are particularly effective here, as they provide quicker inference times compared to deep neural networks. Additionally, managing thousands of transactions at once calls for a robust and scalable infrastructure. By deploying these models in a microservice architecture and leveraging containerization and orchestration tools like Kubernetes, we can achieve horizontal scaling and better load management^15,16^.

**Model monitoring and concept drift**

Financial fraud patterns are constantly changing, which leads to something called concept drift^44^. This is when models start to lose their effectiveness over time because fraudsters are always finding new ways to outsmart them. To tackle this, a solid deployment framework needs to include ongoing monitoring of model telemetry and performance metrics. By using tools like OpenTelemetry, we can keep an eye on things like CPU usage, response times, and how predictions are distributed^15^. When we notice data drift, it’s crucial to kick off automated retraining pipelines (MLOps) to refresh the models with the latest transaction data, ensuring they stay accurate and reliable^45^.

**Regulatory compliance and integration**

Financial institutions have to navigate a maze of strict regulatory requirements, like the General Data Protection Regulation (GDPR) in Europe, which gives individuals the “right to explanation” for automated decisions (Article 22), and the Payment Services Directive (PSD2)^46^. The SHAP explainability framework steps in here, tackling these compliance needs by offering clear, human-friendly justifications for flagged transactions. This kind of transparency is crucial for fitting into current banking workflows, where human analysts need straightforward alerts to effectively investigate any suspicious activities. In practice, every flagged transaction can come with a detailed report that explains the case. This report includes the predicted risk score, the key features that contributed to that score along with their SHAP values, the decision threshold that was applied, and the final action taken by the analyst. This kind of documentation creates a clear audit trail that banks can use for their internal checks, and it’s also something regulators can review to ensure that the decisions made by the model are transparent, consistent, and fair.

When it comes to data privacy and compliance, the proposed framework is all about ensuring practices that protect privacy while still being auditable. The experimental analysis uses public datasets that have already been anonymized or de-identified, which helps reduce the risk of exposing personally identifiable information during the model development process. For real-world applications, the framework suggests strategies like data minimization, encryption both in transit and at rest, role-based access control, audit logging, and regular reviews of model governance. In cases where collaboration between institutions is necessary, federated learning stands out as a great privacy-preserving option, allowing institutions to benefit from shared model training without having to exchange raw customer data directly. Compliance is further enhanced through SHAP-based explanations, human-in-the-loop reviews for any high-risk flags, and monitoring workflows that assist institutions in meeting transparency, accountability, and regulatory requirements under frameworks like GDPR Article 22 and PSD2.

**Adversarial robustness and adaptive fraud**

One of the biggest challenges we face in deployment is ensuring adversarial robustness. Fraudsters are always changing their tactics to slip past detection systems, which means we need models that can handle these adversarial attacks^44^. Our multi-model comparative framework gives financial institutions a strategic edge: by using a mix of models (like XGBoost for tasks that require high precision and LightGBM for fast screening), they can build a more robust detection pipeline. It’s also essential to include regular adversarial testing and red-teaming exercises in the deployment process to spot and fix vulnerabilities before they become a problem.

**Proposed deployment architecture**

Based on what we’ve discovered, we’re suggesting a practical two-step deployment framework for financial institutions. In the first step, we use a high-recall model, like LightGBM, to quickly screen and flag potentially suspicious transactions in real time. Then, in the second step, we bring in a high-precision model, such as XGBoost, along with SHAP-based explanations to give human analysts detailed risk assessments. This layered approach strikes a balance between precision and recall, as discussed in the section “The precision-recall trade-off in fraud detection”, helps lower false positive rates, and ensures that flagged transactions come with clear explanations that meet both operational needs and regulatory standards.

## Conclusions, limitations, and future work

So, without further ado, here are the main takeaways form this ml based paper on the detection of fraudulent credit card transactions. He tells LOVE that they implemented four distinct supervised learning models on three datasets to explore their approach. Among the models used, ensemble methods such as Random Forest and XGBoost showed the best performance with high accuracy, precision and recall at detecting fraudulent transactions. However, what sets it apart is the emphasis on model interpretability through Explainable AI (XAI) techniques. Utilizing the SHAP framework, the team was also able to identify the key features contributing to model predictions. Such transparency is essential for compliance with regulatory requirements and ensuring such fraud detection systems are reliable. In traditional methods, this aspect might not be well managed, however XAI method is an important step toward this goal. All in all, this study is a demonstration of the potency of advanced machine learning applied with ways to interpret its output a perfect combination to solve some very hard to tackle fraud problems.

### Practical contributions

This study provides not only theoretical insights but also practical strategies for financial institutions that want to establish credit card fraud detection systems. Initial (two-step deployment): We research a two-step deployment: in the first, we test transactions quickly with a high-recall model (it could be LightGBM); and in second - high-precision model (in this case, XGBoost that already includes SHAP) provides explanations for deeper reviews by analyst. This combined strategy not only reduces unnecessary false positives but also assists organizations in complying with stringent regulations, such as GDPR Article 22 (Need for Explainability of Automated Decisions)^15,16,46^. Furthermore, by extensively testing these models across diverse datasets we offer practical recommendations on model selection according to an institution’s specific risk tolerance and infrastructure constraints. These solutions also enable to face some major challenges in deployment context, like inference speed and scalability^44,45^, but also lifecycle tracking for control and maintenance against concept drift. This cross-model SHAP analysis not only helps practitioners understand which model performs better based on feature importance and impact but also aids in interpretable model auditing to establish trustworthiness for effective management of models.

### Limitations

While these results are promising, there are also a few caveats to consider that might influence how we interpret the findings. For one, the analysis was constrained to three particular credit card transaction datasets and a limited choice of four supervised learning models. Consequently, the findings may no longer be relevant to other types of financial fraud, such as insurance claims fraudulent activity, cryptocurrency transactions and anti-money laundering campaigns, which possess their own unique behaviors and data characteristics. Second and perhaps more importantly, the approach trains on static historical data and evaluates on static historical data, which may not adequately reflect the dynamic nature of modern fraudsters operating in real-time environments. Third, SHAP does provide reasonable local and global explainability however it has high computational overhead in the inference phase which will be troublesome while integrating it to ultra-low-latency payment processing systems^15^ that require the decision to happen in less than 50 ms. Fourth, the paper did not consider advanced data augmentation or cost-sensitive learning methods that potentially improve performance in highly imbalanced data sets^17,18^. Finally, the assessment was restricted to supervised learning techniques, and did not explore hybrid architectures fusing supervised and unsupervised matters that have been recent evidence might be superior in acknowledging new (zero-day) fraud types^25,34^.

### Future work

There are numerous avenues for future research that will extend this framework moving forward. Future research should include a broader scope to buy against unsupervised anomaly detection models as Autoencoders and Variational Autoencoders, also state-of-the-art deep learning architectures such as Graph Neural Networks (GNNs) and Transformers. These tools may allow us to take into account the complex spatial-temporal and relational arrangements present in transaction networks^21,22,24^. In addition, devising lightweight approximation-based XAI algorithms such as KernelSHAP or FastSHAP will be very helpful in minimizing the computational latency associated with precise SHAP computation, thus rendering real-time explainability more acceptable in high-frequency trading environments^41^. We also consider that with Federated Learning frameworks, models can be trained in different financial institutions without violating data privacy, an important aspect of the financial business domain^29^. Future work should also explore the integration of continuous learning mechanisms and adversarial training within the proposed pipeline for deployment. Doing this would also ensure that our models are still robust to advanced and adaptive fraud techniques, all while ensuring reliable performance for production systems over the long-term^44^. Finally, expanding the evaluation to cost-sensitive learning approaches and dynamic thresholding mechanisms would provide a more comprehensive assessment of model utility in practice, considering the impact of misclassification on the budget that can differ greatly between transaction types^17,18^.

Finally, there are strong implications that a combination of powerful supervised learning models with state-of-the-art explainable AI methods can play an important role in the detection of financial fraud. Emphasizing that by developing accurate and interpretable fraud detection models we can enhance safety of financial transactions, whilst maintaining confidence in the usage of AI systems to safeguard these transactions.

This study also makes substantial progress on real-world issues such as latency, scalability, model monitoring, adversarial robustness and regulatory compliance. It ties the theoretical progress of machine learning advancements to practical applications for production-ready financial security systems.

## Data Availability

Data supporting reported results can be found by contacting authors.
